# Risk factors for atopic and non-atopic asthma in school-age children from high-income and low- and middle-income countries

**DOI:** 10.1136/thorax-2024-222118

**Published:** 2025-06-24

**Authors:** Charlotte E Rutter, Harriet Mpairwe, Camila A Figueiredo, Mary Njoroge, Steven Robertson, Hajar Ali, Collin Brooks, Jeroen Douwes, Philip J Cooper, Martha Chico, Natalia Romero-Sandoval, Álvaro A Cruz, Pius Tumwesige, Mauricio L Barreto, Neil Pearce, Lucy Pembrey, the CAMERA Study Group, Jessica Alchundia

**Affiliations:** 1 Department of Medical Statistics, London School of Hygiene & Tropical Medicine, London, UK; 2 MRC/UVRI and LSHTM Uganda Research Unit, Entebbe, Uganda; 3 Institute of Health Sciences, Federal University of Bahia, Salvador, Brazil; 4 Clinical Trials Unit, London School of Hygiene & Tropical Medicine, London, UK; 5 Centre for Public Health Research, Massey University, Wellington, New Zealand; 6 Fundacion Ecuatoriana Para La Investigacion en Salud, Quito, Ecuador; 7 School of Medicine, Universidad Internacional del Ecuador, Quito, Ecuador; 8 Institute of Infection and Immunity, St George’s University of London, London, UK; 9 Grups de Recerca d’America i Africa Llatines (GRAAL), Barcelona, Spain; 10 PROAR, Federal University of Bahia, Salvador, Brazil; 11 Centre for Data and Knowledge Integration for Health (CIDACS), Fiocruz, Bahia, Brazil

**Keywords:** Asthma, Child, Paediatric asthma, Asthma Epidemiology

## Abstract

**Background:**

It is well established that there are different asthma phenotypes, but whereas determinants of atopic asthma (AA) are well studied, little is known about non-atopic asthma (NAA). We compared risk factors for atopy, AA in atopics and NAA in non-atopics in children in a wide variety of countries.

**Methods:**

Using four studies, across 23 countries, we assessed asthma status and atopy (skin prick tests) for children aged 6–17, plus risk factors from housing, heating, pets, family, diet and air quality categories. Using mixed-effects logistic regression models, we assessed risk factors over four pathways: pathway 1—non-atopic non-asthma to NAA; pathway 2—non-atopic non-asthma to atopy (no asthma); pathway 3—atopic non-asthma to AA; pathway 4—non-atopic non-asthma to AA. We compared the log odds of risk factors between pathways using the Pearson correlation coefficient (PCC).

**Results:**

Our final sample of 32 741 children comprised 67% with neither atopy nor asthma, 15% with atopy but without asthma, 8% with AA and 10% with NAA. Risk factors were similar between pathway 1 and pathway 3 (PCC=0.81, 95% CI 0.68 to 0.94). In contrast, risk factors differed between pathway 2 and pathway 3 (PCC=−0.06, 95% CI −0.29 to 0.17).

**Discussion:**

These findings indicate that although atopy increases the risk of asthma, the risk factors for subsequently developing asthma are generally the same in those with and without atopy. This raises important questions about the role of atopy in asthma, particularly whether it is an inherent part of the aetiological process or is coincidental.

**WHAT IS ALREADY KNOWN ON THIS TOPIC**

- It is well established that there are different phenotypes of asthma, but little is known about risk factors for non-atopic asthma.

**WHAT THIS STUDY ADDS**

- Using a novel approach, we found that lifestyle and environmental risk factors for developing asthma are generally similar in atopic and non-atopic children, but the risk factors for atopy are quite different.

**HOW THIS STUDY MIGHT AFFECT RESEARCH, PRACTICE OR POLICY**

- Our findings suggest that atopy and asthma may be coincidental in a large proportion of children who are defined as having atopic asthma. This has important implications for our understanding of the causes and mechanisms of different asthma phenotypes, and therefore prevention and treatment of asthma.

## Introduction

 Although the importance of intrinsic (or non-allergic or non-atopic) asthma has been recognised since the early 20th century,^1^ the focus of asthma research in the past 60 years has been predominantly on allergic mechanisms despite compelling evidence that at most half of asthma cases are attributable to atopy.^2 3^

As a consequence, little is known about the causes and pathophysiological mechanisms of non-atopic asthma (NAA), hampering the development of effective prevention and treatment options for a large proportion of people with asthma.

Atopic asthma (AA) is associated with T helper type 2-mediated airway inflammation, with atopy often identified (with varying results) by skin prick test (SPT) positivity to common allergens or serum allergen-specific immunoglobulin E (IgE) measurement (commonly associated with airway eosinophilia).^4^ The mechanisms underlying NAA remain unclear but may involve neutrophilic inflammation, neural mechanisms, infection-related responses or other currently unknown pathophysiological pathways.^5^,^7^ Different mechanisms may lead to different risk factors associated with disease. To make a valid comparison between NAA and AA, we first need to tease apart the risk factors for asthma from the risk factors for atopy.

**Figure 1:**
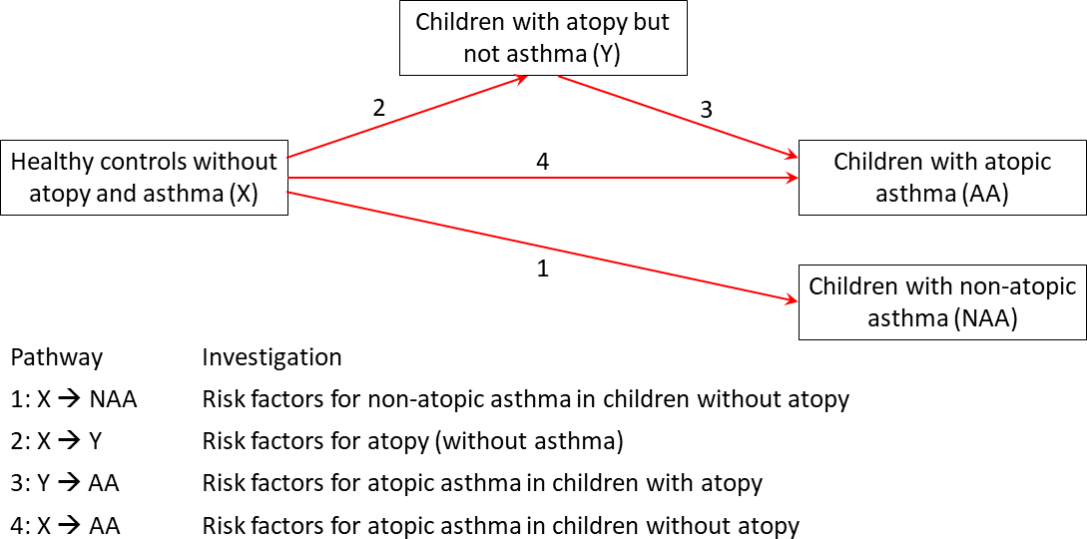
Pathways of possible asthma causation (adapted from Barreto *et al* 8).

Figure 1 shows a framework for pathways potentially involved in the development of AA and NAA.^8^ These are not often considered separately in studies of risk factors. Instead, most have done one of the following^8^: (1) compared all asthma cases with all non-asthmatic controls (a mix of all four pathways); (2) adjusted for atopy (an average of the effects of pathway 1 and pathway 3); (3) compared AA with all non-asthmatic controls (a mix of pathways 2–4); (4) compared NAA with all non-asthmatic controls (none of the indicated pathways); or (5) a few studies have stratified on atopy (yielding separate estimates for pathways 1 and 3).

A 2014 review of the evidence for NAA risk factors found that, of 30 risk factors evaluated in 43 studies, only three (family history of allergic disease, household dampness/mould and childhood lower respiratory tract infections) showed consistent associations with NAA. A lack of breastfeeding, or breastfeeding for a limited period, was less consistently associated with NAA. However, this review noted that most of these studies used inappropriate comparison groups.^9^ There was also considerable methodological heterogeneity with different definitions of atopy and asthma, and different study designs, hampering valid comparisons.^9^

Here, using data from four studies: the International Study of Asthma and Allergies in Childhood (ISAAC) Phase II, the World Asthma Phenotypes (WASP), the Social Change, Asthma and Allergy in Latin America (SCAALA) and the Avon Longitudinal Study of Parents and Children (ALSPAC), we aimed to separately assess and compare risk factors for the four pathways (figure 1), allowing the identification of modifiable factors that may increase/decrease the risk of NAA (pathway 1) and/or AA (pathways 2–4).

## Methods

### Data sources

The ISAAC Phase II cross-sectional questionnaire survey (with additional objective tests) commenced in 1998, targeting children aged 10–11, and included 30 centres from a mix of high-income countries (HIC) and low- and middle-income countries (LMICs). Detailed methods have been published previously.^10 11^

The WASP cross-sectional case–control study was conducted in five centres in HICs (UK, New Zealand (NZ)) and LMICs (Brazil, Ecuador, Uganda) between 2017 and 2020. Participants in Brazil, Ecuador and NZ were recruited from ongoing population-based cohort studies, with additional recruitment through the community (eg, surveys in schools). In Uganda, participants were recruited from a larger case–control study of children with and without asthma identified through a cross-sectional survey in schools. Full details of the questionnaire and methods have been published previously.^4^ The UK centre surveyed young adults (26–27 years) and has therefore been excluded from this analysis.

The SCAALA study involved a cross-sectional survey in Ecuador and a longitudinal study in Brazil, with detailed protocols published previously. In Ecuador, data were collected in 2006 on children aged 5–16 years in the Esmeraldas province.^12^ In Brazil, data were collected in 2005 on children aged 4–11 years at baseline in the city of Salvador, Northeastern Brazil.^13^

For the ALSPAC birth cohort study, pregnant women resident in Avon, UK, with expected dates of delivery between 1 April 1991 and 31 December 1992 were invited to take part in the study. The initial number of pregnancies enrolled was 14 541 from which 13 988 children were still alive at 1 year of age, consisting of 14 203 unique women (G0 mothers). Information was collected from 12 113 G0 partners (of the mothers), of which 3807 are currently enrolled on the study. After 7 years, efforts were made to find eligible pregnancies that were missing from the study. The total sample size for analyses using any data collected after the age of 7 is therefore 15 447 pregnancies, from which 14 901 children were still alive at 1 year of age. The study included regular surveys and tests throughout childhood, details of which have been reported previously.^14 15^ Here we use SPT data at age 7 and surveys collected up to age 7. Please note that the study website contains details of all the data that are available through a fully searchable data dictionary and variable search tool.^16^

From all sources, individuals with missing data on symptoms, SPT results, age or sex were excluded, along with those outside the target age group of 6–17 years, and those who did not fit into a category of case or control (ie, with previous asthma symptoms/diagnosis but not in the past year). Missingness was checked for associations with demographics.

### Variable definitions

Children were defined as having current asthma if they had wheeze symptoms and/or used asthma medications in the past 12 months. They were defined as without asthma if they never had asthma or asthma symptoms, determined by negative responses to questions on ever having wheeze symptoms or diagnosed asthma (ISAAC and SCAALA) or by negative responses to questions on recent wheeze symptoms across all surveys in ALSPAC. Participants qualifying for neither status (eg, those who previously had asthma) were excluded from the analyses. More details on the exact definitions for each study are found in online supplemental table S1.

We defined atopy as SPT positivity (≥3 mm wheal in ISAAC, WASP and SCAALA, ≥2 mm wheal in ALSPAC) to at least one of a panel of five or more common allergens. Individuals testing positive for saline (negative control), negative for histamine (positive control) or with fewer than five allergens tested were excluded. Similar SPT methods were used in each study, and allergens appropriate for the local environment of each centre were used (online supplemental table S2).

Study participants were categorised into four outcome groups: healthy controls (no atopy and no asthma) (X), those with atopy but no asthma (Y), those with AA and those with NAA.

We included potential risk factors if they were available in ISAAC as it was by far the largest study. These were all binary variables and included housing condition (heating, cooling, insulation, cooking fuel, feather/synthetic bedding, damp), animal contact (pet cats, dogs, birds and farm animals), diet (meat, fish, fruit, vegetables, fast food), body mass index category, family (siblings, parental allergies/education/smoking) and early life factors (breastfeeding, birth weight, prematurity) (online supplemental table S1).

### Statistical analyses

Four sets of models were used, representing each pathway in figure 1.

Pathway 1: comparing those with NAA to healthy controls (risk factors for NAA).

Pathway 2: comparing those with atopy but without asthma to healthy controls (risk factors for atopy).

Pathway 3: comparing those with AA to those with atopy but not asthma (risk factors for AA).

Pathway 4: comparing those with AA to healthy controls (risk factors for AA and atopy together).

For the first three pathways, we examined which risk factors were associated with NAA (figure 1, pathway 1), atopy (pathway 2) and AA in atopic children (pathway 3). Pathway 4 was more difficult to address as it is in addition to the two existing pathways, 2 and 3. It was assessed by estimating the overall association of non-atopic non-asthma (X) with AA (not adjusted or stratified on atopy) and comparing it with that predicted assuming no direct path, that is, the OR for pathway 2 multiplied by the OR for pathway 3. A difference between the two figures was considered to indicate a possible direct path (assuming the difference was not due to misclassification or residual confounding). In addition, we directly compared AA to NAA to see which factors differentiated them and how these compared with the risk factors for atopy alone.

Mixed-effects logistic regression models were fitted on the binary outcome representing each pathway, with a random intercept for centre and adjusting for age, sex, study and country income level (LMIC/HIC). In children, asthma prevalence is known to increase with age, peaking around 13–14 years, after which it decreases.^17^ Thus, using the models with no risk factors, we first tested for evidence against a linear trend for age by adding a quadratic term and then by adding a restricted cubic spline (RCS); the most appropriate form was then used for the models of each pathway.

Individual models were fitted for each risk factor in turn, and results compared between pathways. The sample size varies between models as the amount of missing data for each risk factor varies. In addition, some risk factors were not included for a particular study; as stated previously, all included risk factors were in the ISAAC study and at least one other study (online supplemental table S1). To assess the relationship between risk factors for each pathway, we plotted the OR estimates for one pathway against another (on the log scale as the log odds is usually normally distributed) and calculated the Pearson correlation coefficient (PCC) for the log odds and associated bootstrapped CIs (using bootstrap samples of the individual-level dataset to account for the uncertainty of the individual estimates as well as the uncertainty of the correlation) for 1000 replications.

Models stratified by country income group were fitted to test for interactions, and separate models were fitted for each study to assess consistency.

Sensitivity analyses were conducted on the main non-stratified models as follows:

1. To exclude borderline atopics, two stricter atopy definitions were applied to data from ISAAC and ALSPAC (due to data availability): any wheal of at least 5 mm in the core allergens; two or more wheals of at least 4 mm in the core allergens. Those originally classed as atopic but not meeting the new definition were excluded from that analysis. The definition for non-atopics remained the same. Results were compared with those using the standard atopy definition but limited to the two studies.
2. To check if there was an imbalance between studies, four analyses were conducted leaving out one study each time.
3. Lastly, an analysis was conducted that used a more lenient definition for the controls, allowing for a small amount of missing symptom data (either one past questionnaire missing for ALSPAC or the question ‘Has the child ever been told they have asthma?’ missing in ISAAC).

Data were analysed using Stata V.18.^18^

## Results

From a total sample of 71 916 participants (1205 from WASP, 54 271 from ISAAC, 8266 from SCAALA and 8174 from ALSPAC), 33 149 were deemed ineligible for either being out of the age range, not being offered an SPT, or previously having asthma symptoms but not in the past year. This left a sample of 38 767 participants to be considered for analysis. Of these, 6026 (16%) were excluded due to missing data: demographics (21); SPT data (1705); and symptom data (4300) (table 1). There were some relatively weak associations between missing data and demographics; being male was associated with missing SPT data (OR=1.15, 95% CI 1.03 to 1.29), but not with missing symptom information. Older children were associated with a higher chance of missing SPT data (1.07 (1.02 to 1.13)) but a much lower chance of missing symptom information (0.63 (0.60 to 0.65)). Individuals with current asthma were more likely to be missing the SPT data than those who never had asthma (1.27 (1.09 to 1.47)) (online supplemental table S3).

**Table 1:** Exclusions

	WASP	ISAAC	SCAALA	ALSPAC	Total
Total sample	1205	54 271	8266	8174*	71 916
Out of age range	177	864	553	93	1687
No SPT offered	0	21 801	0	478	22 279
Previous but not current asthma symptoms	84	4396	2175	2528	9183
Total ineligible	261	27 061	2728	3099	33 149
Eligible sample	944	27 210	5538	5075	38 767
Missing sex/age	0	9	0	12	21
Missing SPT data	21	0	216	1468	1705
Missing symptoms	0	3545	0	755	4300
Total missing data	21	3554	216	2235	6026
Final sample	923	23 656	5322	2840	32 741

* Individuals who completed the age 7 questionnaire, without second twin.

ALSPAC, Avon Longitudinal Study of Parents and Children; ISAAC, International Study of Asthma and Allergies in Childhood; SCAALA, Social Change, Asthma and Allergy in Latin America; SPT, skin prick test; WASP, World Asthma Phenotypes.

The final sample of 32 741 comprised 66% healthy controls, 15% children with atopy but not asthma, 8% with AA and 10% NAA. The WASP sample had a higher proportion of children with asthma (75%) than the others (15–30%) due to its case–control design (table 2).

**Table 2:** Distribution of main outcome by study

Study	Healthy controls, no atopy, no asthma (X) (%)	Atopy but no asthma (Y) (%)	Atopic asthma (AA) (%)	Non-atopic asthma (NAA) (%)	Total
WASP	171 (19)	66 (7)	422 (46)	264 (29)	923
ISAAC	16 239 (69)	3922 (17)	1584 (7)	1911 (8)	23 656
SCAALA	3860 (73)	560 (11)	203 (4)	699 (13)	5322
ALSPAC	1650 (58)	322 (11)	433 (15)	435 (15)	2840
Total	21 920 (67)	4870 (15)	2642 (8)	3309 (10)	32 741

ALSPAC, Avon Longitudinal Study of Parents and Children; ISAAC, International Study of Asthma and Allergies in Childhood; SCAALA, Social Change, Asthma and Allergy in Latin America; WASP, World Asthma Phenotypes.

Participants in WASP were on average the oldest but with a wide range (9–18 years). Those in ALSPAC were 7–8 years, ISAAC participants were mainly 10–11 years and SCAALA ranged from 6 years to 16 years (online supplemental figures S1–S3). The WASP dataset was 55% female, SCAALA was 48% and ISAAC and ALSPAC were both 51%. In WASP, 64% of the individuals were from LMICs, and in ISAAC this was 32%. SCAALA participants were all from LMICs (Brazil and Ecuador) and ALSPAC were all from HICs (UK). Details of the final sample by country are found in online supplemental table S4.

The prevalence of each risk factor by study is presented in table 3 (expanded to include individual centres in online supplemental table S5). Each risk factor was available for a different subset of individuals, ranging from 14 060 for synthetic pillows to 31 175 for parental allergic diseases. There was considerable variation between centres for some variables, particularly indoor heating questions, as some centres in hot countries do not use heating at all.

**Table 3:** Prevalence of potential risk factors by study

Category	Covariable	n	ISAAC	WASP	SCAALA	ALSPAC	Overall total
Study sample size			23 656	923	5322	2840	32 741
Confounder	Male sex	32 741	49%	45%	52%	49%	49%
House/bedding	Feather bedding	14 156	18%	25%	–	–	18%
	Synthetic bedding	14 155	43%	35%	–	–	43%
	Blankets	15 560	44%	45%	22%	–	43%
	Feather pillow	14 780	17%	32%	–	–	17%
	Synthetic pillow	14 060	39%	47%	–	–	40%
	Double glazing	20 831	55%	–	–	81%	58%
	Double glazing in 1st year	20 194	45%	–	–	59%	47%
	Urban	23 452	92%	–	45%	88%	81%
	Urban in 1st year	16 953	89%	–	–	93%	89%
Heating/cooking	Heating	16 979	68%	48%	–	–	67%
	Wood heating	18 386	14%	14%	–	–	14%
	Coal/coke heating	17 603	4%	0%	–	–	4%
	Gas heating	16 261	28%	34%	–	–	28%
	Heating in 1st year	15 523	68%	–	–	–	68%
	Wood heating in 1st year	16 894	20%	–	–	–	20%
	Coal/coke heating in 1st year	16 330	6%	–	–	–	6%
	Cooking electric	20 728	45%	11%	0%	–	43%
	Cooking gas	29 549	54%	77%	97%	61%	63%
	Cooking gas in 1st year	18 776	19%	–	99%	56%	27%
Animals	Pets	20 342	43%	58%	57%	70%	51%
	Cats	30 397	15%	31%	33%	30%	20%
	Dogs	30 379	14%	36%	42%	20%	20%
	Birds	24 130	14%	–	–	6%	13%
	Cat in 1st year	21 091	9%	–	7%	29%	12%
	Dog in 1st year	21 091	8%	–	18%	19%	10%
	Bird in 1st year	20 399	7%	–	–	5%	7%
	Farm animals	22 052	7%	60%	22%	–	11%
Diet/weight	Meat weekly	26 025	88%	95%	85%	–	87%
	Seafood weekly	26 507	57%	69%	81%	–	62%
	Green vegetables weekly	24 540	83%	–	68%	78%	79%
	Fruit weekly	25 019	90%	93%	87%	96%	90%
	Fast food weekly	17 878	25%	42%	7%	–	20%
	Fruit juice weekly	18 553	69%	–	55%	69%	69%
	Overweight	14 615	25%	22%	10%	14%	18%
	Obese	14 615	7%	5%	3%	3%	4%
Family/birth	Older siblings	29 536	52%	–	75%	52%	56%
	Younger siblings	29 470	49%	–	72%	52%	53%
	More than 1 sibling	29 528	40%	–	82%	34%	47%
	Ever breastfed	29 588	78%	–	97%	80%	82%
	Low birth weight	20 176	18%	–	–	4%	16%
	Premature	16 875	16%	–	8%	9%	14%
	Parental allergic diseases	31 175	36%	–	40%	74%	39%
	Maternal education	18 189	79%	–	34%	100%	70%
	Paternal education	18 389	80%	–	46%	100%	74%
Air quality	Any smokers	29 371	45%	–	41%	29%	43%
	Maternal smoking	30 869	25%	–	13%	16%	22%
	Maternal smoking in 1st year	27 364	19%	–	11%	17%	17%
	Maternal smoking during pregnancy	28 545	12%	–	10%	16%	12%
	Damp	17 670	14%	28%	–	90%	20%
	Mould	15 021	9%	39%	–	52%	15%
	Damp or mould	15 512	18%	49%	60%	92%	28%
	Damp in 1st year	17 763	15%	–	–	49%	20%
	Mould in 1st year	14 599	10%	–	–	25%	13%
	Air conditioning	14 910	29%	3%	1%	–	27%

ALSPAC, Avon Longitudinal Study of Parents and Children; ISAAC, International Study of Asthma and Allergies in Childhood; SCAALA, Social Change, Asthma and Allergy in Latin America; WASP, World Asthma Phenotypes.

Results from the mixed-effects logistic regression, with random intercepts for centre and adjusted for study, sex, age and country income level (LMIC/HIC), but without including risk factors, showed evidence that the effect of age was not linear for pathway 1 (quadratic p=0.046, RCS p=0.17) and for the AA/NAA comparison (quadratic p=0.016, RCS p=0.23), but there was no evidence against linearity for pathways 2–4 (quadratic p=0.27, p=0.18, p=0.45 and RCS p=0.43, p=0.87, p=0.87, respectively). Thus, a quadratic age term was included in all models for pathway 1 and the AA/NAA comparison.

**Figure 2:**
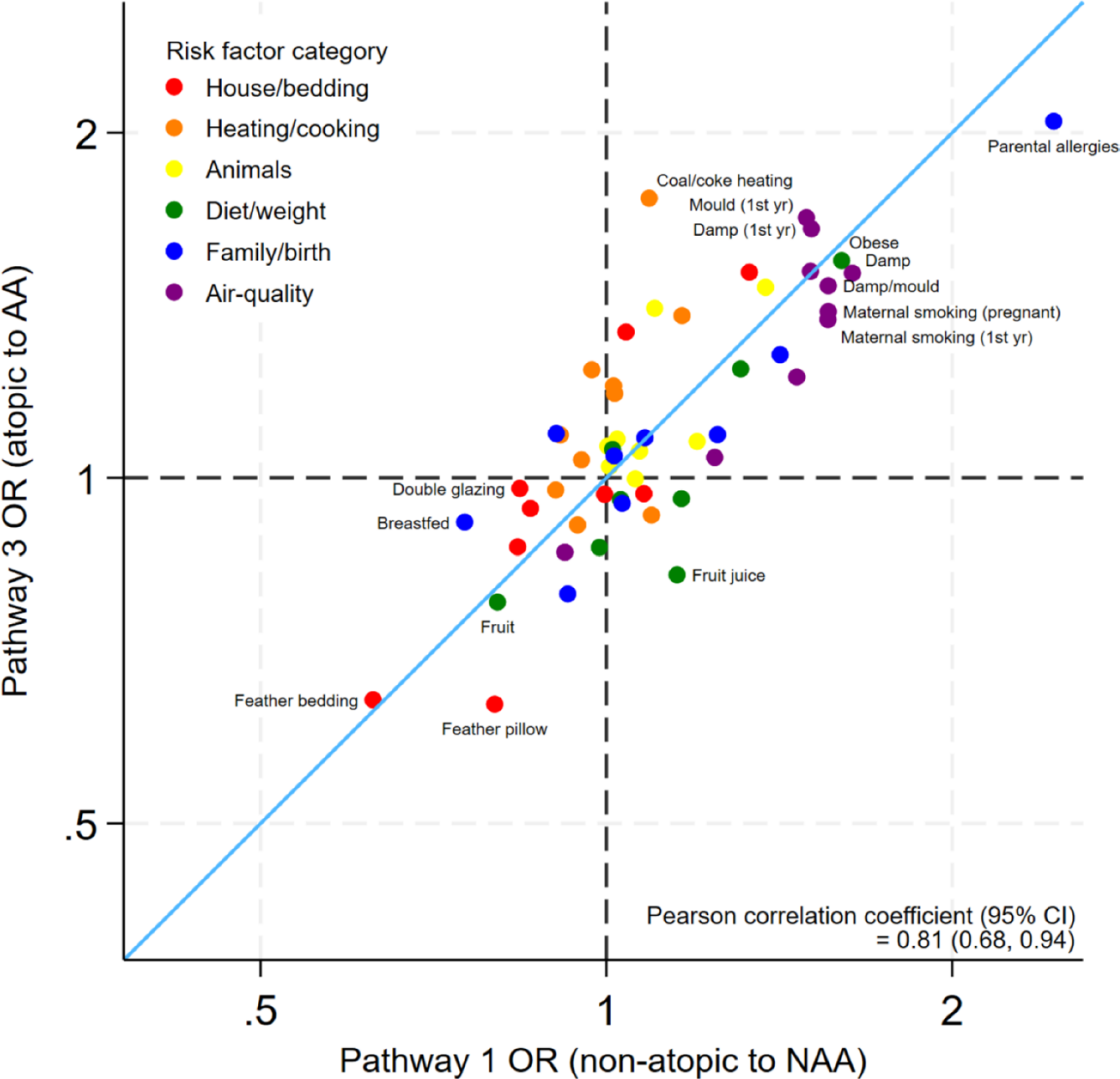
Estimated ORs of risk factors for atopic asthma (AA) compared with non-atopic asthma (NAA), excluding atopy risk (pathway 3 vs pathway 1). Axes are shown on the natural log scale.

Figure 2 compares the estimated ORs of each risk factor between AA in already atopic individuals (pathway 3) and the risk of NAA in non-atopic individuals (pathway 1). The associations for the included risk factors were generally similar for AA and NAA, with a high positive correlation between the estimated log odds (PCC=0.81, bootstrapped 95% CI 0.68 to 0.94).

**Figure 3:**
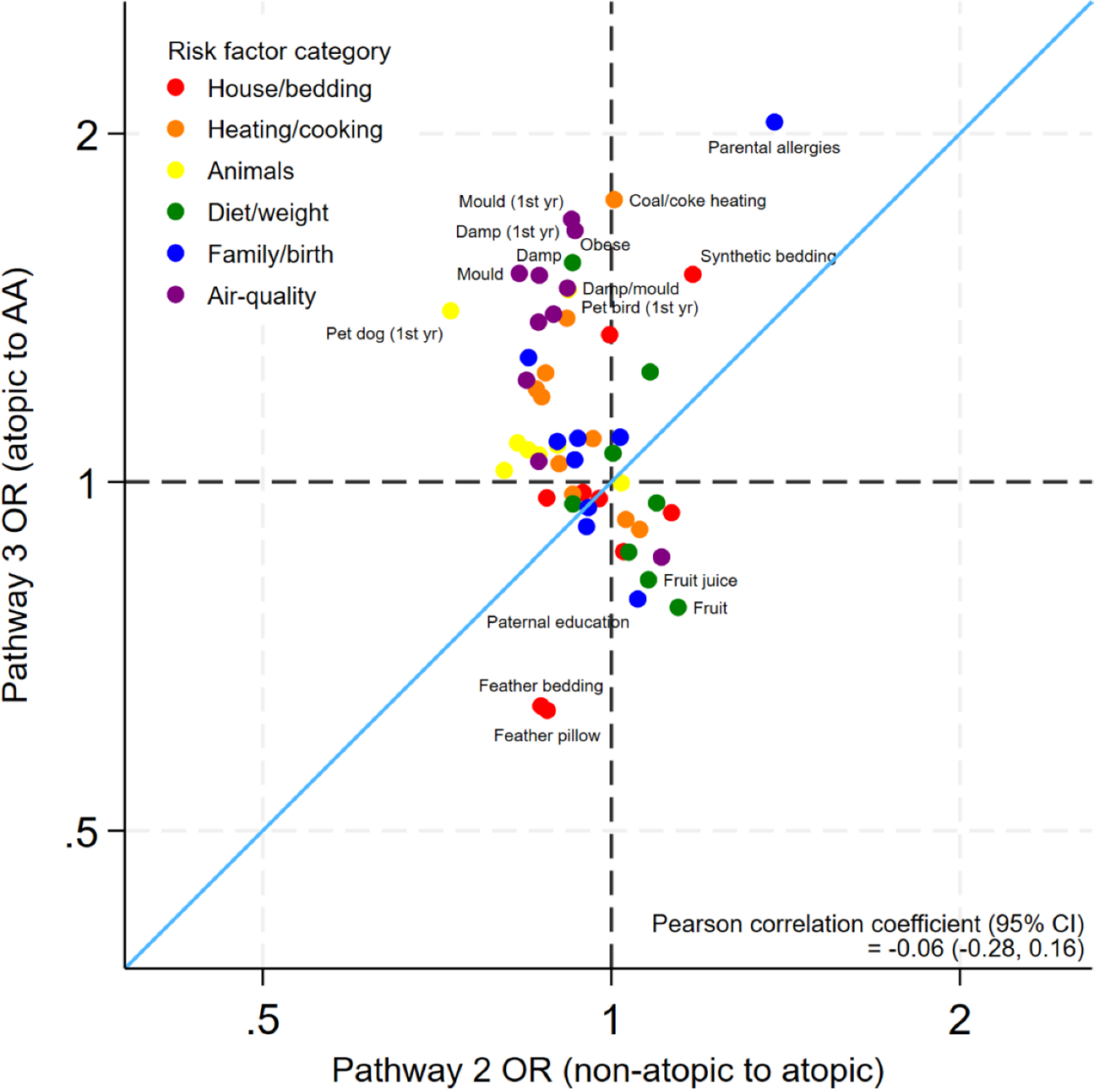
Estimated ORs of risk factors for asthma (in atopic individuals) compared with atopy (pathway 3 vs pathway 2). Axes are shown on the natural log scale. AA, atopic asthma.

Figure 3 compares risk factors for atopy (pathway 2) with risk factors for AA in atopic individuals (pathway 3) with no correlation between the two groups (−0.06 (−0.28 to 0.16)).

We also compared the risk factors for atopy (pathway 2) with the risk factors for AA compared with NAA, as in both of these groups the difference is atopy. We found a moderate positive correlation (0.54 (0.34 to 0.74)) between the risk factor log odds of these groups (online supplemental figure S4).

The individual results for each risk factor and outcome are shown in online supplemental table S6, along with predictions of pathway 4, based on the results for pathways 2 and 3. The observed and the predicted values are similar, so there is likely no direct pathway 4 (non-atopic to AA) over and above the two separate pathways. Results comparing pathways 1–3 by category of risk factor are shown in online supplemental figures S5–S10.

The strongest risk factor for asthma was parental history of allergic disease (asthma, eczema or hay fever) (NAA OR=2.45 (95% CI 2.24 to 2.69); AA 2.05 (1.81 to 2.31)). Breastfeeding (ever) was associated with a lower risk of NAA (0.75 (0.67 to 0.85)). Premature birth (1.42 (1.19 to 1.69)) and low birth weight (1.25 (1.05 to 1.49)) were associated with an increased NAA risk (online supplemental figure S9).

Bedding-related risk factors included feather bedding (NAA 0.63 (0.52 to 0.76); atopy 0.87 (0.76 to 0.99); AA 0.64 (0.52 to 0.79)) and feather pillows (NAA 0.80 (0.66 to 0.96); atopy 0.88 (0.77 to 1.01); AA 0.64 (0.50 to 0.81)) which were both associated with a lower risk of asthma and atopy across all pathways. Conversely, synthetic bedding was associated with a higher risk (NAA 1.33 (1.16 to 1.53); atopy 1.18 (1.05 to 1.31); AA 1.51 (1.28 to 1.79)) (online supplemental figure S5).

Current use of coal/coke heating was associated with an increased risk of developing AA among atopic individuals (1.75 (1.11 to 2.78)), but other heating factors did not show a strong association with asthma or atopy (online supplemental figure S6).

There was evidence that having pet birds in the first year of life was associated with higher risk of asthma (NAA 1.38 (1.13 to 1.68); AA 1.47 (1.15 to 1.87)), and having pet dogs in the first year of life was associated with a higher risk of AA (1.41 (1.15 to 1.72)). Many types of pets (both current and in first year of life) were associated with a lower risk of atopy (any pets 0.87 (0.79 to 0.95); cats 0.81 (0.74 to 0.89); dogs 0.90 (0.82 to 0.99); cats in first year 0.83 (0.72 to 0.95); dogs in first year 0.73 (0.63 to 0.84)). Farm animal contact showed no association with either asthma or atopy (online supplemental figure S7).

Obesity (NAA 1.60 (1.28 to 2.01); AA 1.55 (1.10 to 2.16)) and being overweight (NAA 1.31 (1.14 to 1.50); AA 1.24 (1.03 to 1.51)) were associated with higher risk of both forms of asthma but were not related to risk of atopy alone. Regular fruit consumption was associated with a lower risk of asthma and a weak but increased risk of atopy (NAA 0.80 (0.70 to 0.93); atopy 1.14 (0.99 to 1.31); AA 0.78 (0.63 to 0.97)) (online supplemental figure S8).

Almost all indoor air-related factors including damp (NAA 1.64 (1.41 to 1.90); atopy 0.87 (0.75 to 1.00); AA 1.51 (1.22 to 1.86)), mould (NAA 1.51 (1.29 to 1.75); atopy 0.83 (0.69 to 1.00); AA 1.51 (1.20 to 1.91)) and maternal smoking (current: NAA 1.46 (1.32 to 1.62); atopy 0.84 (0.78 to 0.92); AA 1.22 (1.07 to 1.40); in first year: NAA 1.56 (1.40 to 1.74); atopy 0.87 (0.78 to 0.96); AA 1.37 (1.18 to 1.60)) were associated with an increased risk of asthma (NAA and AA) and a slight protective effect on atopy (online supplemental figure S10).

**Figure 4:**
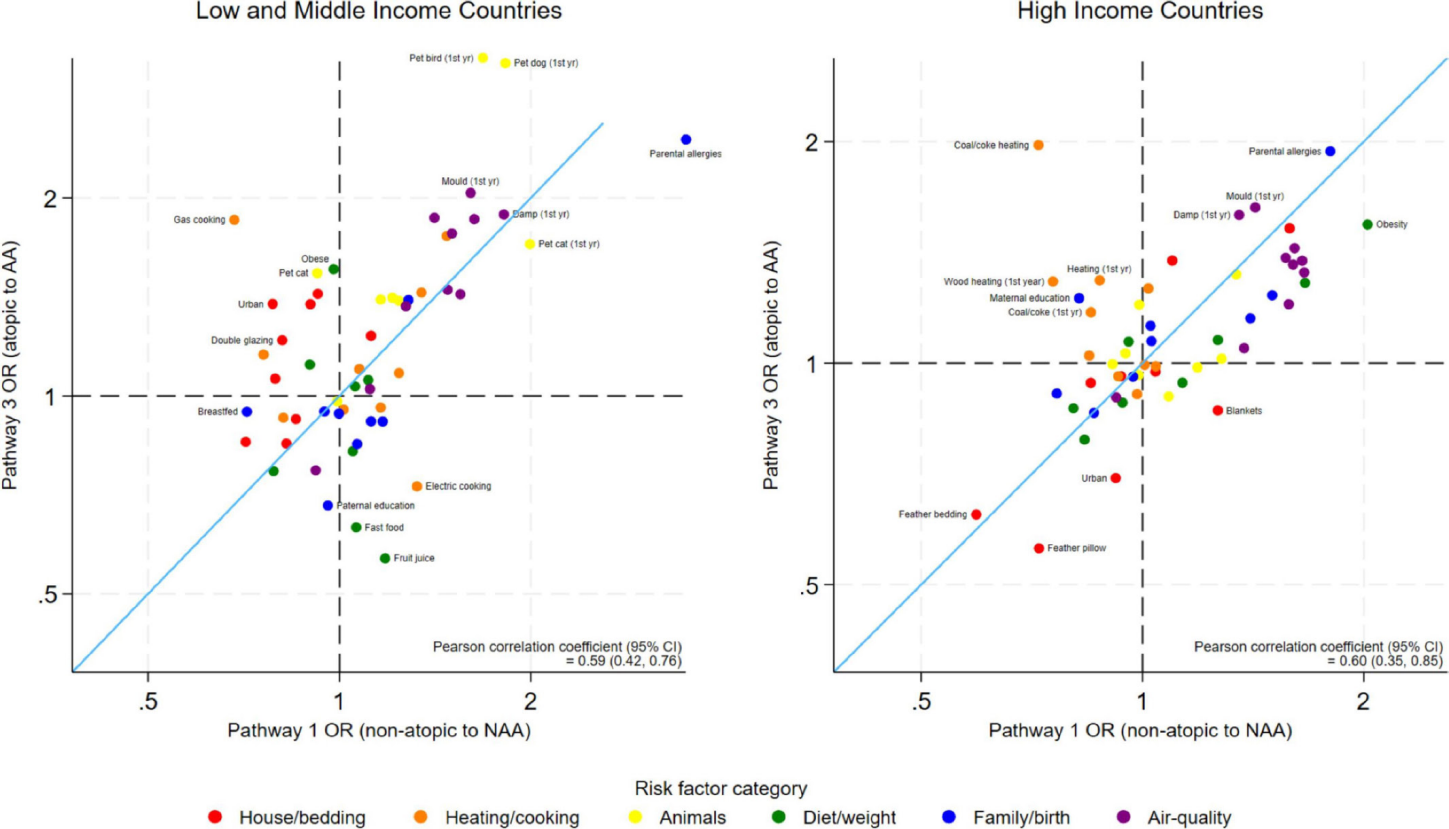
Estimated ORs of risk factors for atopic asthma (AA) compared with non-atopic asthma (NAA), excluding atopy risk (pathway 3 vs pathway 1) stratified by country affluence. Axes are shown on the natural log scale.

**Figure 5:**
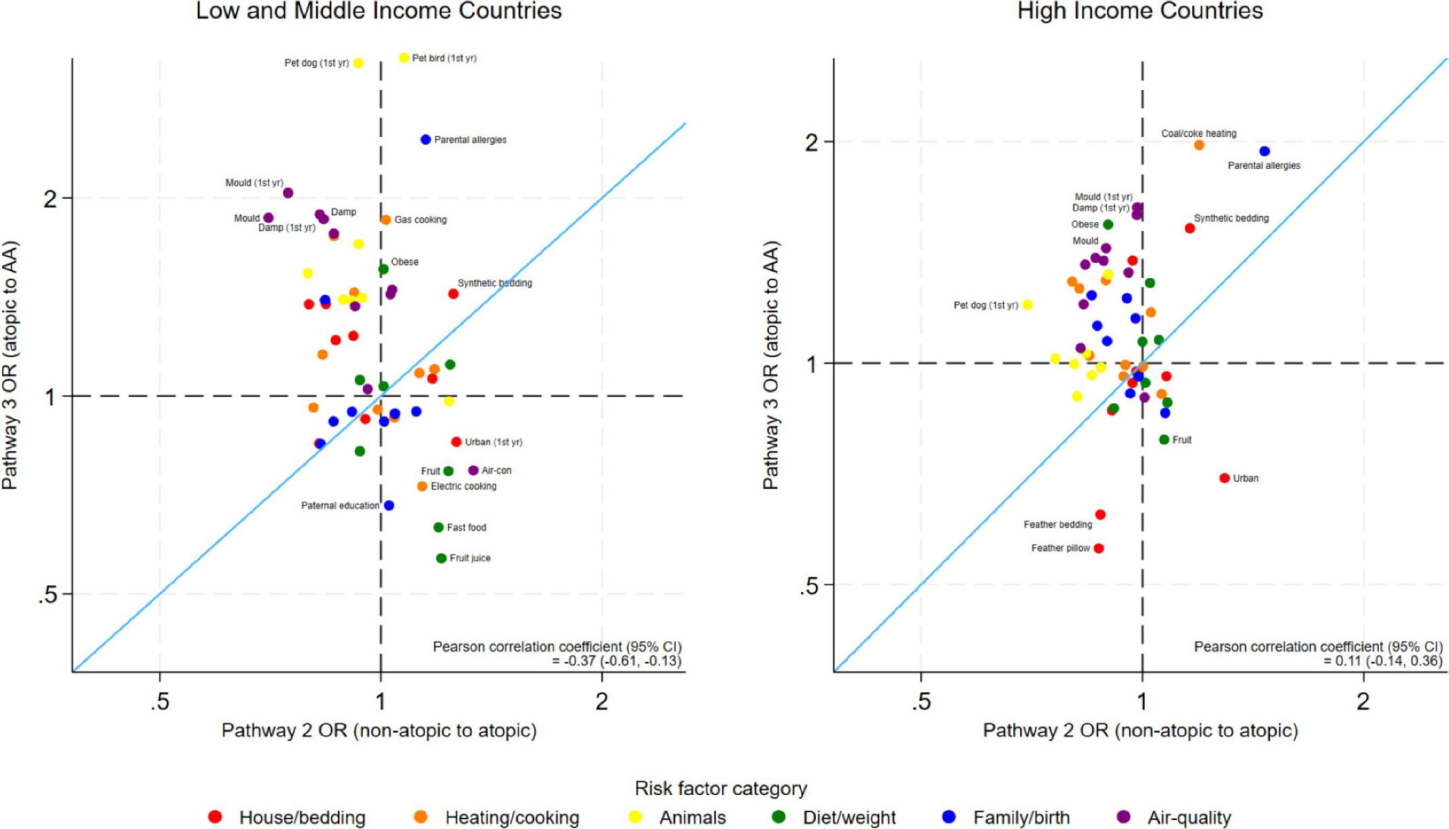
Estimated ORs of risk factors for asthma (in atopic individuals) compared with atopy (pathway 3 vs pathway 2) stratified by country affluence. Axes are shown on the natural log scale. AA, atopic asthma.

Stratified by country affluence, there was still a positive correlation between risk factors for AA and NAA in both settings, but some of the strongest risk factors were quite different by setting (figure 4). The risk factors for atopy were unrelated to the risk factors of asthma in HICs, but in LMICs there was evidence of a negative association (figure 5).

Notably, pet birds and dogs were strong risk factors for asthma in LMICs but not in HICs. Coal/coke heating was a risk factor for AA in HICs but not LMICs. The protective effect of feather bedding and pillows was only found in HICs. Regular fast food was protective for AA (but not NAA) in LMICs only. Detailed results of these stratified models are found in online supplemental table S7.

Results for the separate studies (online supplemental table S8-11) showed some differences, though the WASP study alone was relatively small, resulting in wide CIs.

The sensitivity analyses with different atopy definitions showed similar outcomes. Using only ISAAC and ALSPAC but with the standard atopy definition (n=26 496), the correlation between risk factors for AA in already atopic individuals (pathway 3) and NAA in non-atopic individuals (pathway 1) was lower but still very strongly positive (PCC=0.80, bootstrapped 95% CI 0.67 to 0.93), and the correlation between risk factors for atopy (pathway 2) and AA in atopic individuals (pathway 3) was still very low (−0.12 (−0.33 to 0.09)). Using wheal size ≥5 mm for any core allergen (n=23 188), the correlation between risk factors for AA and NAA was slightly less strong (0.63 (0.45 to 0.81)) and there was no correlation between risk factors for AA and atopy (−0.13 (−0.36 to 0.10)). Using wheal size ≥4 mm for two or more core allergens (n=22 243) resulted in similar findings (0.63 (0.45 to 0.81) and −0.17 (−0.41 to 0.07)) (online supplemental figure S11 and S12 and online supplemental table S12).

The second sensitivity analysis leaving out one study at a time also showed similar findings to the main analyses. The correlation between risk factors for AA and NAA was slightly lower when leaving out the ISAAC data (0.62 (0.36 to 0.88)), but the CI on the correlation coefficient was wider due to the lower sample size (n=9085) (online supplemental figure S13-S16 and online supplemental table S13).

The last sensitivity analysis, allowing for a small amount of missing symptom data, increased the sample size by 3668 (n=36 409) and produced similar findings to the main analyses (online supplemental figure S17 and table S13).

## Discussion

We used a novel approach to assess risk factors for the different pathways to AA and NAA in a large and diverse population representing both HICs and LMICs.

Our findings from the available variables indicate that lifestyle and environmental risk factors are similar for NAA (in non-atopic children) and AA (in atopic children) but not for atopy. For example, dog contact in the first year of life showed a strong association for AA in atopics (OR=1.41), weaker evidence of effect for NAA in non-atopics (1.10) and a protective association with atopy (0.73); frequent fruit intake showed strong and consistent protective associations for asthma (NAA 0.80; AA 0.78) but a positive association with atopy (1.09); maternal smoking at various times in the child’s life was strongly associated with increased risk of asthma (NAA and AA) but reduced risk of atopy. The exception was parental history of allergic diseases, which was a strong risk factor for both forms of asthma (NAA 2.45; AA 2.05) and atopy (1.38).

Although atopy (defined using SPT) is associated with increased risk of asthma, other risk factors are generally the same for AA and NAA. There may be important risk factors that were not included in this study, particularly for NAA which has not been as extensively studied, and the results may have been different if these ‘other’ risk factors (eg, air pollution, viral infections, stress) had been examined. Nevertheless, we have included a large number of commonly recognised and studied risk factors in our analyses, including most of the risk factors that have been identified as being associated with asthma in major studies such as ISAAC.^19 20^ The fact that we have found these risk factors to have similar associations with both asthma in atopics (AA) and asthma in non-atopics (NAA) raises important questions about the role of atopy in asthma, particularly whether it is an inherent part of the aetiological process, or an exacerbating factor (or a mix of both), or is coincidental. Alternatively, NAA and AA could be separate phenotypes caused by different mechanisms for the initial onset of asthma but with similar exacerbating factors. However, this cannot be established from our cross-sectional studies as it is not possible to differentiate risk factors for initial onset from those for exacerbation of pre-existing asthma.

Risk factors in the animal and air quality categories showed a particularly striking pattern of negative associations with atopy but positive associations with asthma (both AA and NAA) (online supplemental figure S7 and S9). This is apparent for both early-life and current risk factors, making it unlikely to be due to reverse causation or specific to exacerbation of symptoms.

In comparing our method of investigating different pathways to the other methods listed in the introduction, we found, as an example, that fruit intake (which was protective for NAA and AA but a risk factor for atopy) was protective when comparing all asthma to all non-asthma, also when adjusting for atopy, and when comparing NAA to all people without asthma. However, this effect was not seen when comparing AA to all people without asthma (as the effect was confounded by atopy). If the differences between risk factors for NAA and AA were greater than we observed, consideration of the separate pathways is even more important.

A strength of this study is the inclusion of data from 23 different countries, including both HICs and LMICs, as well as the resulting large sample size. Previous analyses of ISAAC data have generally found the risk factors for asthma to be similar in HICs and LMICs, providing for triangulation of findings^21^ and increasing the likelihood that the associations are causal. However, there are difficulties in ensuring the relevance of questions in varying settings, for example, heating questions in different climates. In addition, some risk factors may be differentially associated with socioeconomic position, which we partially assessed by stratifying the results by country income level, but we do not have the information to assess differences within countries.

The strongest risk factor for both types of asthma, and for atopy alone, was parental history of allergic disease, consistent with previously published findings.^9 22^ Other strong risk factors for asthma (AA and NAA), but not atopy, were damp and mould in the home, which are already established risk factors for childhood asthma (both aetiology and exacerbation).^9^ Obesity was a risk factor for both AA and NAA in HICs, which is consistent with literature,^9^ but we did not see this in LMICs, whereas pet birds and dogs were risk factors for AA and NAA only in LMICs. These differences could indicate that associations were due to confounding, or that there were other uncaptured differences between settings, for example, bird contact could mean one pet budgie or a brood of chickens.

Other limitations of these analyses should be considered. The associations reported here will likely be subject to residual confounding as not all asthma risk factors are known or were recorded in all the included studies. Furthermore, the questionnaire was relatively simple, and most questions were binary. For some risk factors, reverse causation is a possibility, for example, those with asthma (or family members) may have changed to synthetic bedding if feathers exacerbate symptoms. Selection bias could be a problem with males and younger people being more likely to have missing data, along with a higher chance of missing the SPT for those who never had asthma. Recall bias could also be an issue, with parents of children with asthma more likely to recall information on known risk factors than parents of children without asthma. There is evidence of a disassociation between SPT and IgE in LMICs where some individuals with negative SPT are IgE positive to the same allergen.^23^ In addition, the European Academy of Allergy and Clinical Immunology, in 2023, proposed a broader classification of allergy, including hypersensitivity reactions far beyond IgE-mediated phenomena (atopy).^24^ Both these factors could indicate a higher proportion of asthma cases are in fact allergic (using the above definition) than that which we find here using SPT alone.

Our findings are important as they point towards similar mechanisms in AA and NAA, once the risk factors for atopy itself are removed. With SPT positivity defining atopy, there is no evidence that the risk factors differ between asthma phenotypes, even after using stricter definitions in sensitivity analyses. This suggests that atopy and asthma may be coincidental in a proportion of children who are defined as having AA. To confirm or refute these findings, further work on identifying different phenotypes of asthma should be undertaken in a variety of populations, including using other markers and data sources (eg, microbiome and epigenetics). IgE analysis could identify patterns in potential allergens, and clinical tests could investigate the role of neurological triggers.

## Supplementary material

10.1136/thorax-2024-222118 online supplemental file 1

10.1136/thorax-2024-222118 online supplemental file 2

## Data Availability

Data are available in a public, open access repository. Data are available upon reasonable request. Data may be obtained from a third party and are not publicly available.
