# Supplementary material for: Risk factors for atopic and non-atopic asthma in school-age children from high-income and low- and middle-income countries
Source: Thorax. 2025 Jun 24;80(12):e222118. doi: 10.1136/thorax-2024-222118 (PMC12703255; doi:10.1136/thorax-2024-222118)
Supplement: online supplemental file 1 [file thorax-80-12-s001.pdf]

Risk factors for atopic and non-atopic asthma in school-aged children from high-, and low-and-middle-income countries: Supplementary material

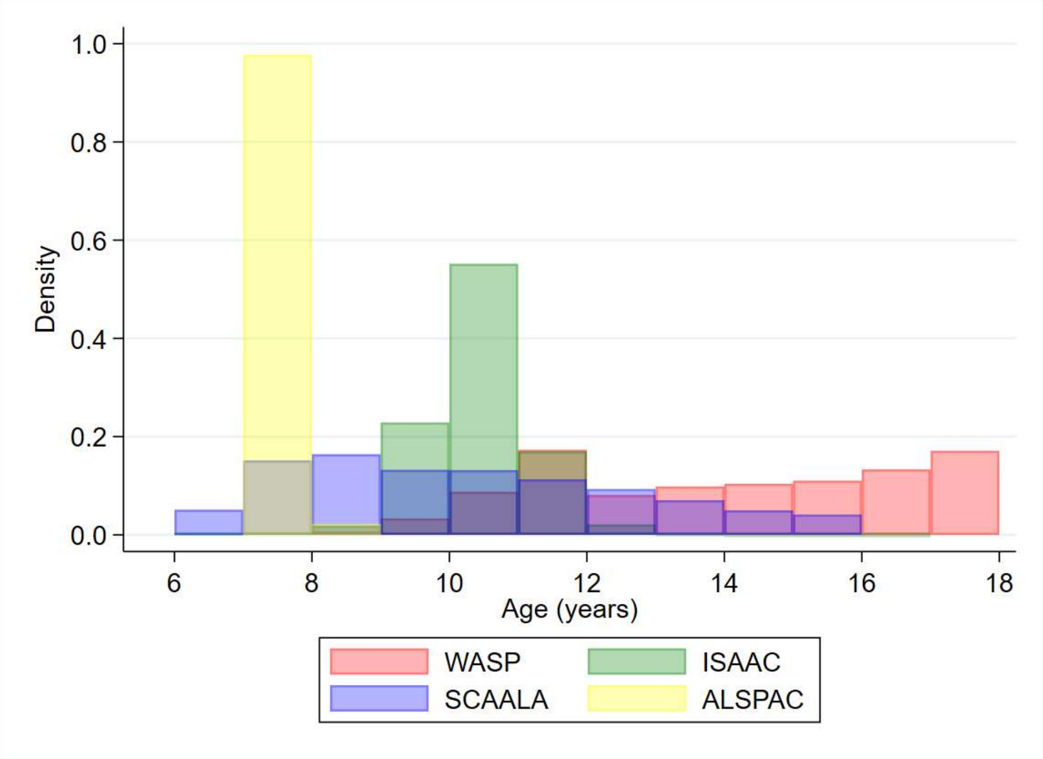

Figure S1: Age distribution by study

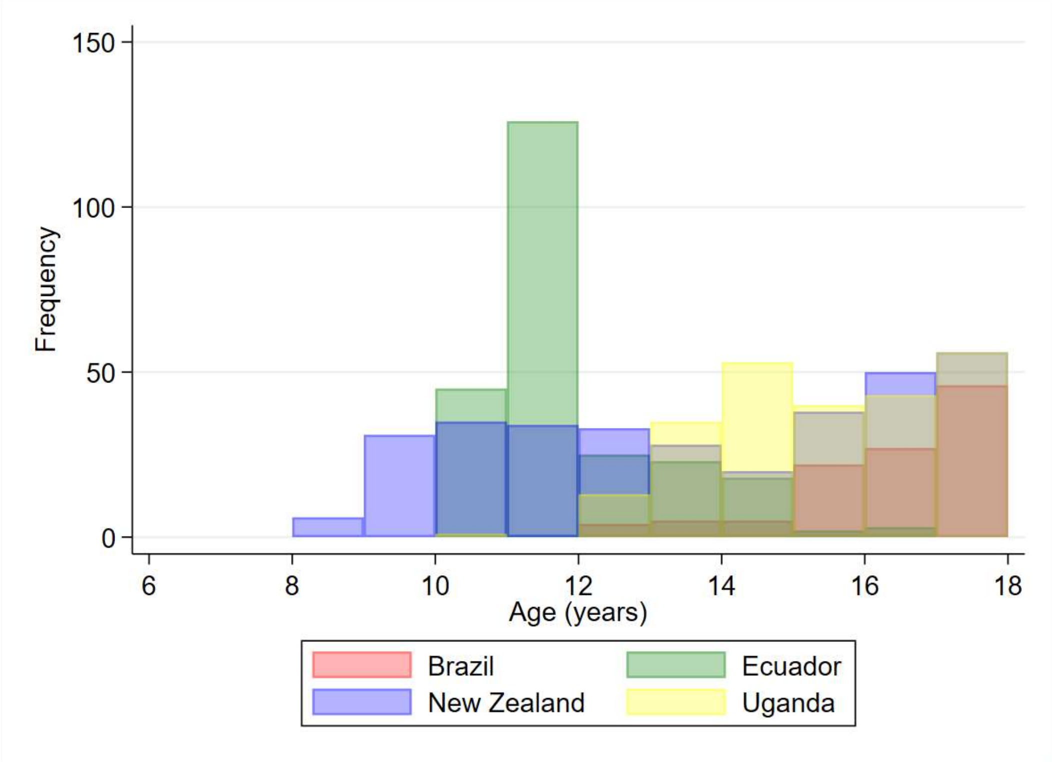

Figure S2: Age distribution within World Asthma Phenotypes (WASP) study

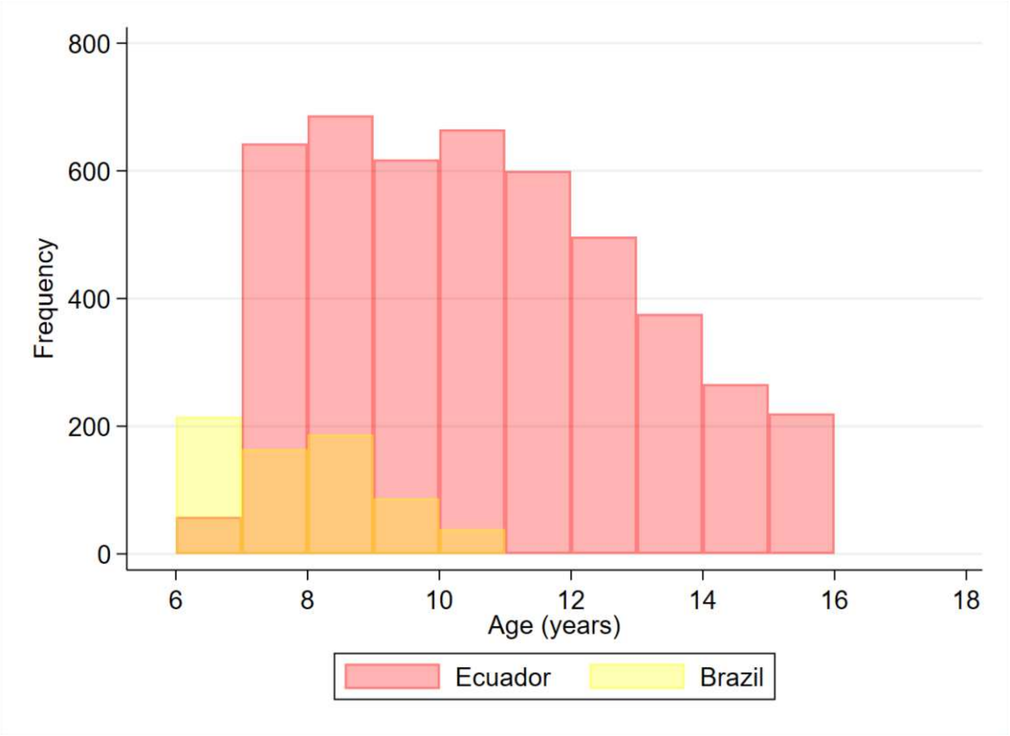

Figure S3: Age distribution within Social Changes Asthma and Allergy in Latin America (SCAALA) study

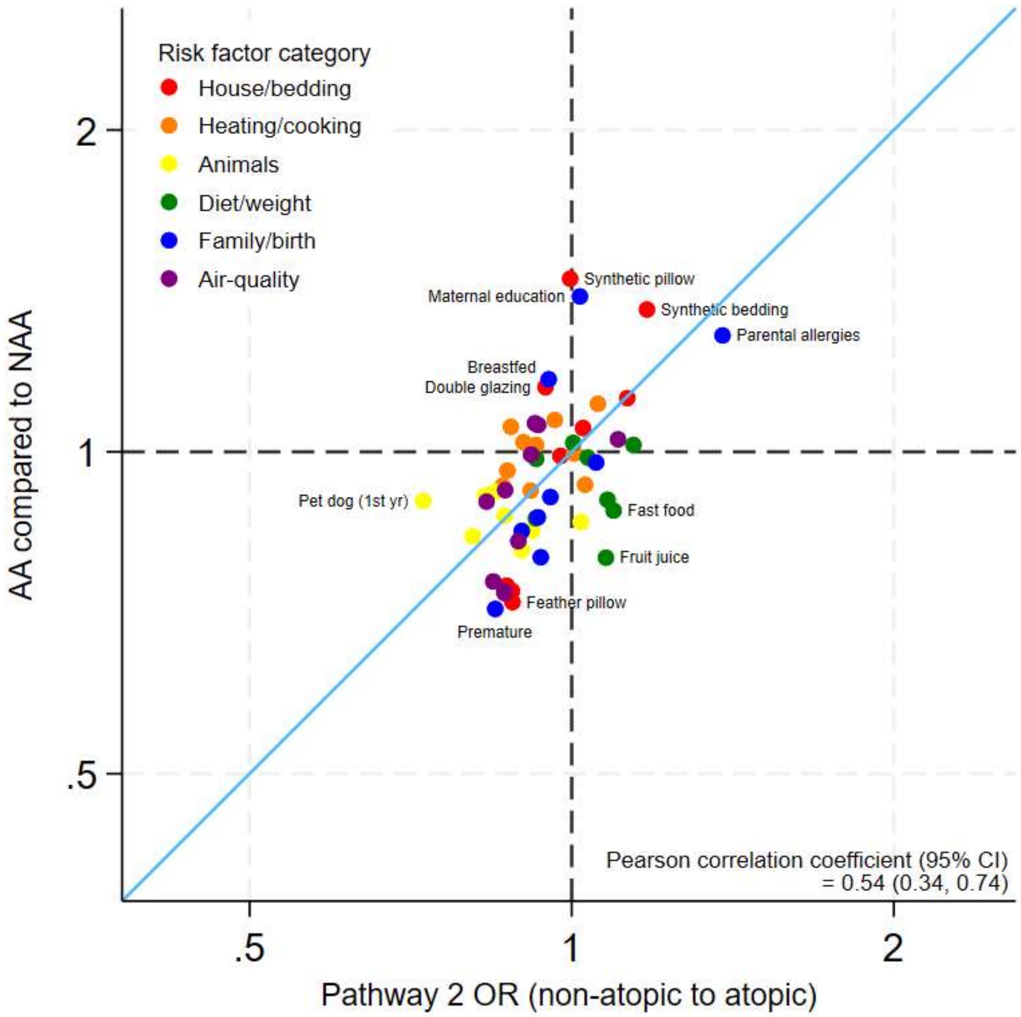

Figure S4: Comparison of risk factors for atopy to difference between atopic asthma (AA) and non-atopic asthma (NAA)

Axes are shown on the natural log scale.

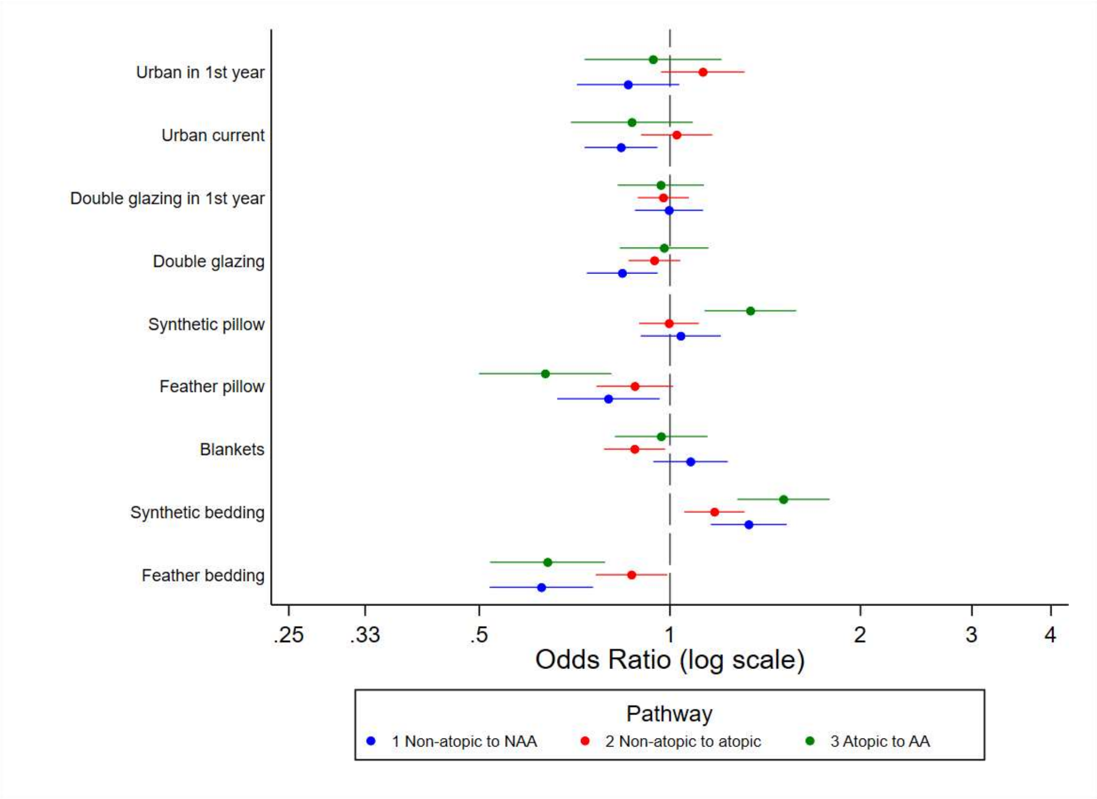

Figure S5: Comparing pathways to asthma for house / bedding related risk factors

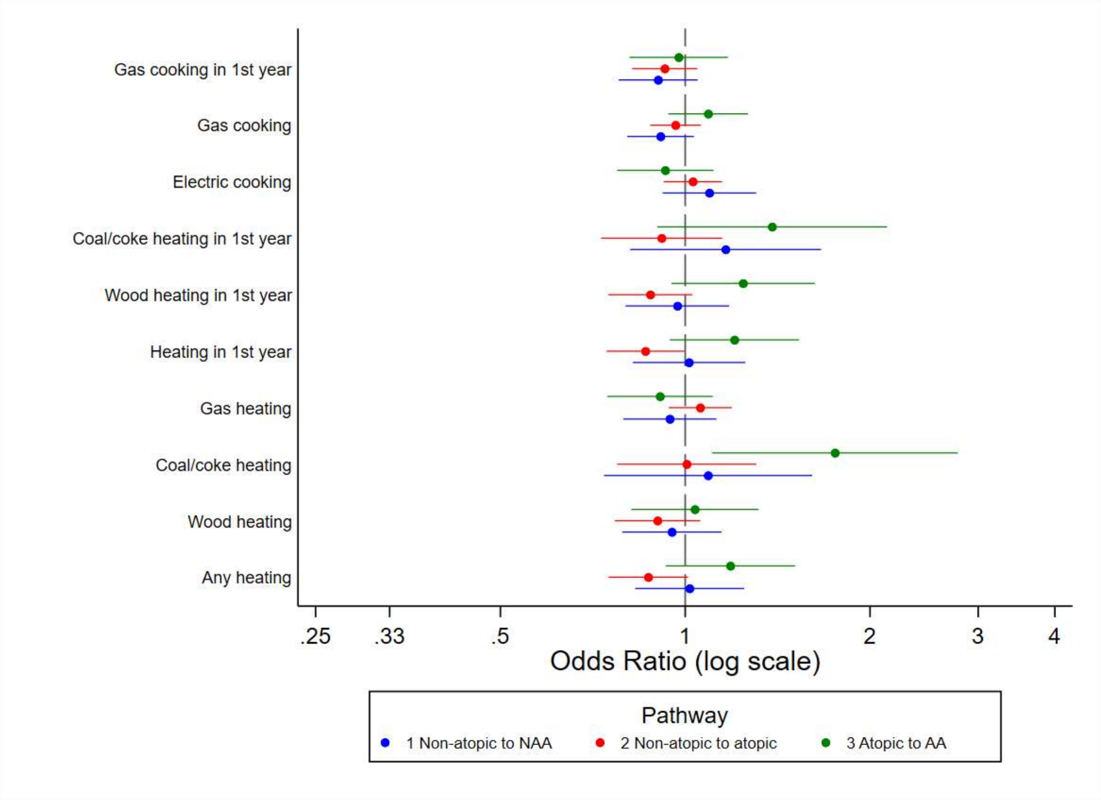

Figure S6: Comparing pathways to asthma for heating / cooking risk factors

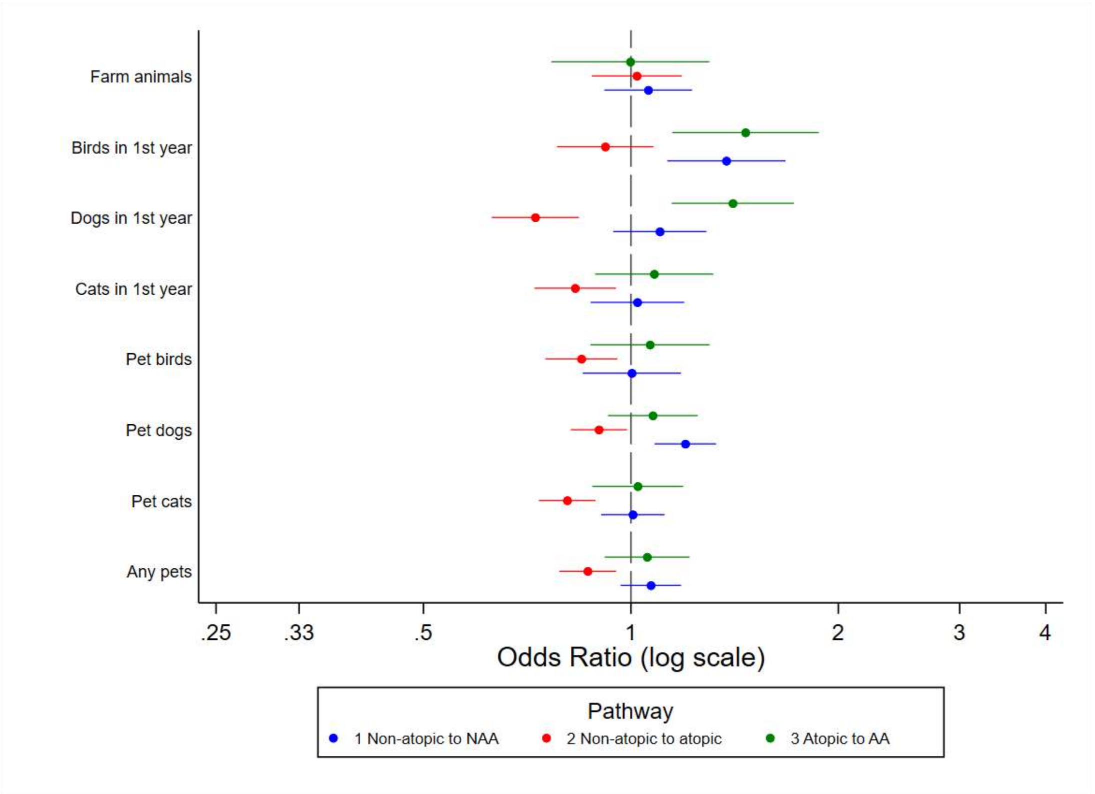

Figure S7: Comparing pathways to asthma for animal risk factors

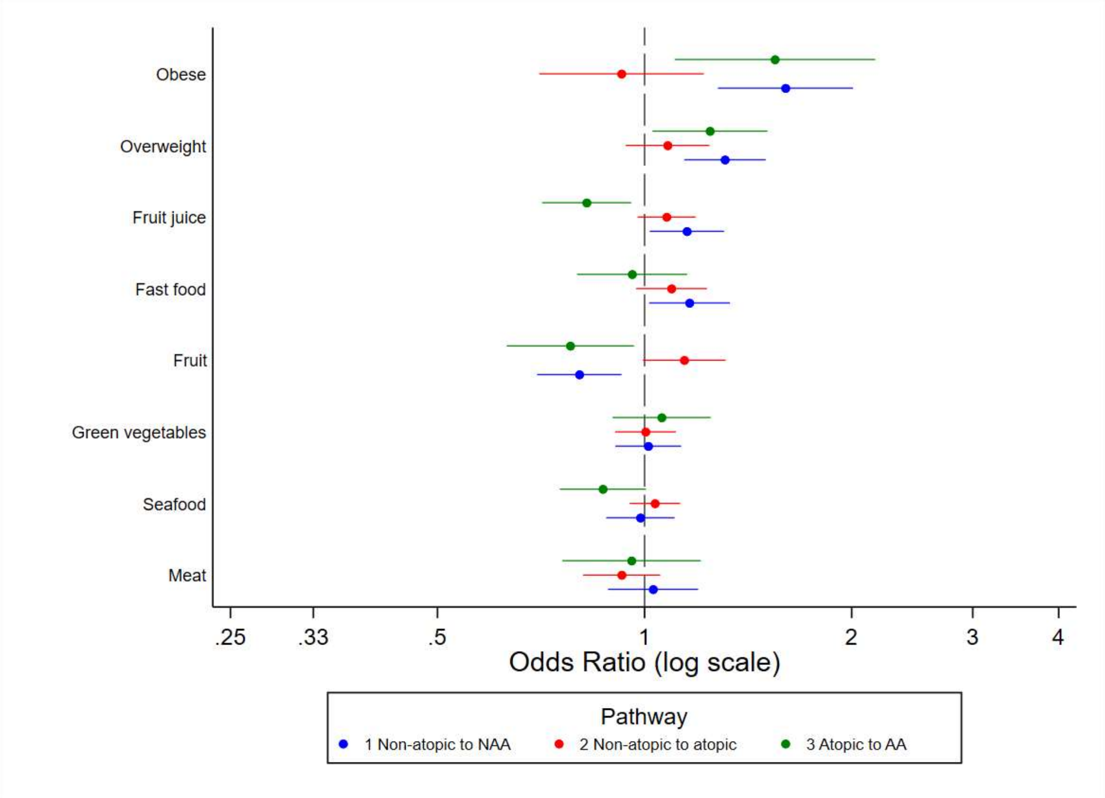

Figure S8: Comparing pathways to asthma for dietary / weight related risk factors

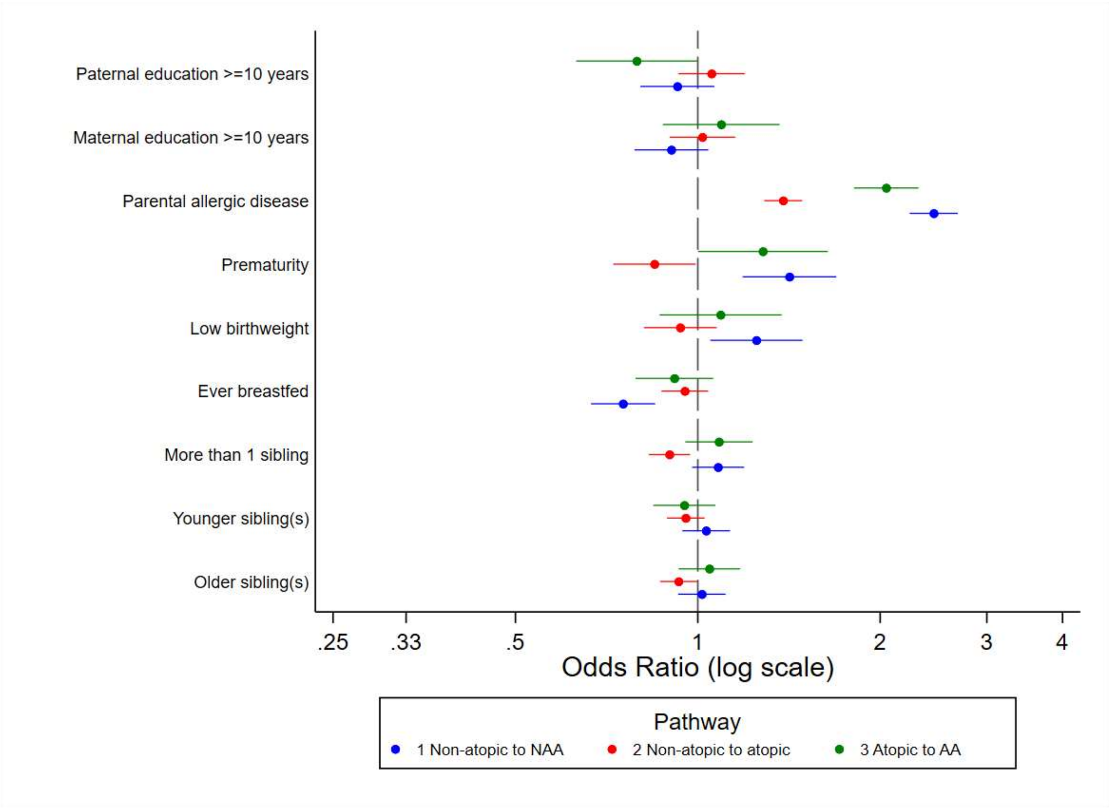

Figure S9: Comparing pathways to asthma for family / birth related risk factors

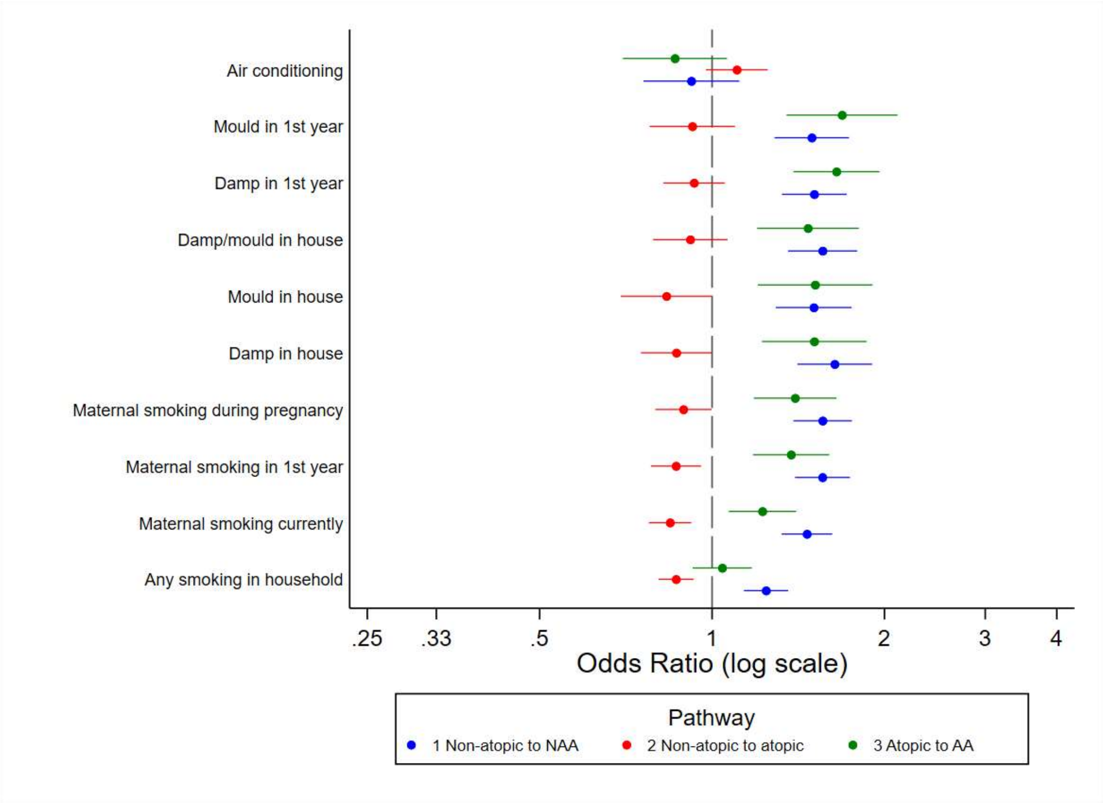

Figure S10: Comparing pathways to asthma for air-quality related risk factors

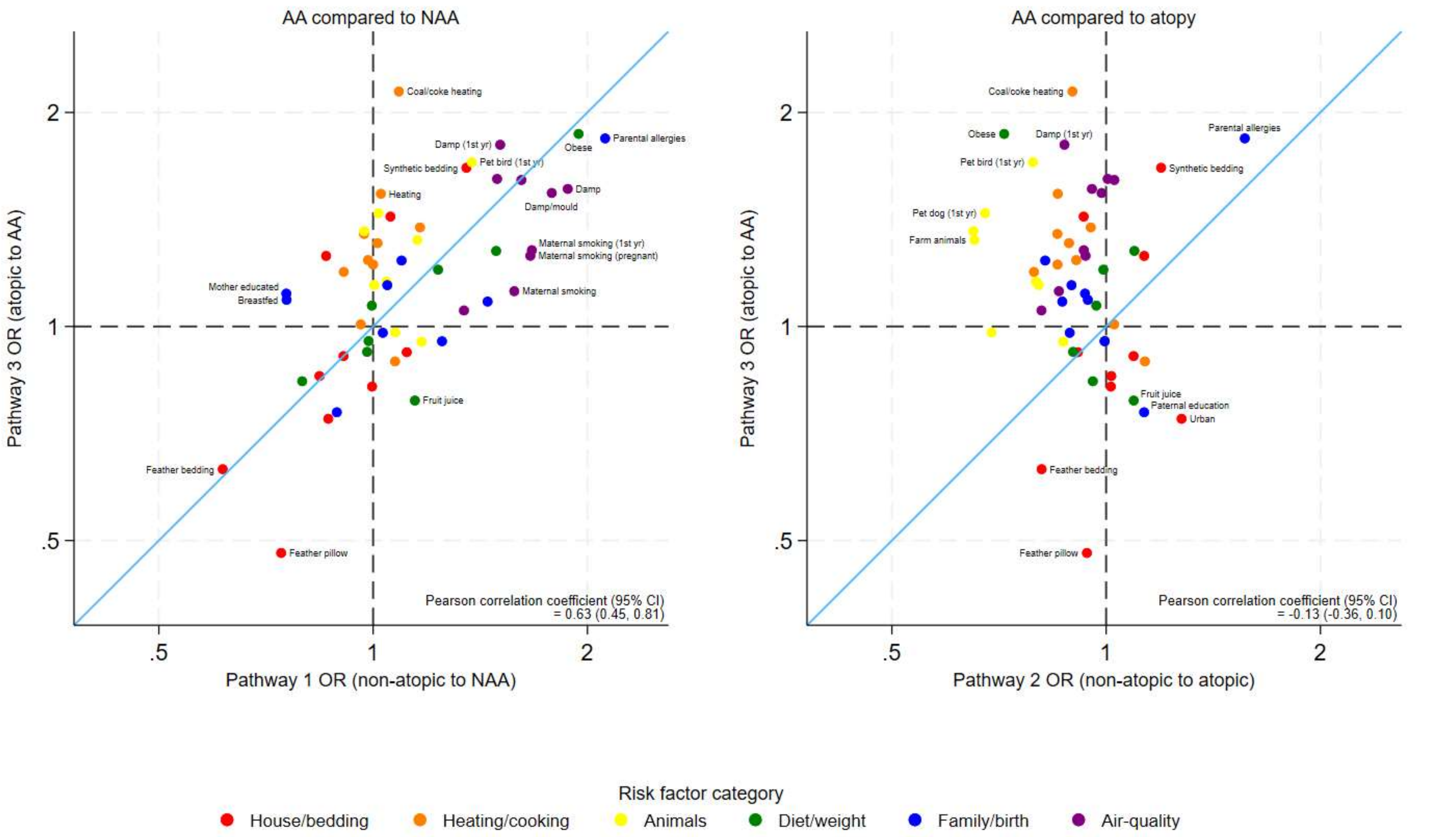

Figure S11: Sensitivity analysis results using any wheal >=5mm in core allergens

Axes are shown on the natural log scale. Only includes ALSPAC and ISAAC data.

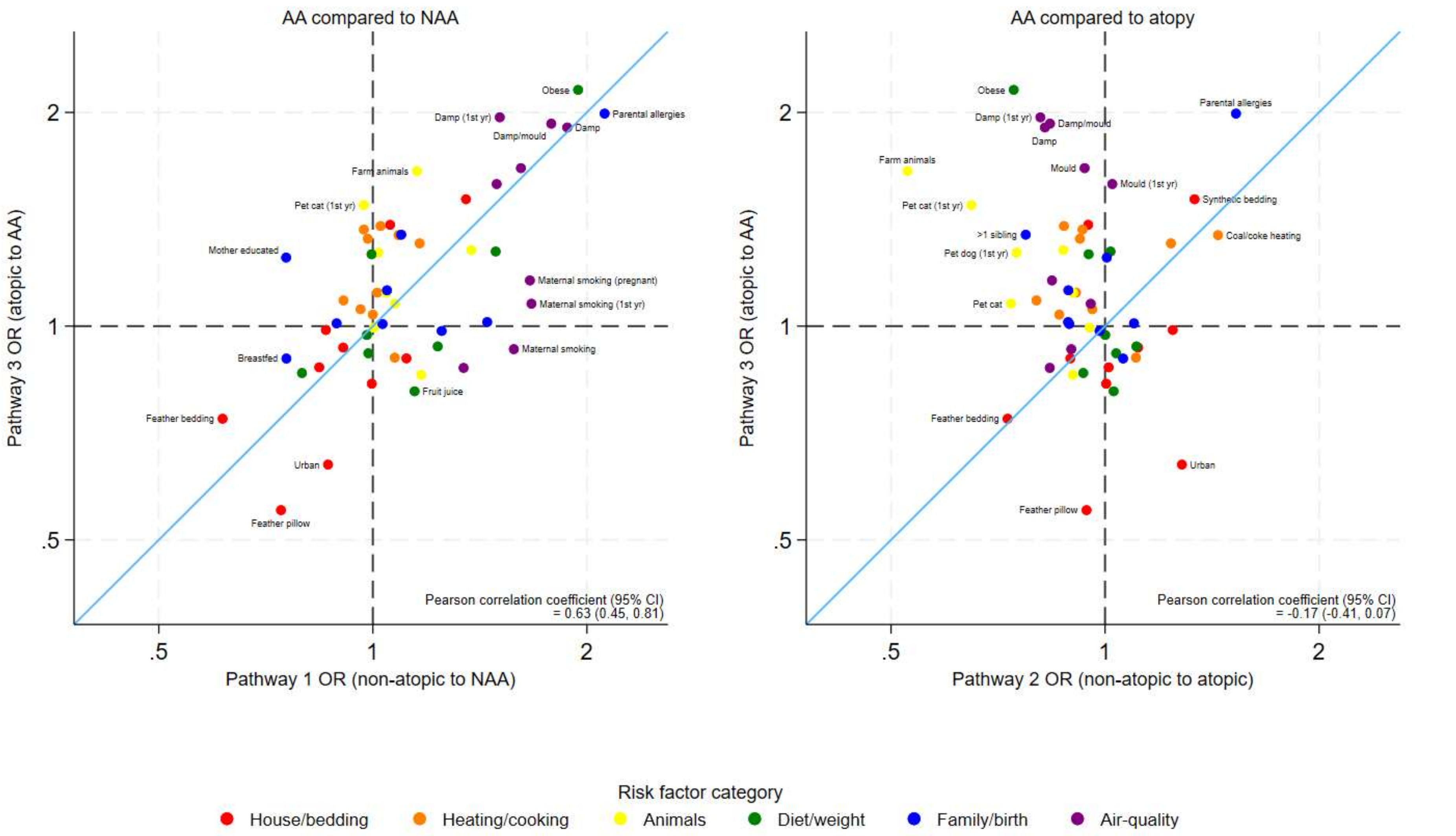

Figure S12: Sensitivity analysis results using wheal >=4mm for 2+ core allergens

Axes are shown on the natural log scale. Only includes ALSPAC and ISAAC data.

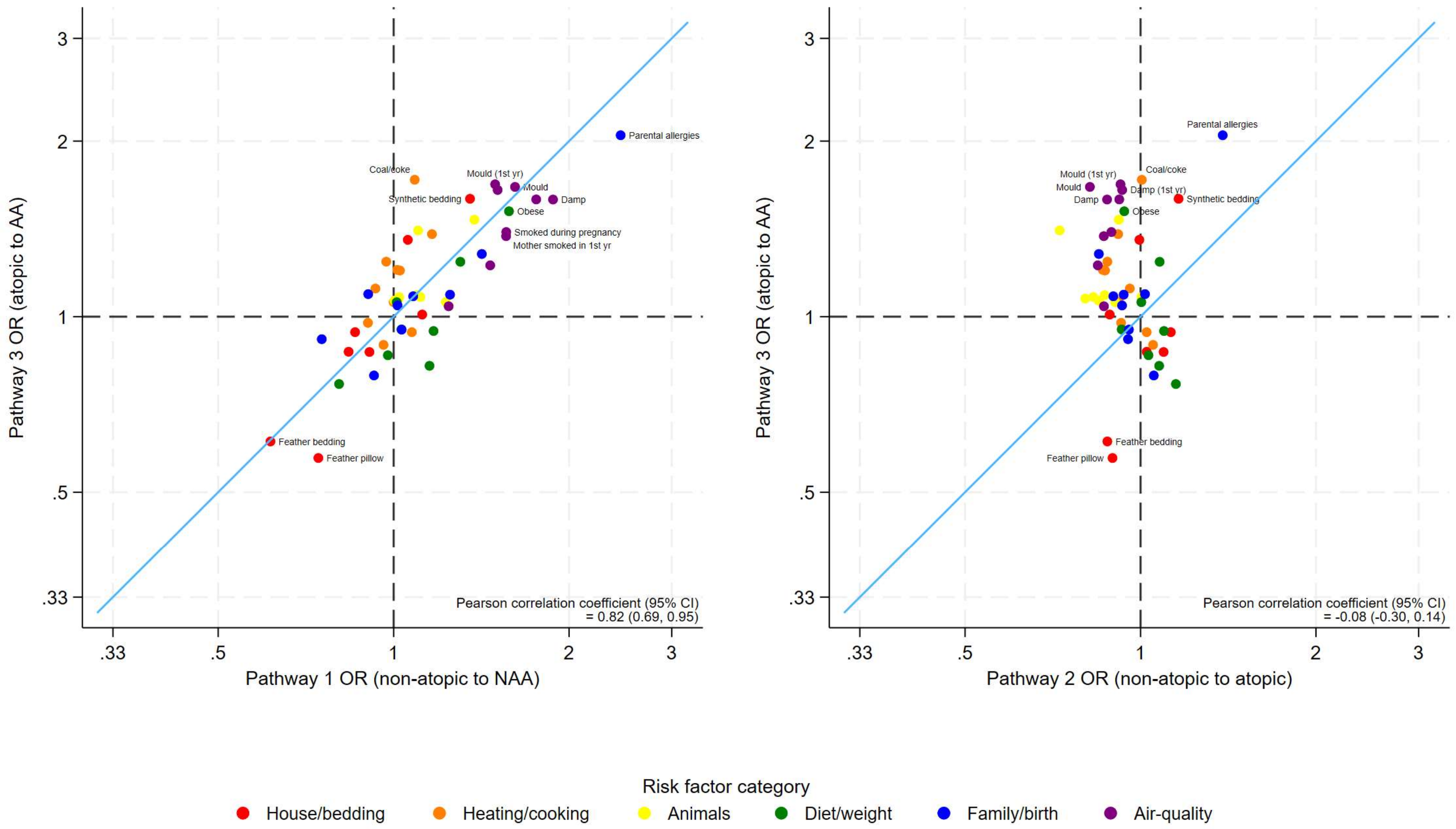

Figure S13: Sensitivity analysis leaving out the WASP study data

Axes are shown on the natural log scale.

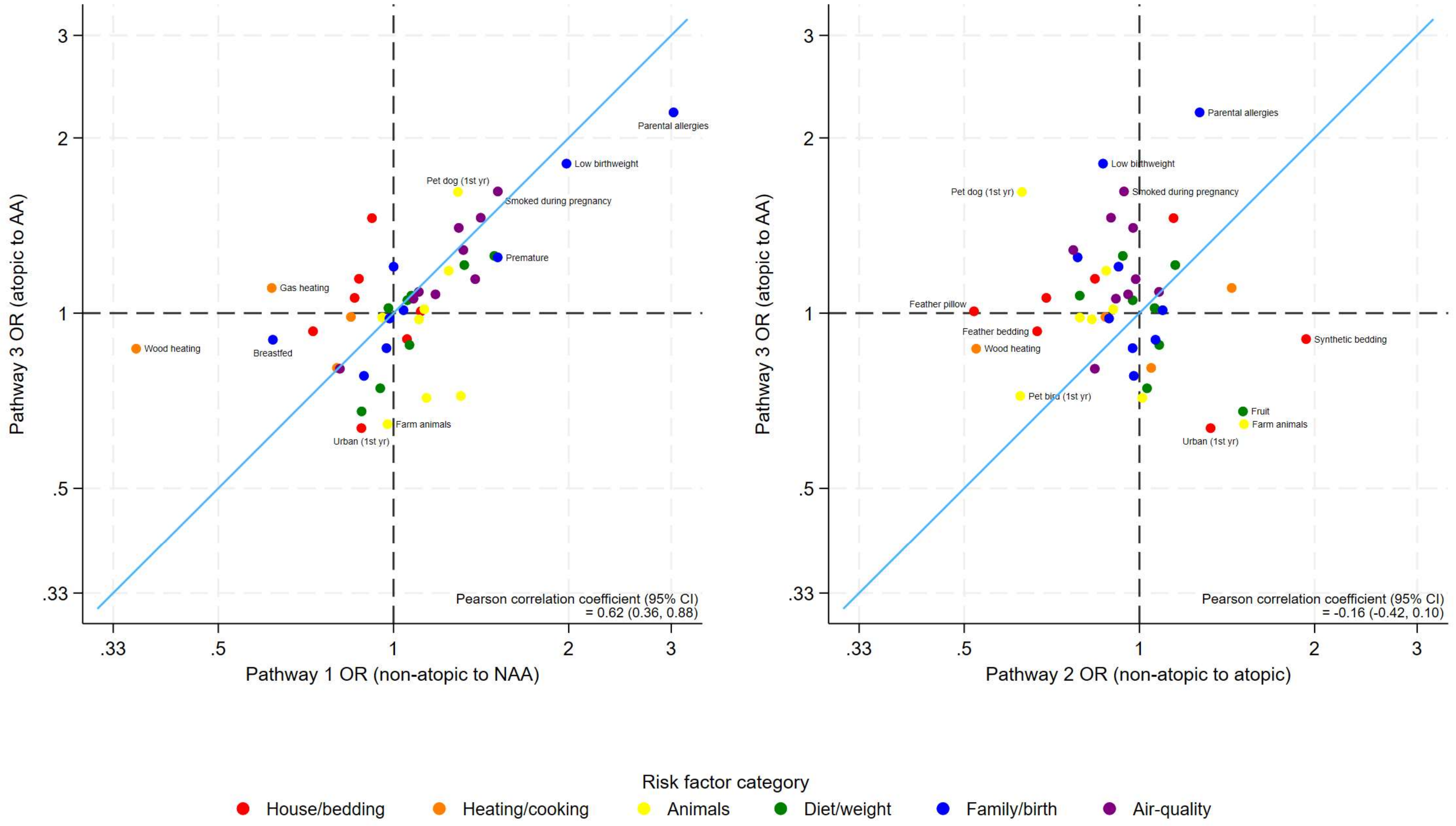

Figure S14: Sensitivity analysis leaving out the ISAAC study data

Axes are shown on the natural log scale.

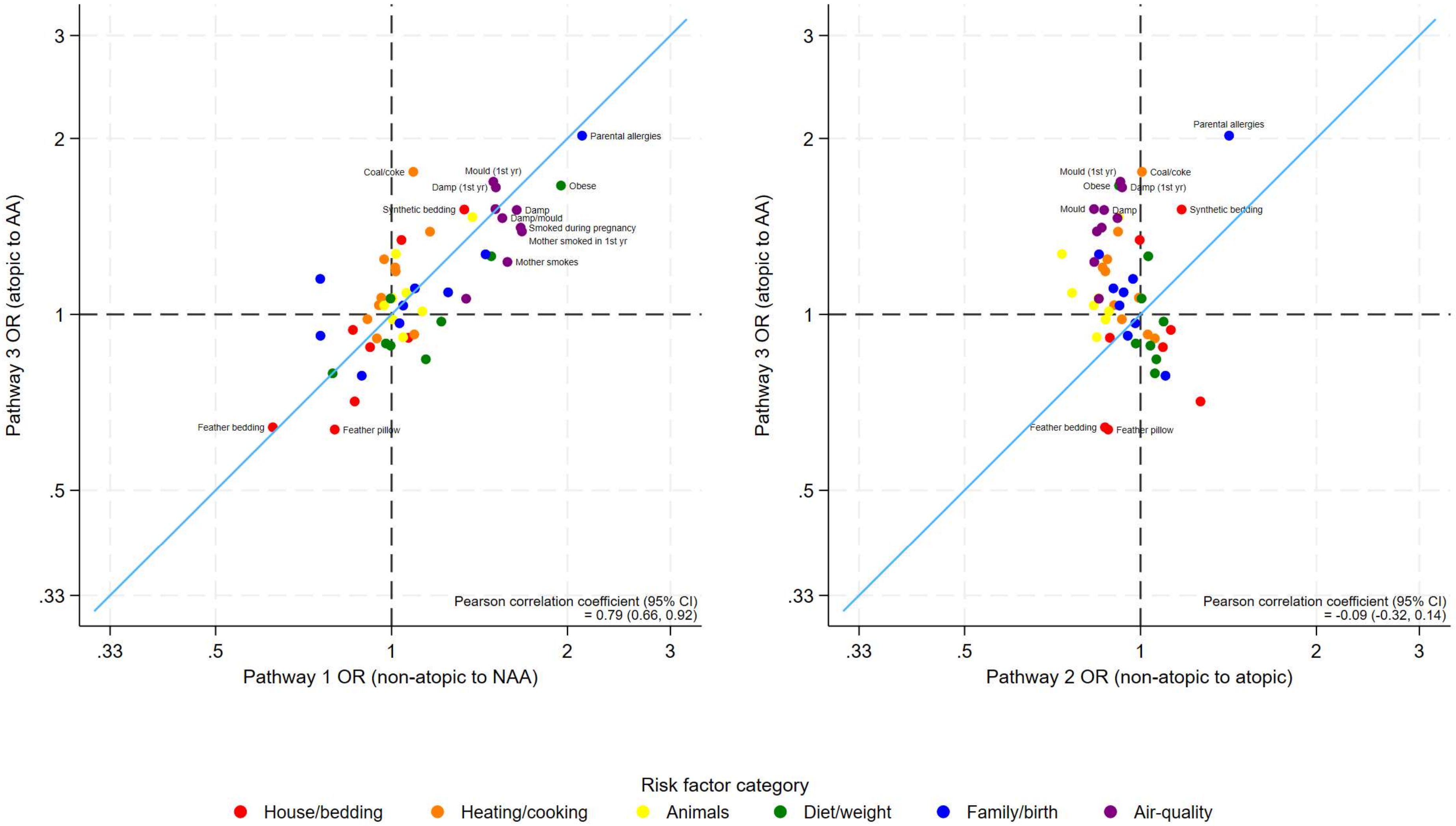

Figure S15: Sensitivity analysis leaving out the SCAALA study data

Axes are shown on the natural log scale.

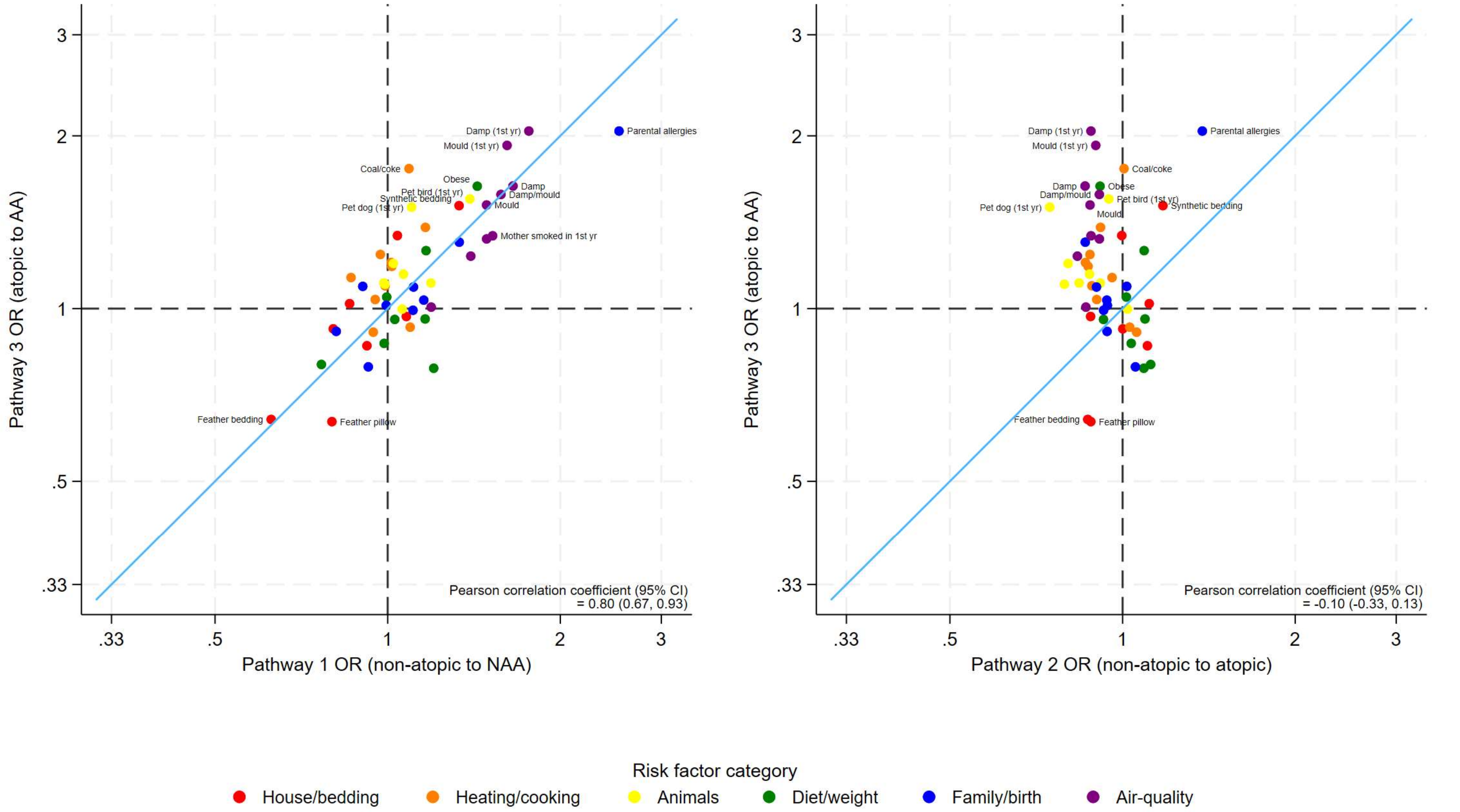

Figure S16: Sensitivity analysis leaving out the ALSPAC study data

Axes are shown on the natural log scale.

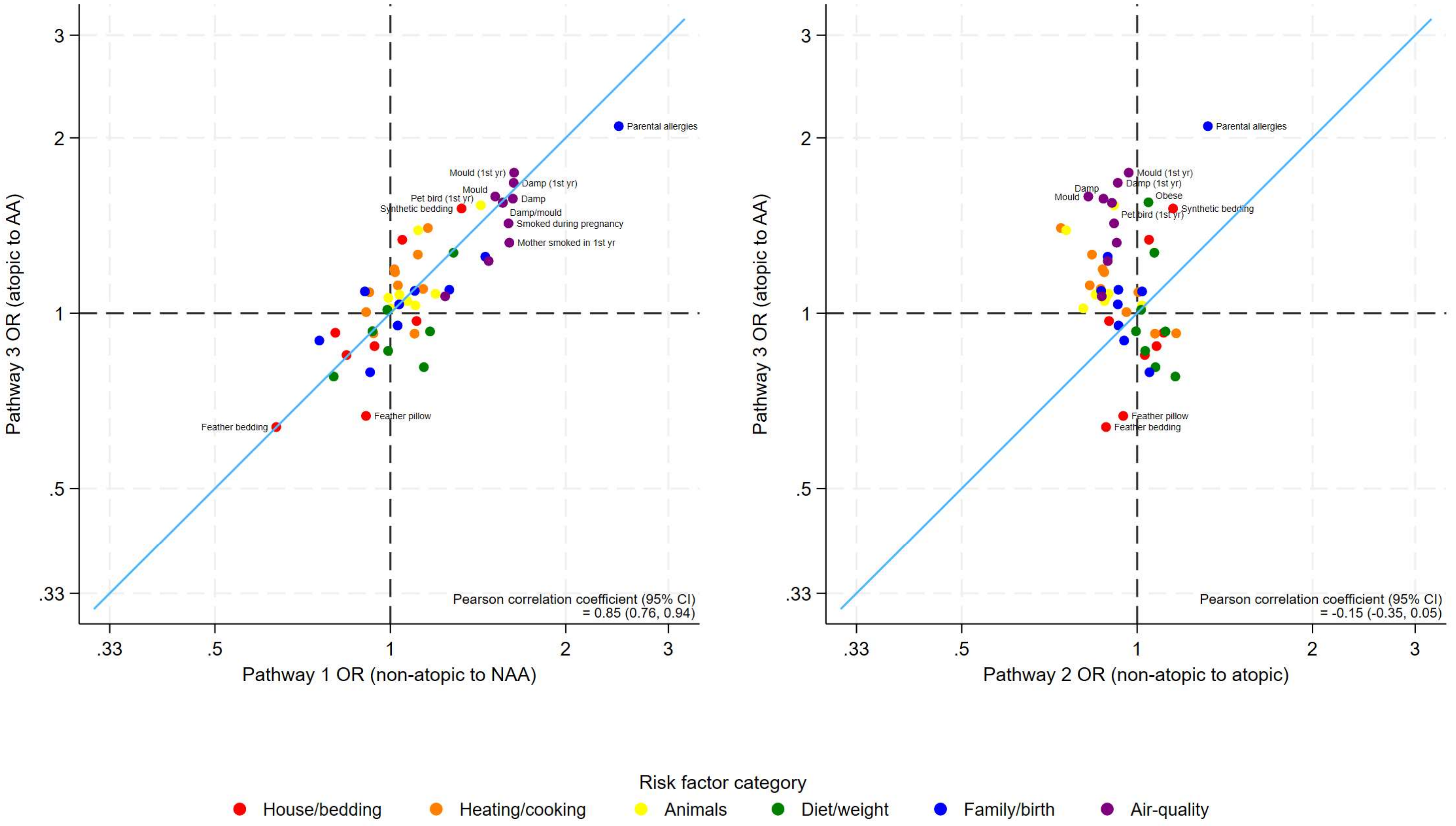

Figure S17: Sensitivity analysis using lenient control definition (allowing some missing data)

Axes are shown on the natural log scale.

Table S1: Comparison of questions used to define variables across studies

Study:	WASP		ISAAC		SCAALA		ALSPAC[a]	
Variable	Question	Answer	Question	Answer			Question	Answer
Current asthma	1) Has your child ever had wheeze (a whistling sound from the chest) at any time in the past?<br>2) Has he or she had wheezing or whistling in his or her chest at any time in the past 12 months?<br>In the past 12 months, has he or she taken any medication, pills, inhalers, or other medications for asthma or wheeze?	1) yes<br>and<br>either<br>2) yes or 3) yes	1) Has your child ever had wheezing or whistling in the chest at any time in the past?<br>2) Has your child had wheezing or whistling in the chest in the last 12 months?<br>3) In the past 12 months, has your child used any medicines, pills, puffers or other medication for wheezing or asthma?	1) yes or missing<br>and<br>either<br>2) yes or 3) yes	1) Has your child ever had wheezing or whistling in the chest at any time in the past?<br>2) Has your child had wheezing or whistling in the chest in the last 12 months?<br>3) In the past 12 months, has your child used any medicines, pills, puffers or other medication for wheezing or asthma?	1) yes or missing<br>and<br>either<br>2) yes or 3) yes	1) Has your child had any wheezing in the past 12 months?<br>2) Has your child had asthma medication in the past 12 months?	1) Yes @7y 7m<br>or<br>2) Yes @7y 7m
Never asthma	As above plus:<br>4) Has he or she ever had asthma?	1) no and<br>2) no and<br>3) no and<br>4) no	As above plus:<br>4) Has your child ever had asthma?	1) no and<br>2) no and<br>3) no or missing<br>and<br>4) no	As above plus:<br>4) Has your child ever had asthma?	1) no and<br>2) no and<br>3) no or missing<br>and<br>4) no or missing	1) Has your child had any wheezing in the past X months [since last survey]?<br>2) Has your child had asthma in the past 12 months?<br>3) In the past year has your child had any periods when there was wheezing with whistling on their chest when they breathed?<br>4) Has a doctor ever actually said that your child has asthma?	1) No @7y 7m & @6y 9m & @5y 9m & @4y 9m & @3y 6m & @2y 6m & @1y 6m & @6m<br>and<br>2) no @7y 7m & @ 6y 9m<br>and<br>3) no @ 6y 9m<br>and<br>4) no @7y 7m
Atopy	Skin prick test[b]	>= 1 reaction to allergens, correct +ve and -ve controls and >=5 allergens tested	Skin prick test[b]	>= 1 reaction to allergens, correct +ve and -ve controls<br>and >=5 allergens tested	Skin prick test[b]	>= 1 reaction to allergens, correct +ve and -ve controls<br>and >=5 allergens tested	Skin prick test[b] from clinic at age 7y 6m	>= 1 reaction to allergens, correct +ve and -ve controls<br>and >=5 allergens tested
Non-atopic	Skin prick test[b]	No reaction to any	Skin prick test[b]	No reaction to any allergens,	Skin prick test[b]	No reaction to any allergens,	Skin prick test[b] from clinic at age 7y 6m	No reaction to any allergens,

		allergens, correct +ve and -ve controls and >=5 allergens tested		correct +ve and -ve controls and >=5 allergens tested		correct +ve and -ve controls and >=5 allergens tested		correct +ve and -ve controls and >=5 allergens tested
House / bedding								
Feather bedding	Does your child use a feather/ down duvet or quilt on *his or her* bed?	Yes/No	What kind of bedding does or did your child use? Feather quilt at present?	Yes/No				
Synthetic bedding	Does your child use a synthetic duvet or quilt on *his or her* bed?	Yes/No	What kind of bedding does or did your child use? Synthetic quilt at present?	Yes/No				
Blankets	Does your child use blankets on *his or her* bed?	Yes/No	What kind of bedding does or did your child use? Blankets at present?	Yes/No	Does your child use blankets at present?	Yes/No		
Feather pillow	Does your child use a feather/down pillow on *his or her* bed?	Yes/No	What kind of pillow does or did your child use? Feather pillow at present?	Yes/No				
Synthetic pillow	Does your child use a synthetic pillow on *his or her* bed?	Yes/No	What kind of bedding does or did your child use? Synthetic pillow at present?	Yes/No				
Double glazing			What kind of windows are there in your child's bedroom?	Yes = Yes to sealed unit/double glazing or secondary window<br>No = no to both			Are any of your windows double glazed? (including secondary double glazing)	Yes = All of them, OR Some of them<br>No = None of them
Double glazing in 1st year			What kind of windows were there in your child's bedroom?	Yes = Yes to sealed unit/double glazing or secondary window<br>No = no to both			Are any of your windows double glazed? (including secondary double glazing)	Yes = All of them, OR Some of them<br>No = None of them
Urban			How would you describe the surroundings of your child's home at present?	Urban = Suburban, with many parks or gardens OR Suburban, with few parks or gardens OR Urban			Postcode matched to 2001 census urban/rural indicator	Urban = "urban (pop>10k)" OR "town and fringe"<br>Rural = "village" OR "hamlet and isolated dwelling"

				with no parks or gardens<br>Rural = Rural, open spaces or fields nearby				
Urban in first year			How would you describe the surroundings of your child's home during the child's first year of life?	Urban = Suburban, with many parks or gardens OR Suburban, with few parks or gardens OR Urban with no parks or gardens<br>Rural = Rural, open spaces or fields nearby			Postcode matched to 2001 census urban/rural indicator	Urban = "urban (pop>10k)" OR "town and fringe"<br>Rural = "village" OR "hamlet and isolated dwelling"
Heating / cooking								
Heating	In the past 12 months have you used home heating?	Yes/No	Is your child's home heated at present?	Yes (one or more fire, stove or boiler inside or outside the home)/<br>No (not heated)				
Heating in the first year			Was your child's home heated during the child's first year of life?	Yes (one or more fire, stove or boiler inside or outside the home)/<br>No (not heated)				
Wood for heating	In the past 12 months have you used wood for heating?	Yes/No	Which fuel do you use for heating?<br>Wood (at present)?	Yes/No				
Wood for heating in the first year			Which fuel did you use for heating?<br>Wood (during the child's first year of life)?	Yes/No				
Coal or coke for heating	In the past 12 months have you used coal or coke fire?	Yes/No	Which fuel did you use for heating?<br>Coal or coke (at present)?	Yes/No				
Coal or coke for heating in the first year			Which fuel did you use for heating?<br>Coal or coke (during the child's first year of life)?	Yes/No				

Gas for heating	In the past 12 months have you used any gas heating in your home (or homes)?	Yes/No	Is gas used for heating at present?	Yes/No				
Electric cooking	Is electricity usually used for cooking in your house?	Yes/No	Do you use electricity for cooking at present?	Yes/No	Do you use electricity for cooking at present?	Yes/No		
Gas cooking	In past 12 months have you used gas for any cooking in your home? (not gas BBQ)	Yes/No	Which fuel do you use for cooking?<br>Gas (at present)?	Yes/No	Do you use gas for cooking at present?	Yes/No	Do you use gas for cooking?	Yes = "Yes, ring(s) only" OR "yes, oven only" OR "yes, rings and oven"<br>No = "no, not at all"
Gas cooking in first year			Which fuel did you use for cooking?<br>Gas (during the child's first year of life)?	Yes/No	Did you use gas for cooking in your child's first year of life?	Yes/No	Do you use gas for cooking?	Yes = "Yes, ring(s) only" OR "yes, oven only" OR "yes, rings and oven"<br>No = "no, not at all"
Animals								
Any pets	Are there any pets present in the household?	Yes/No	Which of the following pets do you keep inside your child's home? Do you keep a Dog / Cat / Other furry pet / Bird / Other pet inside your child's home (at present)?	Yes=Yes to one of Dog / Cat / Other furry pet / Bird / Other pet<br>No= No to Dog & Cat & Other furry pet & Bird & Other pet	Derived from 6 questions. Do you keep a dog / cat / chicken / pigeon / parrot / other pet inside your home?	Yes if yes to any / No if no to all	Do you have any pets?	Yes/No
Cats	Cat (listed)	Yes/No	Which of the following pets do you keep inside your child's home? Cats (at present)?	Yes/No	Do you keep a cat inside your home?	Yes/No	How many of the following pets do you have? Cats	Yes = 1+<br>No = 0 OR No to any pets
Cats in first year			Which of the following pets did you keep inside your child's home? Cats (during the child's first year of life)?	Yes/No	Did you keep a cat inside your home during your child's 1st year of life?	Yes/No	How many of the following pets do you have? Cats	Yes = 1+<br>No = 0 OR No to any pets in first year
Dogs	Dog (listed)	Yes/No	Which of the following pets do you keep inside your child's home? Dogs (at present)?	Yes/No	Do you keep a dog inside your home?	Yes/No	How many of the following pets do you have? Dogs	Yes = 1+<br>No = 0 OR No to any pets
Dogs in first year			Which of the following pets did you keep inside your child's home? Dogs (during the child's first year of life)?	Yes/No	Did you keep a dog inside your home during your child's 1st year of life?	Yes/No	How many of the following pets do you have? Dogs	Yes = 1+<br>No = 0 OR No to any pets in first year

Birds			Which of the following pets do you keep inside your child's home? Birds (at present)?	Yes/No			How many of the following pets do you have? Birds (budgerigar, parrot, etc.)	Yes = 1+<br>No = 0 OR No to any pets
Birds in first year			Which of the following pets did you keep inside your child's home? Birds (during the child's first year of life)?	Yes/No			How many of the following pets do you have? Birds (budgerigar, parrot, etc.)	Yes = 1+<br>No = 0 OR No to any pets in first year
Farm animal contact	Does your child currently have regular contact with cattle, sheep, horses, pigs, poultry, goats or other farm animals?	Yes/No derived from separate yes/no questions on each animal	Does your child have at least once a week contact with farm animals outside your child's home?	Yes/No	Does your child have any contact with farm animals? (Ecuador only)	Yes/No		
Diet / weight								
Meat	In the past 12 months, how often on average, did you eat meat (e.g. beef, lamb, chicken, pork)	Less than once per week = "Never or only occasionally"<br>At least once a week = "Once or twice per week" OR "Most or all days"	How often, on average, does your child eat meat nowadays?	Less than once per week = "Never" OR "Less than once per week"<br>At least once a week = "1-2 times per week" OR "3-6 times per week" OR "Once per day or more often"	How often, on average, does your child eat meat, nowadays?	Less than once per week = "Never" OR "Less than once per week"<br>At least once a week = "1-2 times per week" OR "3-6 times per week" OR "Once per day or more often"		
Fish	In the past 12 months, how often on average, did you eat seafood (including fish)	Less than once per week = "Never or only occasionally"<br>At least once a week = "Once or twice per week" OR	How often, on average, does your child eat fish nowadays?	Less than once per week = "Never" OR "Less than once per week"<br>At least once a week = "1-2 times per week" OR "3-6 times per week" OR "Once per day or more often"	How often, on average, does your child eat fish, nowadays?	Less than once per week = "Never" OR "Less than once per week"<br>At least once a week = "1-2 times per week" OR "3-6 times per week" OR "Once per day or more often"		

		"Most or all days"						
Fruit	In the past 12 months, how often on average, did you eat fruit	Less than once per week = "Never or only occasionally" At least once a week = "Once or twice per week" OR "Most or all days"	How often, on average, does your child eat fresh fruit nowadays?	Less than once per week = "Never" OR "Less than once per week" At least once a week = "1-2 times per week" OR "3-6 times per week" OR "Once per day or more often"	How often, on average, does your child eat fresh fruit, nowadays?	Less than once per week = "Never" OR "Less than once per week" At least once a week = "1-2 times per week" OR "3-6 times per week" OR "Once per day or more often"	Thinking about all the food **that you provide** (**Do not include meals provided by school).** How often does your child eat 1. Fresh citrus fruit (e.g. oranges, grapefruit , satsumas, tangerines etc.) 2. Other fresh fruit (e.g. apple, banana, pear, bunch of grapes, peach etc.)	Less than once a week = Citrus fruit AND other fruits both "Never Or Rarely" OR "Once In 2 Weeks" At keast once a week = Citrus fruit AND/OR other fruits "1-3 Times A Week" OR "4-7 Times A Week" OR "> Once A Day"
Green vegetables			How often, on average, does your child eat or drink the following nowadays? 1. Raw green vegetables 2. Cooked green vegetables	Less than once a week = Raw green vegetables AND Cooked green vegetables both "Never" OR "Less than once per week" At least once a week = Raw green vegetables AND/OR Cooked green vegetables "1-2 times per week" OR "3-6 times per week" OR "Once per day or more often"	How often, on average, does your child eat any green vegetables, nowadays?	Less than once per week = "Never" OR "Less than once per week" At least once a week = "1-2 times per week" OR "3-6 times per week" OR "Once per day or more often"	Thinking about all the food **that you provide** (**Do not include meals provided by school).** How often does your child eat 1. Cabbage, brussel sprouts, spinach, broccoli and other dark green leafy vegetables 2. Other green vegetables (cauliflower, runner beans, leeks, courgettes etc.)	Less than once a week = Dark green leafy vegetables AND other green vegetables both "Never or Rarely" OR "Once In 2 Weeks" At keast once a week = Dark green leafy vegetables AND/OR other green vegetables "1-3 Times A Week" OR "4-7 Times A Week" OR "> Once A Day"
Fast food	In the past 12 months, how often on average, did you eat fast food/burgers	Less than once per week = "Never or only occasionally"	How often, on average, does your child eat burgers nowadays?	Less than once per week = "Never" OR "Less than once per week" At least once a week = "1-2 times	How often, on average, does your child eat burgers, nowadays?	Less than once per week = "Never" OR "Less than once per week" At least once a week = "1-2 times		

		At least once a week = “Once or twice per week” OR “Most or all days”		per week” OR “3-6 times per week” OR “Once per day or more often”		per week” OR “3-6 times per week” OR “Once per day or more often”		
Juice			How often, on average, does your child drink fruit juice nowadays?	Less than once per week = “Never” OR “Less than once per week”<br>At least once a week = “1-2 times per week” OR “3-6 times per week” OR “Once per day or more often”	How often, on average, does your child drink fruit juice, nowadays?	Less than once per week = “Never” OR “Less than once per week”<br>At least once a week = “1-2 times per week” OR “3-6 times per week” OR “Once per day or more often”	Thinking about all the food **that you provide** which she eats during the day, how often does she eat the following foods? **Do not include meals provided by school.**<br>Fruit juice from a tin (including tomato juice) and Pure fruit juice from a carton or freshly squeezed	Less than once a week=<br>Fruit juice from a tin AND pure fruit juice both “Never Or Rarely” OR “Once In 2 Weeks”<br>At keast once a week =<br>Fruit juice from a tin AND/OR pure fruit juice "1-3 Times A Week“ OR “4-7 Times a Week” OR “> Once A Day”
Overweight	Based on Body mass index (kg/m²) compared to table by age	Overweight (BMI≥table) / Not overweight (BMI<table)	Based on Body mass index (kg/m²) compared to table by age	Overweight (BMI≥table) / Not overweight (BMI<table)	Based on Body mass index (kg/m²) compared to table by age	Overweight (BMI≥table) / Not overweight (BMI<table)	Based on Body mass index (kg/m²) compared to table by age	Overweight (BMI≥table) / Not overweight (BMI<table)
Obese	Based on Body mass index (kg/m²) compared to reference table by age	Obese (BMI≥table) / Not obese (BMI<table)	Based on Body mass index (kg/m²) compared to reference table by age	Obese (BMI≥table) / Not obese (BMI<table)	Based on Body mass index (kg/m²) compared to table by age	Obese (BMI≥table) / Not obese (BMI<table)	Based on Body mass index (kg/m²) compared to table by age	Obese (BMI≥table) / Not obese (BMI<table)
Family / birth								
Any older siblings			Derived from number of older siblings	Yes/No	Does your child have older siblings?	Yes/No	Derived from number of older brothers and number of older sisters	Yes/No
Any younger siblings			Derived from number of younger siblings	Yes/No	Does your child have younger siblings?	Yes/No	Derived from number of younger brothers and number of younger sisters	Yes/No
More than 1 sibling			Derived from number of older siblings and number of younger siblings	Yes/No	Derived from number of older brothers, number of older sisters, number of	Yes/No	Derived from number of older brothers, number of older sisters, number of	Yes/No

					younger brothers and number of younger sisters		younger brothers and number of younger sisters	
Breastfed ever			Was your child ever breast fed?	Yes/No	Was your child ever breastfed?	Yes/No	Based on questions on breastfeeding throughout first year	Yes/No
Low birthweight			How much did your child weigh at birth?	Yes = less than 2500g<br>No = 2500g or more			Birthweight in grams	Yes = less than 2500g<br>No = 2500g or more
Premature			Was your child born within 3 weeks of the calculated date?	Premature = “No, more than 3 weeks early”<br>Term = “Yes” OR “No, more than 3 weeks late”	How many months of pregnancy when your child was born?	Yes = 8 months or less / No = 9 months or more	Weeks gestation	Yes if weeks gestation <=37<br>No if weeks gestation>=38
Parental allergic diseases			Has the child’s mother ever had any of the following diseases?<br>Asthma, hayfever, eczema<br>Has the child’s father ever had any of the following diseases?<br>Asthma, hayfever, eczema	Yes = answer yes to any of the questions<br>No = answer no to all of the questions	Dervied from did mother have any allergic diseases, and did father have any allergic diseases?	Yes = answer yes to any of the questions<br>No = answer no to all of the questions	Has the child’s mother ever had any of the following problems: asthma, hayfever, eczema?<br>Has the mother’s partner (if is child’s father) ever had any of the following problems: asthma, hayfever, eczema?	Yes = answer yes to any of the questions<br>No = answer no to all of the questions
Maternal education			For how long did the child’s mother attend school or professional training?<br>School and college	Yes = at least 10 years education<br>No = less than 10 years	For how long did the child’s mother attend school and professional training?	Yes = complete primary education or more<br>No = incomplete primary education OR no education	What is the mother’s highest education qualification?	Yes = any secondary school qualification<br>No = no qualifications
Paternal education			For how long did the child’s father attend school or professional training?<br>School and college	Yes = at least 10 years education<br>No = less than 10 years		Yes = complete primary education or more<br>No = incomplete primary education OR no education	What is the mum’s partner’s highest education qualification?	Yes = any secondary school qualification<br>No = no qualifications
Air-quality								
Any smokers in household			Does anybody, at present, smoke inside your child’s home?	Yes/No	Does anybody, at present, smoke inside your child's home?	Yes/No	Based on how many people living in your household	Yes/No

							(including yourself) are smokers ?	
Maternal smoking			Does your child's mother smoke at present?	Yes/No	Does your child's mother smoke at present?	Yes/No	Based on number of cigarettes smoked	Yes/No
Maternal smoking in first year			Did your child's mother smoke during the child's first year of life	Yes/No	Did your child's mother smoke in the child's first year of life?	Yes/No	Based on number of cigarettes smoked	Yes/No
Maternal smoking while pregnant			Does or did your child's mother smoke during pregnancy with your child?	Yes/No	Did your child's mother smoke during the pregnancy of your child?	Yes/No	Did you smoke regularly at any of the following times during pregnancy:<br>First trimester<br>Second trimester<br>Third trimester	Yes if yes to any<br>No if no to all
Damp	In the past 12 months, have you noticed dampness on walls or ceilings?	Yes/No	Does the child's home have damp spots on the walls or ceiling at present?	Yes/No			How much of a problem is damp or condensation?	1 = no damp or condensation<br>2= not serious<br>3 = fairly serious<br>4 = very serious
Mould	In the past 12 months, have you noticed patches of mould/mildew on any surface of your home?	Yes/No	Does the child's home have visible moulds or fungus at present?	Yes/No			How much of a problem is mould?	1 = no mould<br>2= not serious<br>3 = fairly serious<br>4 = very serious
Damp/ mould	Combine Damp and Mould question	Yes/No	Combine Damp and Mould question	Yes/No	Does the child's home have damp spots on the walls or ceiling, moulds or fungus?	Yes/No	Is there ever any damp, condensation or mould in your home?	Yes/No
Damp in first year			Did the child's home have damp spots on the walls or ceiling in the first year if their life?	Yes/No			How much of a problem is damp or condensation?	1 = no damp or condensation<br>2= not serious<br>3 = fairly serious<br>4 = very serious
Mould in first year			Did the child's home have visible moulds or fungus in the first year if their life?	Yes/No			How much of a problem is mould?	1 = no mould<br>2= not serious<br>3 = fairly serious<br>4 = very serious
Air conditioning	In the past 12 months have you used air conditioning?	Yes/No	Does your child's home have air conditioning at present?	Yes/No	Do you have air conditioning at home?	Yes/No		

ISAAC=International Study of Asthma and Allergies in Childhood; WASP=World Asthma Phenotypes; SCAALA=Social Changes, Asthma and Allergy in Latin America; ALSPAC= Avon Longitudinal Study of Parents and Children; [a] cohort study with multiple surveys at different ages, y=years, m=months, survey at 7y 7m used unless specified; [b] method and allergens from skin prick test detailed in table S2

Table S2: Comparison of skin prick tests between studies

Allergen	ISAAC (>=3mm weal[a], 15 mins)	WASP (>=3mm weal[a], 15 Mins)	SCAALA (>=3mm weal[a], 15 Mins)	ALSPAC (>=2mm weal[a], 10 Mins)
negative control (diluent/saline)	core	core	core	core
positive control (histamine)	core	core	core	core
Mixed grass	core	NZ, Ecuador, Uganda	Ecuador	core
Dermatophagoides pteronyssinus (Der p)	core	Brazil, Ecuador, NZ	Brazil	core
Der p/Der f mix		Uganda	Ecuador	
Cat	core	core	core	core
Egg white				core
Peanut		Uganda		core
Mixed nuts				core
Dog	optional	core	core	group A (1/3)
Horse	optional			group A (1/3)
Mouse				group A (1/3)
Rabbit				group A (1/3)
Guinea pig				group A (1/3)
Hamster				group A (1/3)
Soya				group A (1/3)
Mixed tree pollen	core	NZ		group C (1/3)
Dermatophagoides farinae (Der f)	core	Ecuador, Brazil		group C (1/3)
Alternaria tenuis (fungus)	core	NZ, Ecuador	Ecuador	group C (1/3)
Cladosporium (mould)	optional	NZ		group C (1/3)
Aspergillus fumigatus (mould)				group C (1/3)
Mixed cockroach	optional			removed[b]
Latex				group C (1/3)
Milk				group C (1/3)
Olive	optional			
Parietaria officinalis	optional			
Mixed weeds	optional			
Bird epithelium	optional			
Turkish tree mix	optional			
Penicillium mix		Ecuador, NZ, Uganda		
Blomia Tropicalis		Brazil, Uganda, Ecuador	Brazil	
Mould mix	optional	Uganda		
Mixed fungi			core	
Blatela germanica (cockroach)		Uganda, Brazil	Brazil	
Periplaneta Americana (cockroach)		Ecuador, Brazil	core	
Anisakis simplex (nematode)		Uganda		
Fish				group B (1/3)
Sesame				group B (1/3)
Cashew				group B (1/3)
Almond				group B (1/3)
Walnut				group B (1/3)
Hazelnut				group B (1/3)
Brazil				group B (1/3)
Pecan				group B (1/3)

[a]Weal size is calculated as: (max width+perpendicular width)/2 minus size of negative control weal;
[b]removed from analysis due to problem identified with variable in dataset;
ISAAC=International Study of Asthma and Allergies in Childhood; WASP=World Asthma Phenotypes; SCAALA=Social Changes, Asthma and Allergy in Latin America; ALSPAC= Avon Longitudinal Study of Parents and Children;

Table S3: Associations between missingness and demographics using logistic regression adjusted for study

Exclusion	Sex (male)			Age (years)			Asthma (Case v control)		
	estimate	95% CI	P value	estimate	95% CI	P value	estimate	95% CI	P value
Missing atopy data	1.15	(1.03, 1.29)	0.01	1.07	(1.02, 1.13)	0.009	1.27	(1.09, 1.47)	0.002
Missing symptom data[a]	1.04	(0.98, 1.11)	0.23	0.63	(0.60, 0.65)	<0.001	NA	NA	NA

CI = Confidence interval; NA = not applicable, data not available [a] only ISAAC and ALSPAC included as WASP and SCAALA had no missing data

Table S4: Breakdown of participants by country with income classification from 2000 for ISAAC and 2018 for WASP and 2007 for SCAALA and 1998 for ALSPAC

Country	Participants	Income category
**WASP**		
Brazil (br)	109	LMIC
Ecuador (ec)	242	LMIC
New Zealand (nz)	331	HIC
Uganda (ug)	241	LMIC
**ISAAC**		
Albania (al)	791	LMIC
Brazil (br)	925	LMIC
China (cn)	2001	LMIC
Germany (de)	3861	HIC
Ecuador (ec)	6	LMIC
Estonia (ee)	194	LMIC
Spain (es)	2923	HIC
Georgia (ge)	123	LMIC
Ghana (gh)	1162	LMIC
Greece (gr)	1656	HIC
Hong Kong (hk)	1225	HIC
India (in)	1511	LMIC
Iceland (is)	457	HIC
Italy (it)	1046	HIC
Latvia (lv)	235	LMIC
Netherlands (nl)	1142	HIC
Norway (no)	520	HIC
New Zealand (nz)	952	HIC
Palestine (px)	309	LMIC
Sweden (se)	1509	HIC
Turkey (tr)	345	LMIC
United Kingdom (uk)	763	HIC
**SCAALA**		
Brazil (br)	692	LMIC
Ecuador (ec)	4630	LMIC
**ALSPAC**		
United Kingdom	2840	HIC

ISAAC=International Study of Asthma and Allergies in Childhood; WASP=World Asthma Phenotypes; SCAALA=Social Changes, Asthma and Allergy in Latin America; ALSPAC= Avon Longitudinal Study of Parents and Children; LMIC=Low-middle-income country; HIC=High-income country;

Table S5: Prevalence of potential risk factors by centre / study

Category	Covariable	n	ISAAC Multiple	WASP Brazil	WASP Ecuador	WASP New Zealand	WASP Uganda	WASP total	SCAALA Brazil	SCAALA Ecuador	SCAALA total	ALSPAC UK	Overall Total
Study sample size			23656	109	242	331	241	923	692	4630	5322	2840	32741
Confounder	Male sex	32741	49%	38%	59%	49%	29%	45%	53%	51%	52%	49%	49%
House/ bedding	Feather bedding	14156	18%	4%	18%	37%	.	25%	.	.	.	.	18%
	Synthetic bedding	14155	43%	46%	4%	55%	.	35%	.	.	.	.	43%
	Blankets	15560	44%	14%	56%	47%	.	45%	22%	.	22%	.	43%
	Feather pillow	14780	17%	6%	55%	24%	.	32%	.	.	.	.	17%
	Synthetic pillow	14060	39%	71%	1%	72%	.	47%	.	.	.	.	40%
	Double glazing	20831	55%	.	.	.	.	.	.	.	.	81%	58%
	Double glazing in 1st year	20194	45%	.	.	.	.	.	.	.	.	59%	47%
	Urban	23452	92%	.	.	.	.	.	100%	36%	45%	88%	81%
	Urban in 1st year	16953	89%	.	.	.	.	.	.	.	.	93%	89%
Heating/ cooking	Heating	16979	68%	0%	0%	100%	.	48%	.	.	.	.	67%
	Wood heating	18386	14%	0%	0%	40%	0%	14%	.	.	.	.	14%
	Coal/coke heating	17603	4%	0%	0%	1%	0%	0%	.	.	.	.	4%
	Gas heating	16261	28%	.	.	34%	.	34%	.	.	.	.	28%
	Heating in 1st year	15523	68%	.	.	.	.	.	.	.	.	.	68%
	Wood heating in 1st year	16894	20%	.	.	.	.	.	.	.	.	.	20%
	Coal/coke heating in 1st year	16330	6%	.	.	.	.	.	.	.	.	.	6%
	Cooking electric	20728	45%	1%	15%	.	.	11%	0%	.	0%	.	43%
	Cooking gas	29549	54%	100%	95%	56%	.	77%	99%	96%	97%	61%	63%
	Cooking gas in 1st year	18776	19%	.	.	.	.	.	99%	.	99%	56%	27%
Animals	Pets	20342	43%	51%	63%	77%	30%	58%	37%	60%	57%	70%	51%
	Cats	30397	15%	19%	36%	48%	10%	31%	10%	37%	33%	30%	20%
	Dogs	30379	14%	32%	43%	41%	22%	36%	25%	44%	42%	20%	20%
	Birds	24130	14%	.	.	.	.	.	.	.	.	6%	13%
	Cat in 1st year	21091	9%	.	.	.	.	.	7%	.	7%	29%	12%
	Dog in 1st year	21091	8%	.	.	.	.	.	18%	.	18%	19%	10%
	Bird in 1st year	20399	7%	.	.	.	.	.	.	.	.	5%	7%
	Farm animals	22052	7%	.	77%	.	42%	60%	.	22%	22%	.	11%

Diet/ weight	Meat weekly	26025	88%	.	100%	92%	.	95%	61%	88%	85%	.	87%
	Seafood weekly	26507	57%	.	90%	54%	.	69%	10%	92%	81%	.	62%
	Green vegetables weekly	24540	83%	.	.	.	.	.	31%	73%	68%	78%	79%
	Fruit weekly	25019	90%	69%	96%	98%	.	93%	50%	93%	87%	96%	90%
	Fast food weekly	17878	25%	94%	11%	47%	.	42%	10%	6%	7%	.	20%
	Fruit juice weekly	18553	69%	.	.	.	.	.	55%	.	55%	69%	69%
	Overweight	14615	25%	21%	26%	23%	18%	22%	27%	8%	10%	14%	18%
	Obese	14615	7%	7%	6%	5%	3%	5%	13%	1%	3%	3%	4%
Family/ birth	Older siblings	29536	52%	.	.	.	.	.	62%	76%	75%	52%	56%
	Younger siblings	29470	49%	.	.	.	.	.	42%	77%	72%	52%	53%
	More than 1 sibling	29528	40%	.	.	.	.	.	47%	87%	82%	34%	47%
	Ever breastfed	29588	78%	.	.	.	.	.	96%	98%	97%	80%	82%
	Low birthweight	20176	18%	.	.	.	.	.	.	.	.	4%	16%
	Premature	16875	16%	.	.	.	.	.	8%	.	8%	9%	14%
	Parental allergic diseases	31175	36%	.	.	.	.	.	31%	41%	40%	74%	39%
	Maternal education	18189	79%	.	.	.	.	.	44%	32%	34%	100%	70%
	Paternal education	18389	80%	.	.	.	.	.	100%	38%	46%	100%	74%
Air-quality	Any smokers	29371	45%	.	.	.	.	.	21%	44%	41%	29%	43%
	Maternal smoking	30869	25%	.	.	.	.	.	11%	13%	13%	16%	22%
	Maternal smoking in 1st year	27364	19%	.	.	.	.	.	10%	11%	11%	17%	17%
	Maternal smoking during pregnancy	28545	12%	.	.	.	.	.	10%	10%	10%	16%	12%
	Damp	17670	14%	38%	38%	15%	30%	28%	.	.	.	90%	20%
	Mould	15021	9%	40%	22%	54%	35%	39%	.	.	.	52%	15%
	Damp or mould	15512	18%	50%	42%	55%	48%	49%	60%	.	60%	92%	28%
	Damp in 1st year	17763	15%	.	.	.	.	.	.	.	.	49%	20%
	Mould in 1st year	14599	10%	.	.	.	.	.	.	.	.	25%	13%
	Air conditioning	14910	29%	2%	.	3%	.	3%	1%	.	1%	.	27%

ISAAC=International Study of Asthma and Allergies in Childhood; WASP=World Asthma Phenotypes; SCAALA=Social Changes, Asthma and Allergy in Latin America; ALSPAC= Avon Longitudinal Study of Parents and Children

Table S6: Results from mixed effects logistic regression with random intercept for centre, adjusted for age, sex, study and country income group

Category	Risk factor	Pathway 1 X to NAA		Pathway 2 X to Y		Pathway 3 Y to AA		Pathway 4 X to AA				Pathway 5 NAA to AA	
		n	OR (95% CI)	*n*	OR (95% CI)	*n*	OR (95% CI)	*n*	OR (95% CI)	predicted OR[a]	% of predicted	*n*	OR (95% CI)
House/ bedding	Feather bedding	10567	0.63 (0.52, 0.76)	11018	0.87 (0.76, 0.99)	3589	0.64 (0.52, 0.79)	10371	0.51 (0.42, 0.60)	0.56	0.9	3138	0.75 (0.59, 0.95)
	Synthetic bedding	10566	1.33 (1.16, 1.53)	11017	1.18 (1.05, 1.31)	3589	1.51 (1.28, 1.79)	10370	1.87 (1.62, 2.16)	1.78	1.1	3138	1.36 (1.14, 1.63)
	Blankets	11909	1.08 (0.94, 1.23)	12260	0.88 (0.79, 0.98)	3651	0.97 (0.82, 1.15)	11519	0.81 (0.70, 0.93)	0.85	0.9	3300	0.74 (0.62, 0.88)
	Feather pillow	11206	0.80 (0.66, 0.96)	11618	0.88 (0.77, 1.01)	3574	0.64 (0.50, 0.81)	10946	0.52 (0.42, 0.63)	0.56	0.9	3162	0.72 (0.57, 0.92)
	Synthetic pillow	10500	1.04 (0.90, 1.20)	10950	1.00 (0.89, 1.11)	3560	1.34 (1.13, 1.58)	10286	1.43 (1.24, 1.65)	1.34	1.1	3110	1.45 (1.21, 1.74)
	Double glazing	15773	0.84 (0.74, 0.96)	17402	0.95 (0.86, 1.04)	5058	0.98 (0.83, 1.15)	15498	0.93 (0.81, 1.07)	0.93	1.0	3429	1.15 (0.97, 1.37)
	Double glazing in 1st year	15344	1.00 (0.88, 1.13)	16874	0.98 (0.89, 1.07)	4850	0.97 (0.83, 1.13)	15068	0.95 (0.83, 1.08)	0.94	1.0	3320	0.99 (0.84, 1.17)
	Urban	18519	0.84 (0.73, 0.96)	19188	1.02 (0.90, 1.17)	4933	0.87 (0.70, 1.09)	17609	0.95 (0.79, 1.13)	0.89	1.1	4264	1.05 (0.85, 1.30)
	Urban in 1st year	13120	0.86 (0.71, 1.03)	13973	1.13 (0.97, 1.31)	3833	0.94 (0.73, 1.21)	12730	1.02 (0.83, 1.25)	1.06	1.0	2980	1.12 (0.87, 1.44)
Heating/ cooking	Heating	12772	1.02 (0.83, 1.25)	14360	0.87 (0.75, 1.01)	4207	1.18 (0.93, 1.51)	12761	0.98 (0.79, 1.22)	1.03	1.0	2619	0.96 (0.72, 1.29)
	Wood heating	13677	0.95 (0.79, 1.15)	14939	0.90 (0.77, 1.06)	4709	1.04 (0.82, 1.32)	13538	0.90 (0.73, 1.09)	0.93	1.0	3447	1.02 (0.81, 1.28)
	Coal/coke heating	13110	1.09 (0.74, 1.61)	14253	1.01 (0.77, 1.31)	4493	1.75 (1.11, 2.78)	12948	1.86 (1.26, 2.75)	1.76	1.1	3350	1.00 (0.69, 1.44)
	Gas heating	12152	0.94 (0.79, 1.12)	13439	1.06 (0.94, 1.19)	4109	0.91 (0.75, 1.11)	12030	0.99 (0.83, 1.17)	0.96	1.0	2822	1.11 (0.90, 1.37)
	Heating in 1st year	11900	1.01 (0.82, 1.25)	13492	0.86 (0.74, 1.00)	3623	1.20 (0.94, 1.53)	11747	0.99 (0.80, 1.24)	1.04	1.0	2031	0.93 (0.68, 1.26)
	Wood heating in 1st year	12799	0.97 (0.80, 1.18)	14263	0.88 (0.75, 1.03)	4095	1.24 (0.95, 1.63)	12546	1.00 (0.81, 1.24)	1.09	0.9	2631	1.06 (0.82, 1.35)
	Coal/coke heating in 1st year	12385	1.16 (0.81, 1.66)	13766	0.92 (0.73, 1.15)	3945	1.39 (0.90, 2.13)	12117	1.37 (0.95, 1.98)	1.27	1.1	2564	0.92 (0.62, 1.37)
	Cooking electric	15748	1.09 (0.92, 1.30)	17160	1.03 (0.92, 1.15)	4980	0.93 (0.77, 1.11)	15372	0.98 (0.84, 1.16)	0.96	1.0	3568	0.93 (0.76, 1.15)
	Cooking gas	22738	0.91 (0.80, 1.03)	24210	0.96 (0.88, 1.06)	6811	1.09 (0.94, 1.27)	22129	1.04 (0.91, 1.18)	1.05	1.0	5339	1.07 (0.91, 1.27)
	Cooking gas in 1st year	14074	0.90 (0.78, 1.05)	15223	0.93 (0.82, 1.05)	4702	0.98 (0.81, 1.17)	13801	0.92 (0.79, 1.07)	0.90	1.0	3553	1.02 (0.84, 1.23)
Animals	Pets	16101	1.07 (0.97, 1.18)	16255	0.87 (0.79, 0.95)	4241	1.06 (0.92, 1.22)	15574	0.85 (0.75, 0.95)	0.91	0.9	4087	0.87 (0.76, 1.00)
	Cats	23390	1.01 (0.91, 1.12)	25076	0.81 (0.74, 0.89)	7007	1.02 (0.88, 1.19)	22939	0.80 (0.71, 0.91)	0.83	1.0	5321	0.83 (0.71, 0.98)
	Dogs	23379	1.20 (1.08, 1.33)	25058	0.90 (0.82, 0.99)	7000	1.08 (0.93, 1.25)	22926	0.91 (0.80, 1.03)	0.97	0.9	5321	0.81 (0.70, 0.94)
	Birds	18376	1.00 (0.85, 1.18)	20396	0.85 (0.75, 0.96)	5754	1.07 (0.87, 1.30)	18266	0.90 (0.76, 1.07)	0.90	1.0	3734	0.92 (0.74, 1.15)
	Cat in 1st year	15808	1.02 (0.87, 1.19)	17447	0.83 (0.72, 0.95)	5283	1.08 (0.89, 1.32)	15618	0.90 (0.77, 1.05)	0.90	1.0	3644	0.91 (0.74, 1.12)
	Dog in 1st year	15808	1.10 (0.94, 1.29)	17447	0.73 (0.63, 0.84)	5283	1.41 (1.15, 1.72)	15618	0.94 (0.80, 1.10)	1.02	0.9	3644	0.90 (0.73, 1.11)
	Bird in 1st year	15330	1.38 (1.13, 1.68)	17034	0.92 (0.78, 1.08)	5069	1.47 (1.15, 1.87)	15215	1.16 (0.94, 1.42)	1.35	0.9	3365	0.84 (0.65, 1.09)
	Farm animals	17447	1.06 (0.91, 1.23)	18267	1.02 (0.88, 1.19)	4605	1.00 (0.77, 1.30)	16580	0.86 (0.69, 1.06)	1.02	0.8	3785	0.86 (0.68, 1.08)
Diet/ weight	Meat weekly	20325	1.03 (0.88, 1.20)	21744	0.93 (0.81, 1.05)	5700	0.96 (0.76, 1.21)	19588	0.97 (0.79, 1.19)	0.89	1.1	4281	0.99 (0.78, 1.24)
	Seafood weekly	20653	0.99 (0.88, 1.11)	22073	1.04 (0.95, 1.13)	5854	0.87 (0.75, 1.00)	19941	0.96 (0.84, 1.08)	0.90	1.1	4434	0.99 (0.84, 1.16)
	Green vegetables weekly	19289	1.01 (0.91, 1.13)	20204	1.00 (0.91, 1.11)	5251	1.06 (0.90, 1.25)	18441	1.05 (0.91, 1.20)	1.06	1.0	4336	1.02 (0.86, 1.21)
	Fruit weekly	19436	0.80 (0.70, 0.93)	20215	1.14 (0.99, 1.31)	5583	0.78 (0.63, 0.97)	18730	0.91 (0.76, 1.09)	0.89	1.0	4804	1.01 (0.82, 1.25)
	Fast food weekly	14118	1.16 (1.02, 1.33)	14261	1.09 (0.97, 1.23)	3760	0.96 (0.80, 1.15)	13385	1.08 (0.92, 1.26)	1.05	1.0	3617	0.88 (0.73, 1.07)
	Fruit juice weekly	13926	1.15 (1.02, 1.30)	14940	1.08 (0.98, 1.19)	4627	0.82 (0.71, 0.96)	13577	0.92 (0.81, 1.04)	0.89	1.0	3613	0.80 (0.68, 0.94)
	Overweight	11087	1.31 (1.14, 1.50)	10709	1.08 (0.94, 1.24)	3528	1.24 (1.03, 1.51)	10635	1.24 (1.07, 1.44)	1.34	0.9	3906	0.90 (0.76, 1.07)
	Obese	11087	1.60 (1.28, 2.01)	10709	0.93 (0.70, 1.22)	3528	1.55 (1.11, 2.16)	10635	1.35 (1.05, 1.74)	1.43	0.9	3906	0.87 (0.66, 1.13)
Family/ birth	Older siblings	23027	1.02 (0.93, 1.11)	24657	0.93 (0.87, 1.00)	6509	1.05 (0.93, 1.18)	22216	0.89 (0.81, 0.99)	0.97	0.9	4879	0.87 (0.76, 0.99)
	Younger siblings	22996	1.03 (0.94, 1.13)	24602	0.96 (0.89, 1.03)	6474	0.95 (0.84, 1.07)	22182	0.95 (0.85, 1.05)	0.91	1.0	4868	0.91 (0.80, 1.03)
	More than 1 sibling	23030	1.08 (0.98, 1.19)	24646	0.90 (0.83, 0.97)	6498	1.08 (0.95, 1.23)	22216	0.90 (0.80, 1.00)	0.97	0.9	4882	0.84 (0.73, 0.97)
	Ever breastfed	23039	0.75 (0.67, 0.85)	24632	0.95 (0.87, 1.04)	6549	0.91 (0.79, 1.06)	22239	0.92 (0.81, 1.04)	0.87	1.1	4956	1.17 (0.99, 1.38)
	Low birthweight	15397	1.25 (1.05, 1.49)	16528	0.94 (0.81, 1.07)	4779	1.09 (0.86, 1.38)	15081	1.03 (0.85, 1.26)	1.02	1.0	3648	0.80 (0.62, 1.03)
	Premature	12984	1.42 (1.19, 1.69)	13638	0.85 (0.73, 0.99)	3891	1.28 (1.00, 1.64)	12599	1.05 (0.86, 1.28)	1.09	1.0	3237	0.71 (0.56, 0.91)
	Parental allergic diseases	24285	2.45 (2.24, 2.69)	26107	1.38 (1.29, 1.49)	6890	2.05 (1.81, 2.31)	23503	2.81 (2.53, 3.12)	2.83	1.0	5068	1.28 (1.12, 1.47)
	Maternal education	14692	0.90 (0.79, 1.04)	15076	1.02 (0.90, 1.15)	3497	1.09 (0.88, 1.36)	13907	1.21 (0.99, 1.48)	1.11	1.1	3113	1.40 (1.11, 1.76)
	Paternal education	14865	0.93 (0.80, 1.07)	15243	1.05 (0.93, 1.20)	3524	0.79 (0.63, 1.00)	14061	0.87 (0.71, 1.07)	0.84	1.0	3146	0.98 (0.76, 1.26)

Air-quality	Any smokers	22895	1.24 (1.14, 1.36)	24500	0.87 (0.81, 0.93)	6476	1.04 (0.93, 1.17)	22118	0.90 (0.81, 0.99)	0.90	1.0	4871	0.74 (0.65, 0.84)
	Maternal smoking	24064	1.46 (1.32, 1.62)	25823	0.84 (0.78, 0.92)	6805	1.22 (1.07, 1.40)	23302	1.04 (0.93, 1.17)	1.03	1.0	5046	0.76 (0.65, 0.87)
	Maternal smoking in 1st year	21441	1.56 (1.40, 1.74)	22776	0.87 (0.78, 0.96)	5923	1.37 (1.18, 1.60)	20641	1.16 (1.02, 1.31)	1.19	1.0	4588	0.74 (0.63, 0.87)
	Maternal smoking during pregnancy	22270	1.56 (1.39, 1.75)	23766	0.89 (0.80, 1.00)	6275	1.40 (1.18, 1.65)	21483	1.22 (1.06, 1.40)	1.24	1.0	4779	0.83 (0.70, 0.98)
	Damp	13028	1.64 (1.41, 1.90)	13924	0.87 (0.75, 1.00)	4642	1.51 (1.22, 1.86)	12896	1.26 (1.06, 1.50)	1.31	1.0	3746	0.92 (0.76, 1.12)
	Mould	11302	1.51 (1.29, 1.75)	11346	0.83 (0.69, 1.00)	3719	1.51 (1.20, 1.91)	11037	1.27 (1.07, 1.51)	1.26	1.0	3675	0.90 (0.74, 1.09)
	Damp or mould	11623	1.56 (1.36, 1.79)	11712	0.92 (0.79, 1.06)	3889	1.47 (1.20, 1.80)	11275	1.31 (1.11, 1.54)	1.35	1.0	3800	0.99 (0.83, 1.19)
	Damp in 1st year	13363	1.51 (1.32, 1.72)	14423	0.93 (0.82, 1.05)	4400	1.65 (1.39, 1.96)	13065	1.55 (1.35, 1.79)	1.53	1.0	3340	1.06 (0.89, 1.26)
	Mould in 1st year	11274	1.49 (1.29, 1.73)	11514	0.92 (0.78, 1.10)	3325	1.69 (1.35, 2.11)	10839	1.57 (1.33, 1.86)	1.56	1.0	3085	1.06 (0.87, 1.30)
	Air conditioning	11116	0.92 (0.76, 1.12)	12060	1.10 (0.98, 1.25)	3794	0.86 (0.70, 1.06)	10888	1.01 (0.84, 1.22)	0.95	1.1	2850	1.03 (0.79, 1.34)

OR=Odds Ratio; CI=Confidence Interval; X: non-atopic, non-asthmatic; NAA: non-atopic asthma; Y:atopic, non-asthmatic; AA: atopic asthma; [a]the OR for pathway 2 multiplied by the OR pathway 3;

Table S7: Mixed effects logistic regression models stratified by country income group, with random intercept for centre, adjusted for age, sex and study

Category	Risk factor	Pathway 1 X to NAA				Pathway 2 X to Y				Pathway 3 Y to AA			
		n	LMIC OR (95% CI)	HIC OR (95% CI)	Inter-action *P*-value	*n*	LMIC OR (95% CI)	HIC OR (95% CI)	Inter-action *P*-value	*n*	LMIC OR (95% CI)	HIC OR (95% CI)	Inter-action *P*-value
House/ bedding	Feather bedding	10567	0.83 (0.52, 1.31)	0.59 (0.48, 0.73)	0.20	11018	0.82 (0.57, 1.19)	0.88 (0.76, 1.01)	0.76	3589	0.85 (0.43, 1.66)	0.62 (0.50, 0.78)	0.40
	Synthetic bedding	10566	0.92 (0.72, 1.18)	1.59 (1.34, 1.88)	0.00	11017	1.25 (0.96, 1.64)	1.16 (1.03, 1.31)	0.60	3589	1.43 (0.92, 2.23)	1.53 (1.27, 1.83)	0.79
	Blankets	11909	0.90 (0.74, 1.10)	1.27 (1.05, 1.53)	0.01	12260	0.80 (0.64, 1.00)	0.91 (0.80, 1.03)	0.32	3651	1.38 (0.98, 1.94)	0.86 (0.71, 1.05)	0.02
	Feather pillow	11206	1.12 (0.76, 1.64)	0.72 (0.58, 0.90)	0.05	11618	0.92 (0.67, 1.26)	0.87 (0.75, 1.02)	0.78	3574	1.23 (0.69, 2.22)	0.56 (0.43, 0.73)	0.02
	Synthetic pillow	10500	0.79 (0.55, 1.14)	1.10 (0.94, 1.29)	0.11	10950	1.18 (0.89, 1.56)	0.97 (0.86, 1.09)	0.22	3560	1.06 (0.64, 1.76)	1.38 (1.16, 1.65)	0.34
	Double glazing	15773	0.81 (0.63, 1.05)	0.85 (0.73, 0.99)	0.76	17402	0.87 (0.71, 1.06)	0.97 (0.87, 1.08)	0.34	5058	1.21 (0.81, 1.81)	0.94 (0.79, 1.12)	0.25
	Double glazing in 1st year	15344	0.85 (0.66, 1.11)	1.04 (0.90, 1.20)	0.19	16874	0.95 (0.75, 1.20)	0.98 (0.89, 1.09)	0.82	4850	0.92 (0.58, 1.47)	0.97 (0.82, 1.15)	0.83
	Urban	18519	0.79 (0.66, 0.93)	0.92 (0.74, 1.14)	0.26	19188	0.84 (0.70, 1.01)	1.29 (1.06, 1.58)	0.00	4933	1.38 (0.94, 2.03)	0.70 (0.53, 0.91)	0.00
	Urban in 1st year	13120	0.71 (0.51, 0.99)	0.94 (0.74, 1.17)	0.18	13973	1.27 (0.94, 1.70)	1.08 (0.90, 1.29)	0.36	3833	0.85 (0.47, 1.54)	0.96 (0.73, 1.26)	0.72
Heating/ cooking	Heating	12772	1.02 (0.77, 1.33)	1.02 (0.75, 1.39)	0.99	14360	0.99 (0.76, 1.29)	0.82 (0.69, 0.98)	0.25	4207	0.95 (0.57, 1.60)	1.26 (0.96, 1.67)	0.35
	Wood heating	13677	1.07 (0.82, 1.40)	0.85 (0.65, 1.10)	0.21	14939	1.18 (0.82, 1.70)	0.85 (0.71, 1.01)	0.10	4709	1.10 (0.63, 1.93)	1.02 (0.79, 1.33)	0.83
	Coal/coke heating	13110	1.35 (0.83, 2.18)	0.72 (0.35, 1.51)	0.17	14253	0.92 (0.67, 1.27)	1.20 (0.77, 1.85)	0.34	4493	1.44 (0.68, 3.05)	1.98 (1.10, 3.54)	0.51
	Gas heating	12152	0.82 (0.56, 1.19)	0.98 (0.81, 1.20)	0.39	13439	1.04 (0.80, 1.35)	1.06 (0.93, 1.21)	0.90	4109	0.93 (0.53, 1.60)	0.91 (0.73, 1.12)	0.95
	Heating in 1st year	11900	1.16 (0.87, 1.55)	0.87 (0.65, 1.18)	0.18	13492	0.81 (0.63, 1.04)	0.89 (0.74, 1.07)	0.54	3623	0.96 (0.59, 1.57)	1.30 (0.98, 1.72)	0.30
	Wood heating in 1st year	12799	1.24 (0.95, 1.63)	0.76 (0.57, 1.00)	0.01	14263	1.13 (0.83, 1.53)	0.80 (0.67, 0.96)	0.06	4095	1.08 (0.61, 1.92)	1.29 (0.95, 1.75)	0.59
	Coal/coke heating in 1st year	12385	1.47 (0.91, 2.38)	0.85 (0.47, 1.53)	0.15	13766	0.86 (0.65, 1.14)	1.03 (0.70, 1.51)	0.47	3945	1.75 (0.90, 3.41)	1.17 (0.66, 2.07)	0.37
	Cooking electric	15748	1.32 (0.90, 1.95)	1.04 (0.86, 1.27)	0.28	17160	1.14 (0.89, 1.45)	1.00 (0.89, 1.13)	0.36	4980	0.73 (0.49, 1.08)	0.99 (0.81, 1.21)	0.17
	Cooking gas	22738	0.68 (0.53, 0.87)	1.01 (0.87, 1.16)	0.01	24210	1.02 (0.84, 1.23)	0.95 (0.85, 1.06)	0.53	6811	1.85 (1.24, 2.77)	0.99 (0.85, 1.17)	0.00
	Cooking gas in 1st year	14074	0.76 (0.50, 1.16)	0.93 (0.79, 1.09)	0.39	15223	0.83 (0.60, 1.16)	0.94 (0.83, 1.08)	0.50	4702	1.16 (0.62, 2.15)	0.96 (0.79, 1.16)	0.58
Animals	Pets	16101	1.16 (1.00, 1.34)	0.99 (0.86, 1.14)	0.12	16255	0.89 (0.76, 1.04)	0.85 (0.76, 0.96)	0.69	4241	1.40 (1.05, 1.87)	0.96 (0.82, 1.14)	0.03
	Cats	23390	0.92 (0.79, 1.08)	1.09 (0.94, 1.25)	0.13	25076	0.80 (0.67, 0.94)	0.81 (0.73, 0.92)	0.81	7007	1.54 (1.13, 2.09)	0.90 (0.76, 1.07)	0.00
	Dogs	23379	1.21 (1.05, 1.40)	1.19 (1.03, 1.37)	0.85	25058	0.94 (0.80, 1.11)	0.88 (0.78, 0.98)	0.48	7000	1.41 (1.04, 1.91)	0.99 (0.83, 1.17)	0.05
	Birds	18376	1.24 (0.87, 1.78)	0.95 (0.79, 1.14)	0.19	20396	0.91 (0.66, 1.26)	0.84 (0.74, 0.95)	0.62	5754	1.39 (0.77, 2.53)	1.03 (0.83, 1.27)	0.35
	Cat in 1st year	15808	1.99 (1.36, 2.93)	0.91 (0.77, 1.08)	0.00	17447	0.93 (0.69, 1.26)	0.81 (0.69, 0.94)	0.41	5283	1.70 (1.03, 2.80)	1.00 (0.80, 1.24)	0.05
	Dog in 1st year	15808	1.82 (1.27, 2.62)	0.99 (0.83, 1.18)	0.00	17447	0.93 (0.64, 1.35)	0.70 (0.60, 0.82)	0.16	5283	3.21 (1.91, 5.37)	1.20 (0.96, 1.50)	0.00
	Bird in 1st year	15330	1.68 (0.94, 3.01)	1.34 (1.09, 1.66)	0.48	17034	1.07 (0.68, 1.69)	0.90 (0.76, 1.07)	0.47	5069	3.26 (1.62, 6.58)	1.32 (1.02, 1.71)	0.02
	Farm animals	17447	0.99 (0.84, 1.18)	1.28 (0.97, 1.70)	0.13	18267	1.24 (1.02, 1.50)	0.76 (0.59, 0.97)	0.00	4605	0.98 (0.67, 1.43)	1.01 (0.70, 1.47)	0.90
Diet/ weight	Meat weekly	20325	1.11 (0.93, 1.32)	0.81 (0.60, 1.08)	0.07	21744	0.94 (0.79, 1.11)	0.91 (0.75, 1.11)	0.86	5700	1.06 (0.76, 1.47)	0.87 (0.63, 1.20)	0.41
	Seafood weekly	20653	1.05 (0.88, 1.25)	0.94 (0.81, 1.09)	0.34	22073	0.94 (0.80, 1.09)	1.08 (0.98, 1.20)	0.13	5854	0.82 (0.61, 1.11)	0.88 (0.75, 1.04)	0.69
	Fruit weekly	19436	0.79 (0.66, 0.94)	0.83 (0.66, 1.06)	0.71	20215	1.24 (1.00, 1.52)	1.07 (0.89, 1.29)	0.31	5583	0.77 (0.55, 1.08)	0.79 (0.60, 1.03)	0.92
	Green vegetables weekly	19289	1.06 (0.91, 1.23)	0.96 (0.81, 1.13)	0.38	20204	1.01 (0.86, 1.19)	1.00 (0.88, 1.14)	0.94	5251	1.03 (0.77, 1.39)	1.07 (0.88, 1.30)	0.85
	Fast food weekly	14118	1.06 (0.87, 1.29)	1.27 (1.05, 1.53)	0.20	14261	1.20 (0.97, 1.49)	1.05 (0.91, 1.21)	0.33	3760	0.63 (0.42, 0.95)	1.08 (0.87, 1.32)	0.02
	Fruit juice weekly	13926	1.18 (0.97, 1.43)	1.13 (0.96, 1.33)	0.75	14940	1.21 (1.03, 1.42)	1.01 (0.89, 1.14)	0.08	4627	0.57 (0.42, 0.76)	0.94 (0.79, 1.12)	0.00
	Overweight	11087	0.90 (0.72, 1.12)	1.66 (1.40, 1.98)	0.00	10709	1.24 (0.96, 1.61)	1.02 (0.87, 1.21)	0.22	3528	1.12 (0.75, 1.67)	1.29 (1.03, 1.60)	0.54
	Obese	11087	0.98 (0.65, 1.48)	2.02 (1.54, 2.65)	0.00	10709	1.01 (0.60, 1.71)	0.90 (0.65, 1.24)	0.71	3528	1.56 (0.81, 3.01)	1.54 (1.04, 2.28)	0.98
Family/ birth	Older siblings	23027	1.00 (0.87, 1.15)	1.03 (0.91, 1.16)	0.75	24657	1.04 (0.90, 1.21)	0.90 (0.83, 0.97)	0.07	6509	0.94 (0.72, 1.23)	1.07 (0.94, 1.22)	0.39
	Younger siblings	22996	1.12 (0.98, 1.29)	0.97 (0.86, 1.09)	0.12	24602	0.86 (0.75, 1.00)	0.99 (0.91, 1.07)	0.11	6474	0.91 (0.70, 1.19)	0.96 (0.84, 1.09)	0.76
	More than 1 sibling	23030	1.17 (1.00, 1.37)	1.03 (0.90, 1.17)	0.21	24646	1.01 (0.86, 1.19)	0.87 (0.79, 0.95)	0.12	6498	0.91 (0.67, 1.24)	1.12 (0.98, 1.29)	0.23
	Ever breastfed	23039	0.72 (0.56, 0.92)	0.76 (0.67, 0.88)	0.65	24632	0.91 (0.75, 1.11)	0.96 (0.87, 1.06)	0.64	6549	0.95 (0.65, 1.37)	0.91 (0.77, 1.07)	0.85
	Low birthweight	15397	1.07 (0.81, 1.39)	1.40 (1.12, 1.76)	0.13	16528	0.83 (0.63, 1.08)	0.98 (0.83, 1.15)	0.30	4779	0.84 (0.48, 1.49)	1.15 (0.89, 1.48)	0.33
	Premature	12984	1.28 (0.96, 1.72)	1.50 (1.20, 1.88)	0.40	13638	0.84 (0.64, 1.10)	0.85 (0.70, 1.03)	0.92	3891	1.40 (0.88, 2.22)	1.24 (0.92, 1.66)	0.66
	Parental allergic diseases	24285	3.51 (3.06, 4.02)	1.80 (1.59, 2.04)	0.00	26107	1.15 (0.99, 1.34)	1.47 (1.35, 1.59)	0.01	6890	2.45 (1.89, 3.18)	1.94 (1.69, 2.23)	0.12
	Maternal education	14692	0.95 (0.80, 1.12)	0.82 (0.64, 1.05)	0.34	15076	1.12 (0.92, 1.35)	0.95 (0.81, 1.12)	0.21	3497	0.95 (0.68, 1.32)	1.23 (0.91, 1.66)	0.26

	Paternal education	14865	0.96 (0.81, 1.14)	0.86 (0.67, 1.10)	0.47	15243	1.03 (0.84, 1.25)	1.07 (0.91, 1.26)	0.72	3524	0.68 (0.46, 1.02)	0.86 (0.64, 1.14)	0.36
Air-quality	Any smokers	22895	1.12 (0.98, 1.27)	1.38 (1.21, 1.56)	0.02	24500	0.96 (0.85, 1.09)	0.82 (0.76, 0.90)	0.05	6476	1.02 (0.81, 1.30)	1.05 (0.91, 1.20)	0.87
	Maternal smoking	24064	1.27 (1.07, 1.51)	1.58 (1.39, 1.79)	0.05	25823	0.92 (0.74, 1.14)	0.83 (0.76, 0.91)	0.39	6805	1.37 (0.95, 1.96)	1.20 (1.04, 1.39)	0.52
	Maternal smoking in 1st year	21441	1.48 (1.23, 1.79)	1.60 (1.40, 1.84)	0.50	22776	1.04 (0.81, 1.32)	0.84 (0.75, 0.93)	0.12	5923	1.45 (0.97, 2.16)	1.36 (1.15, 1.61)	0.78
	Maternal smoking during pregnancy	22270	1.55 (1.27, 1.88)	1.57 (1.35, 1.81)	0.93	23766	1.03 (0.80, 1.33)	0.86 (0.76, 0.98)	0.23	6275	1.43 (0.94, 2.18)	1.39 (1.16, 1.67)	0.91
	Damp	13028	1.63 (1.34, 1.98)	1.65 (1.31, 2.08)	0.94	13924	0.84 (0.66, 1.06)	0.89 (0.74, 1.06)	0.70	4642	1.86 (1.27, 2.72)	1.38 (1.07, 1.77)	0.20
	Mould	11302	1.41 (1.14, 1.75)	1.61 (1.30, 2.00)	0.39	11346	0.70 (0.50, 1.00)	0.89 (0.72, 1.11)	0.25	3719	1.87 (1.12, 3.10)	1.43 (1.11, 1.86)	0.37
	Damp or mould	11623	1.50 (1.26, 1.79)	1.66 (1.33, 2.08)	0.49	11712	0.86 (0.68, 1.09)	0.96 (0.79, 1.16)	0.49	3889	1.77 (1.25, 2.49)	1.33 (1.03, 1.72)	0.19
	Damp in 1st year	13363	1.81 (1.47, 2.25)	1.35 (1.15, 1.60)	0.03	14423	0.83 (0.66, 1.03)	0.98 (0.85, 1.14)	0.20	4400	1.89 (1.30, 2.75)	1.59 (1.31, 1.93)	0.43
	Mould in 1st year	11274	1.61 (1.27, 2.04)	1.42 (1.17, 1.73)	0.44	11514	0.75 (0.52, 1.08)	0.98 (0.81, 1.19)	0.20	3325	2.03 (1.16, 3.57)	1.63 (1.28, 2.08)	0.48
	Air conditioning	11116	0.92 (0.64, 1.33)	0.92 (0.73, 1.15)	0.99	12060	1.34 (1.08, 1.66)	1.01 (0.86, 1.17)	0.03	3794	0.77 (0.52, 1.15)	0.90 (0.70, 1.15)	0.52

X: non-atopic, non-asthmatic; NAA: non-atopic asthma; Y:atopic, non-asthmatic; AA: atopic asthma ; LMIC: low-middle-income country; HIC: high-income country; OR=Odds Ratio; CI=Confidence Interval; X: non-atopic, non-asthmatic; NAA: non-atopic asthma; Y:atopic, non-asthmatic; AA: atopic asthma; [a]the OR for pathway 2 multiplied by the OR pathway 3;

Table S7 continued

Category	Risk factor	Pathway 4 X to AA						Pathway 5 NAA to AA			
		n	LMIC OR (95% CI)	HIC OR (95% CI)	Inter-action *P*-value	LMIC predicted OR[a]	HIC predicted OR[a]	*n*	LMIC OR (95% CI)	HIC OR (95% CI)	Inter-action *P*-value
House/ bedding	Feather bedding	10371	0.49 (0.29, 0.83)	0.51 (0.42, 0.61)	0.88	0.70	0.55	3138	0.66 (0.36, 1.21)	0.77 (0.59, 1.00)	0.66
	Synthetic bedding	10370	1.79 (1.26, 2.55)	1.88 (1.61, 2.21)	0.80	1.79	1.77	3138	1.50 (1.09, 2.07)	1.30 (1.05, 1.61)	0.46
	Blankets	11519	0.87 (0.67, 1.15)	0.78 (0.66, 0.93)	0.51	1.10	0.78	3300	0.91 (0.70, 1.18)	0.63 (0.50, 0.80)	0.05
	Feather pillow	10946	1.12 (0.70, 1.77)	0.43 (0.34, 0.54)	0.00	1.13	0.49	3162	1.15 (0.77, 1.71)	0.57 (0.43, 0.76)	0.01
	Synthetic pillow	10286	1.74 (1.12, 2.72)	1.40 (1.20, 1.63)	0.36	1.25	1.34	3110	1.66 (1.14, 2.42)	1.40 (1.14, 1.71)	0.44
	Double glazing	15498	0.90 (0.62, 1.30)	0.94 (0.81, 1.09)	0.84	1.05	0.91	3429	1.25 (0.89, 1.75)	1.12 (0.91, 1.36)	0.57
	Double glazing in 1st year	15068	0.76 (0.50, 1.15)	0.97 (0.84, 1.11)	0.28	0.88	0.96	3320	1.31 (0.90, 1.90)	0.92 (0.77, 1.11)	0.10
	Urban	17609	1.09 (0.78, 1.52)	0.90 (0.73, 1.11)	0.34	1.16	0.90	4264	1.24 (0.88, 1.75)	0.95 (0.72, 1.24)	0.22
	Urban in 1st year	12730	1.14 (0.69, 1.87)	0.99 (0.79, 1.25)	0.63	1.08	1.04	2980	1.43 (0.91, 2.24)	1.00 (0.74, 1.36)	0.21
Heating/ cooking	Heating	12761	0.94 (0.62, 1.42)	1.00 (0.77, 1.29)	0.82	0.94	1.04	2619	1.03 (0.65, 1.62)	0.92 (0.62, 1.35)	0.71
	Wood heating	13538	1.23 (0.80, 1.87)	0.82 (0.66, 1.03)	0.10	1.30	0.87	3447	1.00 (0.71, 1.41)	1.04 (0.76, 1.41)	0.87
	Coal/coke heating	12948	1.47 (0.77, 2.82)	2.14 (1.32, 3.47)	0.37	1.32	2.37	3350	0.70 (0.45, 1.08)	3.02 (1.31, 6.93)	0.00
	Gas heating	12030	0.95 (0.60, 1.51)	1.00 (0.83, 1.20)	0.85	0.97	0.96	2822	1.31 (0.89, 1.91)	1.03 (0.81, 1.33)	0.31
	Heating in 1st year	11747	0.76 (0.50, 1.17)	1.10 (0.85, 1.43)	0.16	0.78	1.16	2031	0.66 (0.39, 1.10)	1.14 (0.77, 1.69)	0.09
	Wood heating in 1st year	12546	1.06 (0.69, 1.64)	0.98 (0.77, 1.26)	0.76	1.22	1.04	2631	0.77 (0.54, 1.11)	1.44 (1.01, 2.04)	0.02
	Coal/coke heating in 1st year	12117	1.45 (0.82, 2.57)	1.32 (0.82, 2.13)	0.80	1.51	1.20	2564	0.73 (0.45, 1.18)	1.50 (0.73, 3.09)	0.10
	Cooking electric	15372	0.92 (0.64, 1.33)	1.00 (0.83, 1.20)	0.71	0.83	0.99	3568	0.91 (0.63, 1.31)	0.94 (0.73, 1.22)	0.86
	Cooking gas	22129	1.85 (1.27, 2.70)	0.95 (0.83, 1.09)	0.00	1.88	0.94	5339	1.90 (1.23, 2.94)	0.96 (0.80, 1.15)	0.00
	Cooking gas in 1st year	13801	1.05 (0.62, 1.78)	0.91 (0.78, 1.07)	0.62	0.96	0.90	3553	1.40 (0.86, 2.28)	0.96 (0.79, 1.18)	0.17
Animals	Pets	15574	1.15 (0.91, 1.46)	0.77 (0.67, 0.88)	0.00	1.24	0.82	4087	1.06 (0.84, 1.34)	0.78 (0.65, 0.93)	0.04
	Cats	22939	1.12 (0.87, 1.44)	0.73 (0.63, 0.84)	0.00	1.22	0.73	5321	1.35 (1.02, 1.77)	0.67 (0.55, 0.81)	0.00
	Dogs	22926	1.19 (0.93, 1.53)	0.83 (0.72, 0.96)	0.01	1.33	0.87	5321	1.03 (0.81, 1.31)	0.70 (0.58, 0.85)	0.02
	Birds	18266	1.32 (0.77, 2.27)	0.87 (0.72, 1.04)	0.15	1.27	0.86	3734	0.97 (0.61, 1.54)	0.90 (0.70, 1.16)	0.81
	Cat in 1st year	15618	1.56 (1.03, 2.37)	0.83 (0.70, 0.99)	0.01	1.58	0.80	3644	0.97 (0.58, 1.61)	0.90 (0.72, 1.12)	0.79
	Dog in 1st year	15618	2.55 (1.70, 3.82)	0.80 (0.67, 0.95)	0.00	2.99	0.84	3644	1.27 (0.82, 1.97)	0.82 (0.65, 1.03)	0.08
	Bird in 1st year	15215	2.37 (1.35, 4.16)	1.06 (0.85, 1.32)	0.01	3.51	1.19	3365	1.29 (0.74, 2.27)	0.76 (0.57, 1.01)	0.10
	Farm animals	16580	1.07 (0.79, 1.44)	0.69 (0.51, 0.94)	0.05	1.22	0.77	3785	1.12 (0.85, 1.47)	0.51 (0.35, 0.76)	0.00
Diet/ weight	Meat weekly	19588	1.10 (0.82, 1.47)	0.84 (0.63, 1.12)	0.20	0.99	0.79	4281	0.94 (0.71, 1.25)	1.08 (0.73, 1.59)	0.59
	Seafood weekly	19941	0.83 (0.64, 1.08)	1.00 (0.87, 1.15)	0.23	0.77	0.96	4434	0.80 (0.60, 1.07)	1.09 (0.90, 1.33)	0.08
	Fruit weekly	18730	0.97 (0.73, 1.29)	0.88 (0.70, 1.11)	0.60	0.95	0.84	4804	1.06 (0.79, 1.42)	0.97 (0.71, 1.32)	0.67
	Green vegetables weekly	18441	1.07 (0.83, 1.38)	1.04 (0.88, 1.22)	0.85	1.04	1.07	4336	0.95 (0.73, 1.23)	1.07 (0.86, 1.33)	0.49
	Fast food weekly	13385	0.92 (0.66, 1.28)	1.13 (0.94, 1.35)	0.29	0.76	1.13	3617	0.80 (0.58, 1.10)	0.93 (0.73, 1.19)	0.47
	Fruit juice weekly	13577	0.86 (0.67, 1.09)	0.94 (0.82, 1.09)	0.50	0.68	0.95	3613	0.77 (0.58, 1.02)	0.81 (0.66, 0.99)	0.76
	Overweight	10635	1.11 (0.81, 1.51)	1.29 (1.08, 1.53)	0.41	1.39	1.32	3906	1.29 (0.97, 1.72)	0.75 (0.61, 0.93)	0.00
	Obese	10635	1.52 (0.92, 2.49)	1.30 (0.96, 1.74)	0.59	1.57	1.39	3906	1.76 (1.10, 2.83)	0.64 (0.47, 0.88)	0.00
Family/ birth	Older siblings	22216	0.79 (0.63, 0.99)	0.92 (0.82, 1.03)	0.23	0.98	0.96	4879	0.76 (0.61, 0.96)	0.92 (0.79, 1.08)	0.18
	Younger siblings	22182	0.78 (0.62, 0.98)	0.99 (0.88, 1.11)	0.07	0.79	0.95	4868	0.71 (0.56, 0.89)	1.02 (0.87, 1.19)	0.01
	More than 1 sibling	22216	0.71 (0.55, 0.91)	0.94 (0.84, 1.07)	0.04	0.92	0.98	4882	0.64 (0.50, 0.82)	0.95 (0.81, 1.12)	0.01
	Ever breastfed	22239	0.95 (0.69, 1.31)	0.91 (0.80, 1.05)	0.81	0.86	0.88	4956	0.95 (0.65, 1.40)	1.22 (1.02, 1.47)	0.25
	Low birthweight	15081	0.75 (0.46, 1.22)	1.11 (0.89, 1.39)	0.14	0.70	1.13	3648	0.73 (0.45, 1.20)	0.82 (0.61, 1.11)	0.69
	Premature	12599	1.00 (0.68, 1.47)	1.06 (0.84, 1.35)	0.80	1.17	1.05	3237	0.80 (0.51, 1.24)	0.68 (0.50, 0.91)	0.55
	Parental allergic diseases	23503	2.93 (2.36, 3.64)	2.78 (2.46, 3.13)	0.67	2.82	2.84	5068	0.90 (0.72, 1.12)	1.57 (1.33, 1.86)	0.00

	Maternal education	13907	1.31 (0.98, 1.74)	1.13 (0.86, 1.49)	0.47	1.06	1.17	3113	1.40 (1.03, 1.91)	1.39 (0.98, 1.99)	0.98
	Paternal education	14061	0.84 (0.59, 1.19)	0.89 (0.69, 1.15)	0.76	0.70	0.92	3146	0.94 (0.65, 1.36)	1.01 (0.72, 1.43)	0.76
	Any smokers	22118	0.91 (0.74, 1.12)	0.89 (0.79, 1.01)	0.87	0.98	0.86	4871	0.89 (0.71, 1.12)	0.67 (0.57, 0.79)	0.05
	Maternal smoking	23302	1.15 (0.85, 1.54)	1.03 (0.91, 1.16)	0.50	1.26	1.00	5046	1.03 (0.78, 1.36)	0.68 (0.57, 0.80)	0.01
	Maternal smoking in 1st year	20641	1.26 (0.91, 1.74)	1.14 (0.99, 1.31)	0.58	1.50	1.14	4588	0.83 (0.61, 1.12)	0.71 (0.59, 0.86)	0.42
	Maternal smoking during pregnancy	21483	1.35 (0.96, 1.90)	1.20 (1.03, 1.39)	0.53	1.47	1.20	4779	0.99 (0.72, 1.37)	0.77 (0.64, 0.94)	0.19
Air-quality	Damp	12896	1.33 (1.01, 1.76)	1.22 (0.98, 1.52)	0.64	1.55	1.22	3746	1.01 (0.77, 1.33)	0.83 (0.63, 1.10)	0.32
	Mould	11037	1.30 (0.94, 1.81)	1.26 (1.03, 1.54)	0.87	1.31	1.28	3675	1.03 (0.77, 1.38)	0.81 (0.63, 1.04)	0.22
	Damp or mould	11275	1.37 (1.06, 1.76)	1.27 (1.02, 1.57)	0.65	1.52	1.27	3800	1.11 (0.88, 1.41)	0.86 (0.66, 1.12)	0.16
	Damp in 1st year	13065	1.74 (1.29, 2.35)	1.51 (1.29, 1.76)	0.40	1.56	1.56	3340	0.94 (0.69, 1.30)	1.11 (0.91, 1.36)	0.40
	Mould in 1st year	10839	1.67 (1.11, 2.49)	1.55 (1.29, 1.87)	0.76	1.52	1.60	3085	1.06 (0.74, 1.52)	1.06 (0.84, 1.35)	1.00
	Air conditioning	10888	1.20 (0.85, 1.69)	0.95 (0.76, 1.18)	0.26	1.03	0.90	2850	1.19 (0.72, 1.96)	0.97 (0.72, 1.32)	0.51

Table S8: Results from mixed effects logistic regression models for ISAAC study only, with random intercept for centre, adjusted for age, sex, and country income group

Category (for figures)	Risk factor	Pathway 1 X to NAA		Pathway 2 X to Y		Pathway 3 Y to AA		Pathway 4 X to AA		
		n	OR (95% CI)	*n*	OR (95% CI)	*n*	OR (95% CI)	*n*	OR (95% CI)	predicted OR[a]
House/ bedding	Feather bedding	10266	0.61 (0.50, 0.75)	10828	0.88 (0.77, 1.00)	3214	0.61 (0.49, 0.76)	9926	0.50 (0.41, 0.60)	0.54
	Synthetic bedding	10266	1.35 (1.17, 1.56)	10828	1.16 (1.04, 1.30)	3214	1.59 (1.34, 1.90)	9926	1.93 (1.66, 2.25)	1.85
	Blankets	11130	1.11 (0.96, 1.29)	11657	0.89 (0.80, 1.00)	3062	0.95 (0.79, 1.14)	10671	0.82 (0.70, 0.96)	0.84
	Feather pillow	10905	0.74 (0.61, 0.91)	11428	0.90 (0.78, 1.03)	3199	0.57 (0.44, 0.74)	10501	0.44 (0.35, 0.55)	0.51
	Synthetic pillow	10199	1.06 (0.91, 1.23)	10760	1.00 (0.89, 1.11)	3185	1.35 (1.14, 1.61)	9841	1.46 (1.26, 1.69)	1.35
	Double glazing	13765	0.89 (0.77, 1.03)	15477	0.93 (0.84, 1.03)	4342	1.00 (0.84, 1.19)	13477	0.92 (0.78, 1.08)	0.93
	Double glazing in 1st year	13345	0.98 (0.84, 1.14)	14945	0.95 (0.86, 1.05)	4134	0.97 (0.81, 1.17)	13059	0.87 (0.74, 1.02)	0.92
	Urban	11926	0.80 (0.64, 0.99)	12831	1.27 (1.05, 1.54)	3433	0.71 (0.53, 0.95)	11510	0.90 (0.70, 1.14)	0.90
	Urban in 1st year	11262	0.86 (0.70, 1.05)	12173	1.11 (0.95, 1.30)	3144	1.02 (0.77, 1.35)	10853	1.09 (0.86, 1.38)	1.14
Heating/ cooking	Heating	12471	1.03 (0.83, 1.26)	14170	0.87 (0.75, 1.01)	3831	1.20 (0.94, 1.53)	12315	0.99 (0.79, 1.23)	1.04
	Wood heating	13242	1.00 (0.83, 1.21)	14702	0.92 (0.78, 1.08)	4221	1.06 (0.82, 1.37)	12945	0.95 (0.77, 1.18)	0.97
	Coal/coke heating	12675	1.09 (0.73, 1.61)	14016	1.01 (0.77, 1.30)	4005	1.72 (1.08, 2.74)	12355	1.82 (1.23, 2.71)	1.73
	Gas heating	12041	0.96 (0.80, 1.15)	13325	1.05 (0.93, 1.18)	3897	0.89 (0.73, 1.10)	11792	0.96 (0.80, 1.14)	0.94
	Heating in 1st year	11900	1.01 (0.82, 1.25)	13492	0.86 (0.74, 1.00)	3623	1.20 (0.94, 1.53)	11747	0.99 (0.80, 1.24)	1.04
	Wood heating in 1st year	12799	0.97 (0.80, 1.18)	14263	0.88 (0.75, 1.03)	4095	1.24 (0.95, 1.63)	12546	1.00 (0.81, 1.24)	1.09
	Coal/coke heating in 1st year	12385	1.16 (0.81, 1.66)	13766	0.92 (0.73, 1.15)	3945	1.39 (0.90, 2.13)	12117	1.37 (0.95, 1.98)	1.27
	Cooking electric	15082	1.07 (0.90, 1.28)	16674	1.02 (0.92, 1.14)	4608	0.94 (0.78, 1.12)	14766	0.98 (0.83, 1.15)	0.96
	Cooking gas	15868	0.96 (0.82, 1.13)	17672	0.99 (0.89, 1.10)	4950	1.14 (0.95, 1.37)	15596	1.10 (0.93, 1.29)	1.13
	Cooking gas in 1st year	11579	1.00 (0.82, 1.22)	12869	0.89 (0.77, 1.02)	3768	1.11 (0.87, 1.40)	11373	0.99 (0.81, 1.21)	0.98
Animals	Pets	9118	1.02 (0.88, 1.19)	9697	0.89 (0.79, 1.01)	2290	1.12 (0.93, 1.36)	8923	0.95 (0.80, 1.12)	1.00
	Cats	16426	1.07 (0.91, 1.26)	18523	0.82 (0.73, 0.92)	5049	1.04 (0.85, 1.26)	16296	0.78 (0.66, 0.92)	0.85
	Dogs	16424	1.14 (0.97, 1.34)	18522	0.91 (0.81, 1.03)	5049	1.01 (0.83, 1.22)	16297	0.93 (0.78, 1.10)	0.92
	Birds	16428	0.98 (0.83, 1.17)	18525	0.84 (0.74, 0.95)	5050	1.11 (0.90, 1.36)	16299	0.93 (0.77, 1.12)	0.93
	Cat in 1st year	13323	0.93 (0.75, 1.16)	15102	0.81 (0.69, 0.94)	4349	1.13 (0.88, 1.45)	13199	0.91 (0.74, 1.12)	0.91
	Dog in 1st year	13323	0.97 (0.78, 1.20)	15102	0.76 (0.64, 0.89)	4349	1.32 (1.03, 1.69)	13199	0.94 (0.76, 1.15)	1.00
	Bird in 1st year	13323	1.39 (1.12, 1.74)	15102	0.95 (0.80, 1.12)	4349	1.55 (1.21, 2.00)	13199	1.34 (1.08, 1.67)	1.47
	Farm animals	13067	1.15 (0.94, 1.42)	14153	0.73 (0.59, 0.91)	3877	1.23 (0.89, 1.69)	12662	0.83 (0.64, 1.08)	0.90
Diet/ weight	Meat weekly	15494	0.98 (0.80, 1.20)	17152	0.99 (0.84, 1.15)	4658	0.88 (0.66, 1.17)	15178	0.96 (0.75, 1.24)	0.87
	Seafood weekly	15822	0.99 (0.87, 1.12)	17482	1.04 (0.95, 1.13)	4812	0.87 (0.75, 1.02)	15532	0.95 (0.83, 1.09)	0.90
	Fruit weekly	12560	0.74 (0.61, 0.88)	13667	1.03 (0.88, 1.21)	3740	0.80 (0.62, 1.04)	12211	0.84 (0.67, 1.04)	0.83
	Green vegetables weekly	12706	0.96 (0.82, 1.13)	13840	1.02 (0.90, 1.16)	3781	1.05 (0.85, 1.30)	12359	1.03 (0.85, 1.23)	1.07
	Fast food weekly	9275	1.23 (1.06, 1.44)	9667	1.10 (0.97, 1.25)	2626	0.96 (0.78, 1.17)	8893	1.10 (0.93, 1.31)	1.05
	Fruit juice weekly	11417	1.20 (1.03, 1.41)	12576	1.08 (0.97, 1.20)	3703	0.80 (0.67, 0.96)	11149	0.88 (0.76, 1.03)	0.86
	Overweight	4120	1.31 (1.07, 1.61)	4193	1.02 (0.85, 1.22)	1574	1.29 (1.00, 1.66)	3999	1.25 (1.02, 1.54)	1.31
	Obese	4120	1.69 (1.24, 2.30)	4193	0.93 (0.66, 1.30)	1574	1.80 (1.18, 2.72)	3999	1.43 (1.04, 1.97)	1.67
Family/ birth	Older siblings	16422	1.02 (0.91, 1.15)	18269	0.93 (0.86, 1.01)	5030	1.00 (0.87, 1.14)	16109	0.85 (0.75, 0.96)	0.93
	Younger siblings	16391	1.14 (1.01, 1.28)	18214	0.95 (0.88, 1.03)	4995	1.02 (0.89, 1.17)	16075	0.97 (0.86, 1.10)	0.97
	More than 1 sibling	16425	1.14 (1.01, 1.29)	18258	0.90 (0.83, 0.98)	5019	1.12 (0.97, 1.30)	16109	0.92 (0.81, 1.05)	1.01
	Ever breastfed	16456	0.82 (0.71, 0.95)	18291	0.94 (0.85, 1.03)	5039	0.92 (0.78, 1.08)	16146	0.90 (0.78, 1.03)	0.86

	Low birthweight	13346	1.16 (0.96, 1.40)	14584	0.94 (0.82, 1.08)	4034	1.03 (0.81, 1.32)	13028	0.96 (0.77, 1.20)	0.97
	Premature	10423	1.37 (1.10, 1.71)	11254	0.86 (0.73, 1.02)	2926	1.29 (0.97, 1.72)	10118	1.08 (0.84, 1.39)	1.11
	Parental allergic diseases	17983	2.17 (1.94, 2.44)	20012	1.41 (1.30, 1.53)	5479	2.01 (1.75, 2.31)	17679	2.78 (2.46, 3.13)	2.84
	Maternal education	8546	0.76 (0.61, 0.93)	9156	0.97 (0.83, 1.14)	2067	1.15 (0.87, 1.52)	8219	1.17 (0.91, 1.51)	1.12
	Paternal education	8570	0.89 (0.73, 1.09)	9179	1.10 (0.94, 1.29)	2070	0.78 (0.60, 1.02)	8241	0.89 (0.71, 1.13)	0.86
	Any smokers	16393	1.28 (1.14, 1.44)	18222	0.84 (0.78, 0.91)	5018	1.03 (0.90, 1.17)	16092	0.85 (0.76, 0.96)	0.87
	Maternal smoking	17579	1.51 (1.33, 1.70)	19555	0.82 (0.75, 0.90)	5333	1.24 (1.07, 1.44)	17292	1.02 (0.90, 1.16)	1.02
	Maternal smoking in 1st year	14968	1.65 (1.44, 1.90)	16513	0.86 (0.77, 0.96)	4448	1.35 (1.13, 1.61)	14643	1.16 (1.00, 1.34)	1.16
	Maternal smoking during pregnancy	15755	1.59 (1.37, 1.85)	17487	0.88 (0.77, 1.00)	4776	1.33 (1.09, 1.61)	15455	1.16 (0.99, 1.37)	1.16
Air-quality	Damp	11657	1.91 (1.63, 2.25)	12817	0.87 (0.75, 1.01)	3791	1.75 (1.38, 2.20)	11346	1.62 (1.34, 1.95)	1.52
	Mould	9908	1.64 (1.36, 1.97)	10210	0.86 (0.69, 1.08)	2873	1.76 (1.29, 2.41)	9467	1.63 (1.28, 2.08)	1.52
	Damp or mould	9785	1.82 (1.55, 2.14)	10201	0.91 (0.77, 1.08)	2849	1.79 (1.39, 2.31)	9346	1.71 (1.40, 2.09)	1.63
	Damp in 1st year	11366	1.76 (1.51, 2.06)	12498	0.88 (0.76, 1.02)	3684	2.04 (1.65, 2.52)	11056	1.88 (1.58, 2.23)	1.80
	Mould in 1st year	9280	1.62 (1.34, 1.95)	9593	0.90 (0.72, 1.11)	2612	1.92 (1.44, 2.58)	8835	1.79 (1.42, 2.25)	1.73
	Air conditioning	10545	0.91 (0.75, 1.10)	11557	1.08 (0.96, 1.23)	3298	0.89 (0.72, 1.10)	10193	1.00 (0.83, 1.20)	0.96

ISAAC=International Study of Asthma and Allergies in Childhood; OR=Odds Ratio; CI=Confidence Interval; X: non-atopic, non-asthmatic; NAA: non-atopic asthma; Y:atopic, non-asthmatic; AA: atopic asthma; [a]the OR for pathway 2 multiplied by the OR pathway 3;

Table S9: Results from mixed effects logistic regression models for WASP study only, with random intercept for centre, adjusted for age, sex, and country income group

Category	Risk factor	Pathway 1 X to NAA		Pathway 2 X to Y		Pathway 3 Y to AA		Pathway 4 X to AA		
		n	OR (95% CI)	*n*	OR (95% CI)	*n*	OR (95% CI)	*n*	OR (95% CI)	predicted OR[a]
House/ bedding	Feather bedding	301	0.73 (0.42, 1.26)	190	0.67 (0.33, 1.34)	375	0.93 (0.49, 1.79)	445	0.57 (0.35, 0.93)	0.62
	Synthetic bedding	300	1.05 (0.55, 2.02)	189	1.93 (0.93, 4.02)	375	0.90 (0.50, 1.62)	444	1.27 (0.76, 2.14)	1.74
	Blankets	301	0.62 (0.38, 1.02)	190	0.67 (0.35, 1.29)	375	0.58 (0.33, 1.03)	445	0.44 (0.28, 0.68)	0.39
	Feather pillow	301	1.11 (0.68, 1.84)	190	0.52 (0.25, 1.09)	375	1.01 (0.51, 2.00)	445	1.05 (0.64, 1.74)	0.52
	Synthetic pillow	301	0.92 (0.45, 1.85)	190	1.14 (0.52, 2.53)	375	1.46 (0.79, 2.67)	445	1.11 (0.60, 2.06)	1.67
Heating/ cooking	Heating	301	0.25 (0.14, 0.44)	190	2.39 (1.13, 5.08)	376	N/A	446	N/A	N/A
	Wood heating	435	0.36 (0.16, 0.83)	237	0.52 (0.24, 1.14)	488	0.87 (0.45, 1.68)	593	0.55 (0.32, 0.97)	0.46
	Coal/coke heating	435	N/A	237	N/A	488	N/A	593	N/A	N/A
	Gas heating	111	0.62 (0.25, 1.54)	114	1.44 (0.64, 3.26)	212	1.10 (0.55, 2.22)	238	1.46 (0.80, 2.67)	1.59
	Cooking electric	188	1.91 (0.71, 5.16)	73	4.32 (0.83, 22.42)	158	0.21 (0.04, 1.04)	203	1.19 (0.37, 3.87)	0.90
	Cooking gas	300	0.42 (0.20, 0.87)	189	1.32 (0.60, 2.92)	376	0.68 (0.34, 1.34)	445	0.83 (0.47, 1.44)	0.90
Animals	Pets	432	0.72 (0.46, 1.12)	234	0.85 (0.42, 1.72)	483	0.64 (0.35, 1.16)	587	0.56 (0.36, 0.85)	0.54
	Cats	432	0.79 (0.50, 1.24)	234	1.05 (0.56, 1.95)	483	0.52 (0.30, 0.90)	587	0.65 (0.43, 0.99)	0.54
	Dogs	432	0.86 (0.56, 1.32)	234	0.71 (0.37, 1.34)	483	1.26 (0.71, 2.24)	587	0.82 (0.56, 1.21)	0.89
	Farm animals	304	0.72 (0.42, 1.25)	111	5.54 (0.66, 46.59)	179	0.11 (0.01, 0.85)	262	0.58 (0.33, 1.00)	0.59
Diet/ weight	Meat weekly	277	1.01 (0.22, 4.62)	177	0.78 (0.19, 3.15)	280	1.15 (0.35, 3.76)	351	0.74 (0.25, 2.16)	0.90
	Seafood weekly	277	1.42 (0.74, 2.72)	176	1.45 (0.69, 3.04)	280	1.09 (0.57, 2.07)	350	1.41 (0.84, 2.35)	1.58
	Fruit weekly	296	0.52 (0.13, 2.03)	186	0.28 (0.06, 1.46)	371	1.10 (0.34, 3.58)	439	0.76 (0.19, 3.04)	0.31
	Fastfood weekly	297	1.13 (0.64, 2.00)	187	1.07 (0.54, 2.12)	372	1.41 (0.78, 2.52)	441	0.94 (0.56, 1.59)	1.50
	Overweight	435	1.32 (0.82, 2.14)	237	1.02 (0.48, 2.18)	486	1.45 (0.74, 2.84)	591	1.19 (0.76, 1.87)	1.49
	Obese	435	1.94 (0.70, 5.35)	237	0.58 (0.10, 3.30)	486	2.64 (0.61, 11.54)	591	2.22 (0.88, 5.59)	1.54
Air-quality	Damp	435	0.57 (0.36, 0.89)	237	0.69 (0.34, 1.41)	488	0.87 (0.45, 1.69)	593	0.58 (0.38, 0.89)	0.60
	Mould	422	0.89 (0.58, 1.39)	231	0.97 (0.51, 1.81)	465	0.93 (0.53, 1.63)	572	0.87 (0.58, 1.29)	0.89
	Air conditioning	128	N/A	124	N/A	302	0.49 (0.12, 2.01)	324	N/A	N/A

WASP=World Asthma Phenotypes; OR=Odds Ratio; CI=Confidence Interval; X: non-atopic, non-asthmatic; NAA: non-atopic asthma; Y:atopic, non-asthmatic; AA: atopic asthma; [a]the OR for pathway 2 multiplied by the OR pathway 3;

Table S10: Results from mixed effects logistic regression models for SCAALA study only, with random intercept for centre, adjusted for age and sex

Category	Risk factor	Pathway 1 X to NAA		Pathway 2 X to Y		Pathway 3 Y to AA		Pathway 4 X to AA		
		n	OR (95% CI)	*n*	OR (95% CI)	*n*	OR (95% CI)	*n*	OR (95% CI)	predicted OR[a]
House/ bedding	Blankets	478	1.14 (0.72, 1.79)	413	0.72 (0.40, 1.30)	214	2.28 (1.17, 4.47)	403	1.67 (0.99, 2.81)	1.64
Heating/ cooking	Cooking electric	478	N/A	413	N/A	214	N/A	403	N/A	N/A
	Cooking gas	4555	0.51 (0.34, 0.76)	4416	0.49 (0.33, 0.75)	763	2.77 (0.82, 9.41)	4059	1.31 (0.40, 4.27)	1.37
	Cooking gas in 1st year	478	N/A	413	N/A	214	0.46 (0.04, 5.16)	403	N/A	N/A
Animals	Pets	4559	1.21 (1.02, 1.43)	4420	0.85 (0.71, 1.02)	763	1.52 (1.07, 2.17)	4063	1.27 (0.93, 1.73)	1.30
	Cats	4558	0.93 (0.77, 1.11)	4419	0.70 (0.57, 0.86)	762	1.99 (1.33, 2.99)	4063	1.35 (0.95, 1.90)	1.39
	Dogs	4557	1.34 (1.13, 1.58)	4419	0.95 (0.79, 1.15)	763	1.42 (0.99, 2.04)	4062	1.29 (0.95, 1.77)	1.35
	Cat in 1st year	478	2.81 (1.36, 5.78)	413	0.71 (0.23, 2.23)	214	3.59 (1.11, 11.60)	403	2.35 (1.03, 5.35)	2.56
	Dog in 1st year	478	2.13 (1.32, 3.45)	413	0.57 (0.26, 1.21)	214	4.70 (2.10, 10.51)	403	2.50 (1.44, 4.33)	2.66
	Farm animals	4076	1.07 (0.85, 1.34)	4003	1.47 (1.18, 1.83)	549	0.85 (0.51, 1.39)	3656	1.25 (0.79, 1.97)	1.25
Diet/ weight	Meat weekly	4554	1.09 (0.86, 1.38)	4415	0.80 (0.62, 1.01)	762	1.07 (0.71, 1.63)	4059	0.99 (0.68, 1.43)	0.85
	Seafood weekly	4554	0.91 (0.68, 1.22)	4415	0.99 (0.72, 1.37)	762	0.60 (0.32, 1.11)	4059	0.71 (0.44, 1.17)	0.59
	Fruit weekly	4549	0.81 (0.63, 1.04)	4410	1.54 (1.12, 2.11)	762	0.70 (0.43, 1.13)	4054	1.16 (0.78, 1.74)	1.07
	Green vegetables weekly	4548	1.05 (0.87, 1.26)	4410	1.01 (0.82, 1.23)	761	1.01 (0.70, 1.48)	4055	1.13 (0.81, 1.58)	1.02
	Fast food weekly	4546	0.94 (0.67, 1.31)	4407	1.08 (0.77, 1.51)	762	0.84 (0.45, 1.57)	4051	0.99 (0.57, 1.73)	0.91
	Fruit juice weekly	478	1.16 (0.79, 1.71)	413	1.36 (0.87, 2.12)	214	0.66 (0.38, 1.14)	403	0.98 (0.61, 1.56)	0.89
	Overweight	4461	0.89 (0.67, 1.18)	4322	1.24 (0.93, 1.64)	720	1.20 (0.74, 1.94)	3976	1.34 (0.88, 2.03)	1.48
	Obese	4461	0.81 (0.49, 1.34)	4322	1.37 (0.83, 2.28)	720	1.02 (0.48, 2.17)	3976	1.10 (0.57, 2.11)	1.39
Family/ birth	Older siblings	4554	0.92 (0.76, 1.11)	4416	1.00 (0.82, 1.24)	761	1.15 (0.78, 1.68)	4061	0.95 (0.68, 1.32)	1.15
	Younger siblings	4554	1.03 (0.85, 1.24)	4416	0.79 (0.64, 0.97)	761	0.83 (0.58, 1.19)	4061	0.69 (0.50, 0.95)	0.65
	More than 1 sibling	4554	1.01 (0.81, 1.26)	4416	0.90 (0.70, 1.15)	761	0.86 (0.56, 1.31)	4061	0.66 (0.47, 0.93)	0.77
	Ever breastfed	4504	0.75 (0.47, 1.20)	4369	0.95 (0.54, 1.67)	759	0.74 (0.30, 1.80)	4014	0.70 (0.33, 1.50)	0.70
	Premature	477	1.03 (0.50, 2.13)	412	0.86 (0.36, 2.08)	214	1.52 (0.53, 4.32)	402	1.31 (0.57, 2.98)	1.31
	Parental allergic diseases	4553	3.61 (3.02, 4.31)	4415	1.19 (0.99, 1.44)	762	2.26 (1.59, 3.22)	4057	2.67 (1.96, 3.64)	2.70
	Maternal education	4091	1.04 (0.86, 1.25)	3960	1.09 (0.89, 1.34)	695	1.01 (0.70, 1.46)	3637	1.28 (0.93, 1.77)	1.10
	Paternal education	4240	0.97 (0.79, 1.18)	4104	0.97 (0.78, 1.20)	719	0.84 (0.51, 1.37)	3769	0.79 (0.51, 1.24)	0.81
Air-quality	Any smokers	4546	1.01 (0.85, 1.20)	4408	0.98 (0.82, 1.19)	762	0.84 (0.58, 1.23)	4052	0.82 (0.58, 1.14)	0.83
	Maternal smoking	4483	1.05 (0.82, 1.35)	4349	0.95 (0.73, 1.25)	757	1.15 (0.70, 1.91)	3998	1.06 (0.68, 1.66)	1.10
	Maternal smoking in 1st year	4463	1.16 (0.89, 1.52)	4330	1.05 (0.79, 1.40)	756	1.24 (0.74, 2.08)	3978	1.19 (0.75, 1.89)	1.30
	Maternal smoking during pregnancy	4454	1.20 (0.91, 1.58)	4320	1.12 (0.84, 1.50)	755	1.31 (0.78, 2.22)	3969	1.33 (0.84, 2.11)	1.47
	Damp or mould	478	1.60 (1.07, 2.37)	413	0.92 (0.59, 1.42)	214	1.54 (0.89, 2.69)	403	1.41 (0.88, 2.26)	1.41
	Air conditioning	443	1.77 (0.11, 28.71)	379	12.63 (1.37, 116.40)	194	N/A	371	N/A	N/A

SCAALA=Social Changes, Asthma and Allergy in Latin America; OR=Odds Ratio; CI=Confidence Interval; X: non-atopic, non-asthmatic; NAA: non-atopic asthma; Y:atopic, non-asthmatic; AA: atopic asthma;
[a]the OR for pathway 2 multiplied by the OR pathway 3;

Table S11: Results from logistic regression models for ALSPAC study only, adjusted for age and sex

Category	Risk factor	Pathway 1 X to NAA		Pathway 2 X to Y		Pathway 3 Y to AA		Pathway 4 X to AA		
		n	OR (95% CI)	*n*	OR (95% CI)	*n*	OR (95% CI)	*n*	OR (95% CI)	predicted OR[a]
House/ bedding	Double glazing	2008	0.70 (0.53, 0.91)	1925	1.11 (0.80, 1.54)	716	0.90 (0.61, 1.33)	2021	1.00 (0.75, 1.33)	1.00
	Double glazing in 1st year	1999	1.03 (0.82, 1.30)	1929	1.18 (0.92, 1.51)	716	0.94 (0.69, 1.27)	2009	1.11 (0.89, 1.40)	1.11
	Urban	2034	1.05 (0.75, 1.48)	1937	1.24 (0.83, 1.86)	737	0.72 (0.45, 1.15)	2036	0.90 (0.65, 1.25)	0.90
	Urban in 1st year	1858	0.88 (0.57, 1.37)	1800	1.33 (0.77, 2.29)	689	0.63 (0.34, 1.18)	1877	0.82 (0.53, 1.26)	0.84
Heating/ cooking	Cooking gas	2015	1.03 (0.82, 1.29)	1933	1.00 (0.78, 1.28)	722	0.98 (0.72, 1.33)	2029	0.96 (0.76, 1.20)	0.98
	Cooking gas in 1st year	2017	0.80 (0.64, 1.00)	1941	1.06 (0.83, 1.35)	720	0.82 (0.61, 1.10)	2025	0.86 (0.69, 1.07)	0.86
Animals	Pets	1992	1.11 (0.86, 1.42)	1904	0.81 (0.62, 1.05)	705	0.77 (0.56, 1.05)	2001	0.62 (0.49, 0.78)	0.62
	Cats	1974	1.08 (0.85, 1.37)	1900	0.94 (0.72, 1.23)	713	0.74 (0.53, 1.04)	1993	0.70 (0.54, 0.90)	0.70
	Dogs	1966	1.27 (0.97, 1.65)	1883	0.74 (0.54, 1.03)	705	0.89 (0.59, 1.34)	1980	0.67 (0.49, 0.90)	0.66
	Birds	1948	1.14 (0.73, 1.77)	1871	1.01 (0.61, 1.68)	704	0.71 (0.37, 1.39)	1967	0.74 (0.44, 1.25)	0.72
	Cat in 1st year	2007	1.02 (0.80, 1.30)	1932	0.91 (0.70, 1.19)	720	0.89 (0.64, 1.24)	2016	0.81 (0.63, 1.04)	0.81
	Dog in 1st year	2007	1.09 (0.83, 1.43)	1932	0.64 (0.45, 0.90)	720	1.14 (0.75, 1.73)	2016	0.72 (0.53, 0.97)	0.73
	Bird in 1st year	2007	1.31 (0.84, 2.03)	1932	0.62 (0.33, 1.19)	720	0.72 (0.30, 1.72)	2016	0.45 (0.23, 0.87)	0.45
Diet/ weight	Green vegetables weekly	2035	1.11 (0.84, 1.45)	1954	0.93 (0.70, 1.23)	709	1.10 (0.77, 1.57)	2027	1.03 (0.79, 1.36)	1.02
	Fruit weekly	2031	1.61 (0.86, 3.00)	1952	1.77 (0.88, 3.59)	710	0.56 (0.25, 1.25)	2026	0.98 (0.59, 1.64)	0.99
	Fruit juice weekly	2031	1.03 (0.81, 1.30)	1951	1.00 (0.77, 1.30)	710	0.98 (0.71, 1.35)	2025	1.00 (0.78, 1.27)	0.98
	Overweight	2071	2.02 (1.52, 2.67)	1957	0.98 (0.67, 1.44)	748	1.25 (0.79, 1.97)	2069	1.24 (0.90, 1.71)	1.22
	Obese	2071	2.65 (1.60, 4.40)	1957	1.05 (0.48, 2.26)	748	1.10 (0.44, 2.79)	2069	1.14 (0.57, 2.24)	1.15
Family/ birth	Older siblings	2051	1.13 (0.91, 1.41)	1972	0.82 (0.65, 1.05)	718	1.22 (0.91, 1.65)	2046	1.01 (0.81, 1.26)	1.01
	Younger siblings	2051	0.74 (0.59, 0.92)	1972	1.32 (1.03, 1.68)	718	0.76 (0.56, 1.02)	2046	1.00 (0.80, 1.25)	1.00
	More than 1 sibling	2051	0.96 (0.76, 1.21)	1972	0.87 (0.68, 1.13)	718	1.05 (0.77, 1.44)	2046	0.93 (0.74, 1.18)	0.92
	Ever breastfed	2079	0.60 (0.47, 0.76)	1972	1.11 (0.81, 1.52)	751	0.95 (0.65, 1.40)	2079	1.05 (0.80, 1.39)	1.05
	Low birthweight	2051	1.98 (1.26, 3.13)	1944	0.87 (0.44, 1.72)	745	1.81 (0.85, 3.86)	2053	1.55 (0.94, 2.56)	1.56
	Premature	2084	1.60 (1.16, 2.21)	1972	0.76 (0.48, 1.20)	751	1.18 (0.69, 2.04)	2079	0.92 (0.63, 1.36)	0.90
	Maternal education	2055	1.00 (0.00, 0.00)	1960	1.00 (0.00, 0.00)	735	1.00 (0.00, 0.00)	2051	1.00 (0.00, 0.00)	1.00
	Paternal education	2055	1.00 (0.00, 0.00)	1960	1.00 (0.00, 0.00)	735	1.00 (0.00, 0.00)	2051	1.00 (0.00, 0.00)	1.00
Air-quality	Any smokers	1956	1.62 (1.28, 2.06)	1870	0.91 (0.68, 1.22)	696	1.32 (0.94, 1.85)	1974	1.17 (0.92, 1.50)	1.20
	Maternal smoking	2002	1.99 (1.52, 2.61)	1919	1.06 (0.74, 1.50)	715	1.12 (0.73, 1.70)	2012	1.15 (0.84, 1.56)	1.18
	Maternal smoking in 1st year	2010	1.73 (1.32, 2.26)	1933	0.70 (0.48, 1.01)	719	1.61 (1.05, 2.49)	2020	1.12 (0.84, 1.51)	1.13
	Maternal smoking during pregnancy	2061	1.88 (1.45, 2.44)	1959	0.73 (0.50, 1.06)	744	1.85 (1.21, 2.82)	2059	1.36 (1.02, 1.80)	1.34
	Damp	936	1.39 (0.76, 2.53)	870	0.98 (0.52, 1.83)	363	0.74 (0.37, 1.49)	957	0.69 (0.43, 1.12)	0.73
	Mould	972	1.52 (1.11, 2.09)	905	0.72 (0.50, 1.03)	381	1.51 (1.00, 2.30)	998	1.08 (0.80, 1.45)	1.08
	Damp or mould	934	1.21 (0.65, 2.27)	867	0.97 (0.49, 1.90)	360	0.70 (0.33, 1.47)	953	0.64 (0.39, 1.06)	0.68
	Damp in 1st year	1997	1.11 (0.88, 1.38)	1925	1.08 (0.85, 1.38)	716	1.09 (0.81, 1.46)	2009	1.16 (0.93, 1.44)	1.18
	Mould in 1st year	1994	1.29 (1.01, 1.66)	1921	0.98 (0.73, 1.30)	713	1.40 (1.00, 1.97)	2004	1.36 (1.06, 1.74)	1.37

ALSPAC= Avon Longitudinal Study of Parents and Children; OR=Odds Ratio; CI=Confidence Interval; X: non-atopic, non-asthmatic; NAA: non-atopic asthma; Y:atopic, non-asthmatic; AA: atopic asthma; [a]the OR for pathway 2 multiplied by the OR pathway 3;

Table S12: Sensitivity analysis of atopy definition (ISAAC and ALSPAC only)

Atopy definition (non-atopy remains as no reaction)	Study	Non-Atopic Non-Asthmatic (X) (%)	Atopic Non-Asthmatic (Y) (%)	Atopic Asthmatic (AA) (%)	Non-Atopic Asthmatic (NAA) (%)	Total	Correlation between pathways 1 and 3	Correlation between pathways 2 and 3
Standard[a]	ISAAC	16239 (69%)	3922 (17%)	1584 (7%)	1911 (8%)	23656	0.80 (0.67, 0.93)	-0.12 (-0.33, 0.09)
	ALSPAC	1650 (58%)	322 (11%)	433 (15%)	435 (15%)	2840		
	TOTAL	17889 (68%)	4244 (16%)	2017 (8%)	2346 (9%)	26496		
Wheal size >=5mm for any core allergen[b]	ISAAC	16239 (78%)	1777 (9%)	979 (5%)	1911 (9%)	20906	0.63 (0.45, 0.81)	-0.13 (-0.36, 0.10)
	ALSPAC	1650 (72%)	56 (2%)	141 (6%)	435 (19%)	2282		
	TOTAL	17889 (77%)	1833 (8%)	1120 (5%)	2346 (10%)	23188		
Wheal size >=4mm for at least 2 core allergens[b]	ISAAC	16239 (81%)	1164 (6%)	745 (4%)	1911 (10%)	20059	0.63 (0.45, 0.81)	-0.17 (-0.41, 0.07)
	ALSPAC	1650 (76%)	19 (1%)	80 (4%)	435 (20%)	2184		
	TOTAL	17889 (80%)	1183 (5%)	825 (4%)	2346 (11%)	22243		

ISAAC=International Study of Asthma and Allergies in Childhood; ALSPAC= Avon Longitudinal Study of Parents and Children; [a] wheal size >=3mm for ISAAC or >=2mm for ALSPAC for any allergen; [b] those who meet the standard definition but not the new definition are excluded from the sensitivity analysis.

Table S13: Sensitivity analyses by centre

Sensitivity	Non-Atopic Non-Asthmatic (X) (%)	Atopic Non-Asthmatic (Y) (%)	Atopic Asthmatic (AA) (%)	Non-Atopic Asthmatic (NAA) (%)	Total	Correlation between pathways 1 and 3	Correlation between pathways 2 and 3
Original	21920 (67%)	4870 (15%)	2642 (8%)	3309 (10%)	32741	0.81 (0.68, 0.94)	-0.06 (-0.29, 0.17)
Omitting WASP	21749 (68%)	4804 (15%)	2220 (7%)	3045 (10%)	31818	0.82 (0.69, 0.95)	-0.08 (-0.30, 0.14)
Omitting ISAAC[a]	5681 (63%)	948 (10%)	1058 (12%)	1398 (15%)	9085	0.62 (0.36, 0.88)	-0.16 (-0.42, 0.10)
Omitting SCAALA	18060 (66%)	4310 (16%)	2439 (9%)	2610 (10%)	27419	0.79 (0.66, 0.92)	-0.09 (-0.32, 0.14)
Omitting ALSPAC	20270 (68%)	4548 (15%)	2209 (7%)	2874 (10%)	29901	0.80 (0.67, 0.93)	-0.10 (-0.33, 0.13)
Controls more lenient on missing data for ISAAC and ALSPAC[b]	24482 (68%)	5616 (16%)	2642 (7%)	3309 (9%)	36409	0.85 (0.76, 0.94)	-0.15 (-0.35, 0.05)

ISAAC=International Study of Asthma and Allergies in Childhood; ALSPAC= Avon Longitudinal Study of Parents and Children; WASP=World Asthma Phenotypes; SCAALA=Social Changes, Asthma and Allergy in Latin America; [a] excludes the early life risk factors of any heating, wood heating and coal/coke heating as they were only in the ISAAC study and excludes current coal/coke heating, air conditioning, electric cooking, and any heating due to low sample size; [b] allows for the question "has the child ever been told they have asthma?" to be missing from ISAAC or for one timepoint of data on symptoms to be missing from ALSPAC.
