# Supplementary material for: Risk factors for atopic and non-atopic asthma in school-age children from high-income and low- and middle-income countries
Source: Thorax. 2025 Jun 24;80(12):e222118. doi: 10.1136/thorax-2024-222118 (PMC12703255; doi:10.1136/thorax-2024-222118)
Supplement: online supplemental file 2 [file thorax-80-12-s002.pptx]

## Slide 1
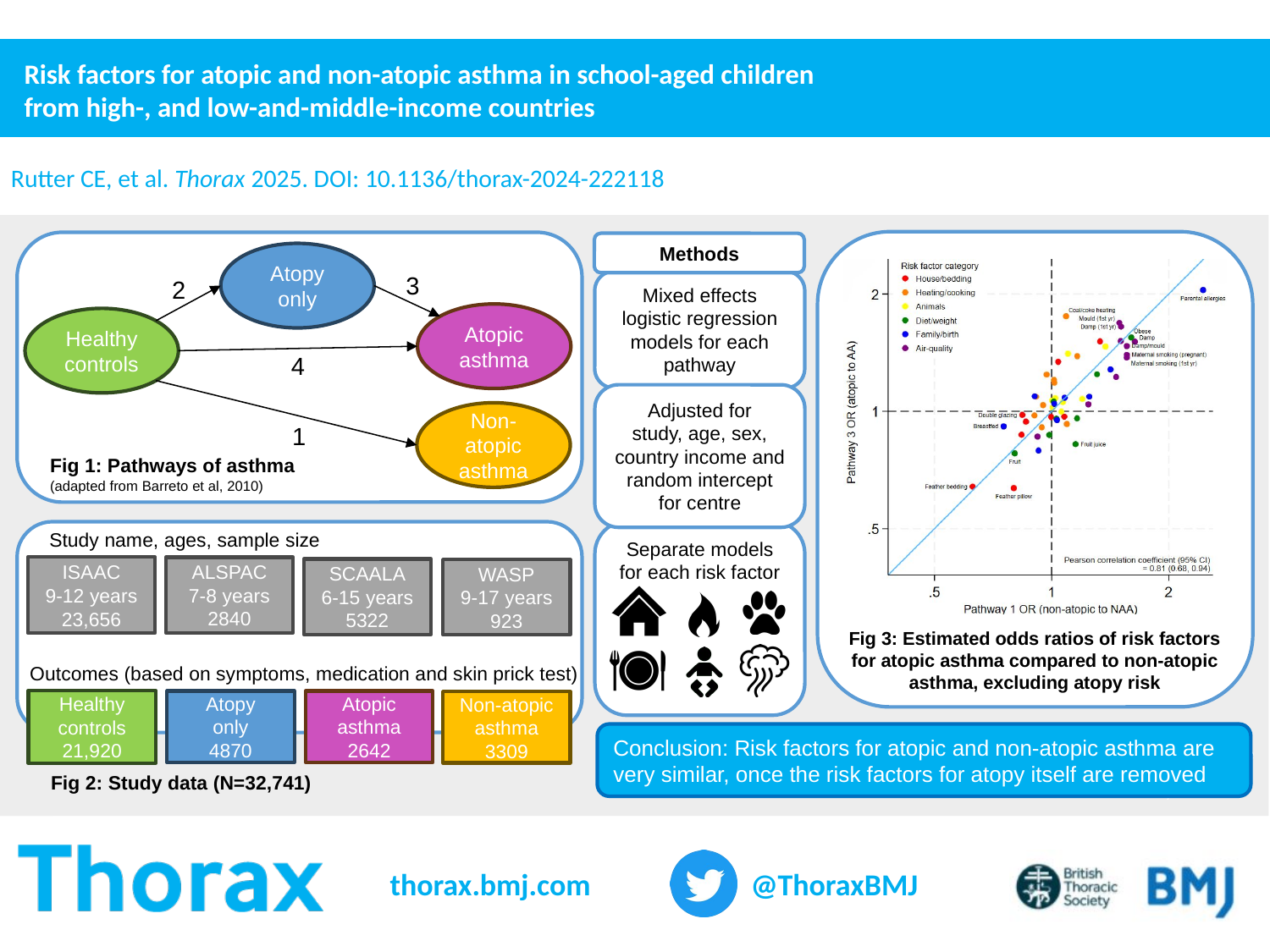

Risk factors for atopic and non-atopic asthma in school-aged children from high-, and low-and-middle-income countries
Rutter CE, et al. Thorax 2025. DOI: 10.1136/thorax-2024-222118
Methods
Atopy only
3
2
Mixed effects logistic regression models for each pathway
Atopic asthma
Healthy controls
4
Adjusted for
study, age, sex, country income and random intercept for centre
Non-atopic asthma
1
Fig 1: Pathways of asthma
(adapted from Barreto et al, 2010)
Study name, ages, sample size
Separate models for each risk factor
ISAAC
9-12 years
23,656
ALSPAC
7-8 years
2840
SCAALA
6-15 years
5322
WASP
9-17 years
923
Fig 3: Estimated odds ratios of risk factors for atopic asthma compared to non-atopic asthma, excluding atopy risk
Outcomes (based on symptoms, medication and skin prick test)
Healthy controls
21,920
Atopy
only
4870
Atopic asthma
2642
Non-atopic asthma
3309
© Author(s) (or their employer(s) 2019. Re-use permitted under CC BY. Published by BMJ.
Conclusion: Risk factors for atopic and non-atopic asthma are very similar, once the risk factors for atopy itself are removed
Fig 2: Study data (N=32,741)
thorax.bmj.com @ThoraxBMJ
